# Understanding Substrate Selectivity of Phoslactomycin Polyketide Synthase by Using Reconstituted in Vitro Systems

**DOI:** 10.1002/cbic.202000112

**Published:** 2020-03-30

**Authors:** Kyra Geyer, Srividhya Sundaram, Peter Sušnik, Ulrich Koert, Tobias J. Erb

**Affiliations:** ^1^ Department of Biochemistry and Synthetic Metabolism Max Planck Institute for Terrestrial Microbiology Karl-von-Frisch-Str. 10 35043 Marburg Germany; ^2^ Department of Chemistry Philipps-University Marburg Hans-Meerwein-Str. 4 35032 Marburg Germany; ^3^ LOEWE Center for Synthetic Microbiology (Synmikro) Karl-von-Frisch-Str. 16 35043 Marburg Germany

**Keywords:** acyltransferases, enzymes, natural products, phoslactomycin, polyketides

## Abstract

Polyketide synthases (PKSs) use simple extender units to synthesize complex natural products. A fundamental question is how different extender units are site‐specifically incorporated into the growing polyketide. Here we established phoslactomycin (Pn) PKS, which incorporates malonyl‐ and ethylmalonyl‐CoA, as an *in vitro* model to study substrate specificity. We combined up to six Pn PKS modules with different termination sites for the controlled release of tetra‐, penta‐ and hexaketides, and challenged these systems with up to seven different extender units in competitive assays to test for the specificity of Pn modules. While malonyl‐CoA modules of Pn PKS exclusively accept their natural substrate, the ethylmalonyl‐CoA module PnC tolerates different α‐substituted derivatives, but discriminates against malonyl‐CoA. We show that the ratio of extender transacylation to hydrolysis controls incorporation in PnC, thus explaining site‐specific selectivity and promiscuity in the natural context of Pn PKS.

## Graphical abstract

**Going to any length**: Polyketide synthases produce compounds of high structural and functional diversity. We have created phoslactomycin polyketide synthase‐derived *in vitro* systems and studied their substrate selectivity. We were able to produce various phoslactomycin derivatives with altered side‐chain lengths and characterized the transacylation and hydrolysis activity of the involved acyltransferases for all substrates tested.

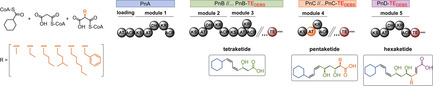

## Introduction

Polyketides are a class of natural products with high structural diversity but common biosynthetic logic. They are synthesized by polyketide synthases (PKSs) from simple acyl‐ and (alkyl)malonyl‐CoA‐based building blocks. The structure of the product is determined by the type and location of catalytic domains in the assembly line, while structural diversity of the backbone arises from the choice of extender unit, extent of reduction and stereo centers installed during biosynthesis.^1^, ^2^

**Scheme 1:**
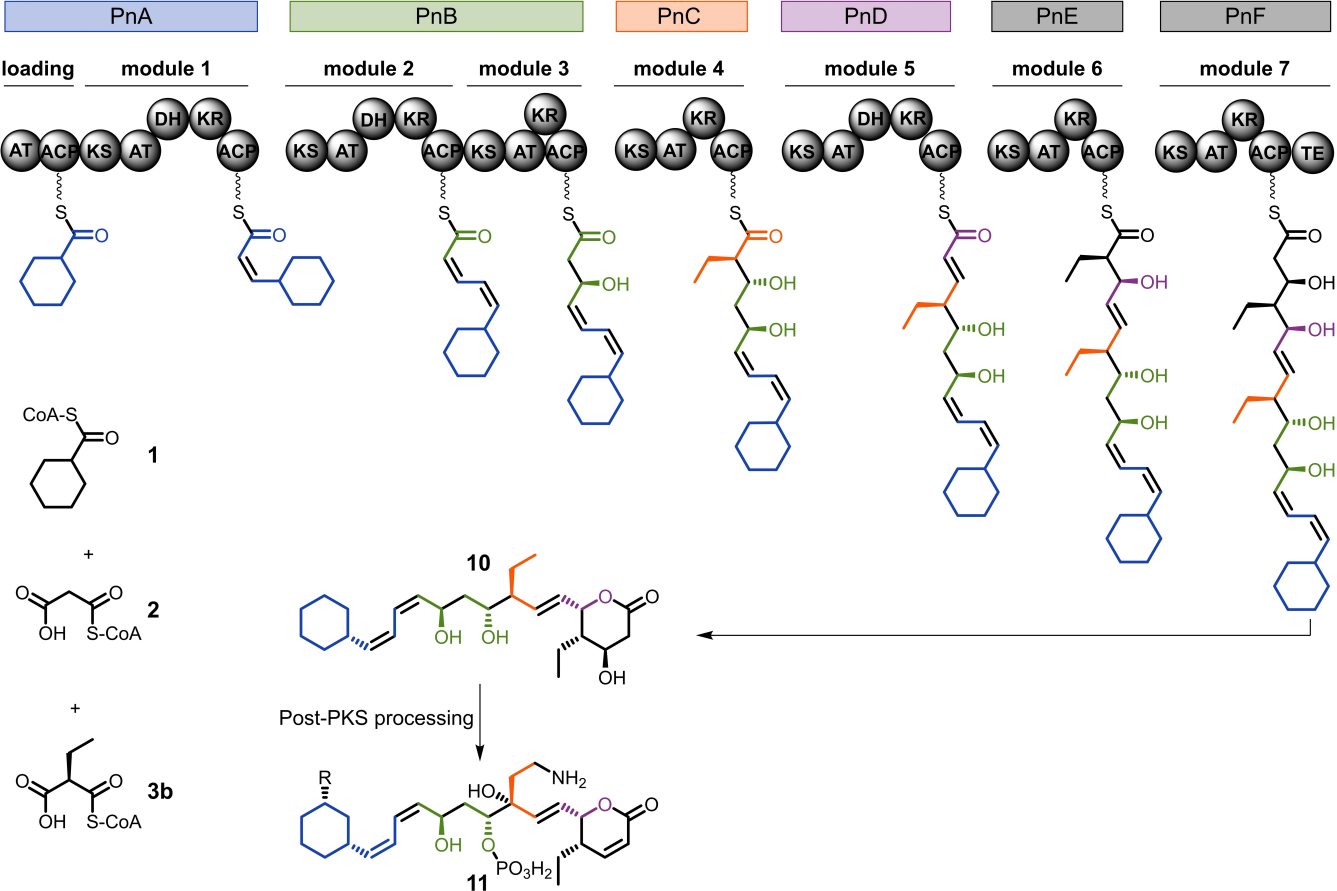
Phoslactomycin polyketide synthase Pn PKS. ACP=acyl carrier protein; AT=acyltransferase; DH=dehydratase; KR=ketoreductase; TE=thioesterase; **1**=cyclohexanecarboxyl‐CoA; **2**=malonyl‐CoA; **3**=(2*S*)‐ethylmalonyl‐CoA; **10**=phoslactomycin polyketide backbone, **11**=bioactive phoslactomycin derivatives; R=isobutyryloxy, isovaleryloxy, 4‐methylcaleryloxy, cyclohexylcarbonyloxy, 4‐methylheptanoyloxy.^9^, ^10^

The three major extender units incorporated in polyketides are malonyl‐, methylmalonyl‐CoA and to a lesser extent ethylmalonyl‐CoA. While it is generally believed that the acyltransferase (AT) domains control extender unit incorporation, recent reports have shown that these domains are more promiscuous than initially assumed. For example AT5 of monensin accepts methyl‐ and ethylmalonyl‐CoA in the natural system, but experiments showed that also propargyl‐ and butylmalonyl‐CoA are accepted.^3^, ^4^ Research on the 6‐deoxyerythronolide B PKS (DEBS), a well‐established model system, revealed an intrinsic promiscuity of the ATs of module 2, module 3^5^ and module 6.^6^ Promiscuous AT domains were also observed in the structurally highly similar pikromycin PKS (module 5 and module 6^7^, ^8^). However, to fundamentally understand how extender unit selectivity in PKS is achieved other, more diverse model systems are required. Especially, since the well‐studied DEBS PKS does not need to distinguish between different extender units, because only methylmalonyl‐CoA is incorporated into the assembly line. Here, we focused on phoslactomycin (Pn) PKS, a modular type I PKS from *Streptomyces platensis*.^9^, ^10^ Pn PKS is composed of a loading module selecting cyclohexanecarboxyl‐CoA (**1**) and seven extending modules of which five incorporate malonyl‐CoA (**2**) and two incorporate ethylmalonyl‐CoA (**3** **b**; Scheme 1). This makes Pn PKS, in comparison to methylmalonyl‐CoA specific DEBS PKS, an excellent model to study how extender unit selectivity is controlled at different sites within one PKS.

## Results and Discussion

**Scheme 2:**
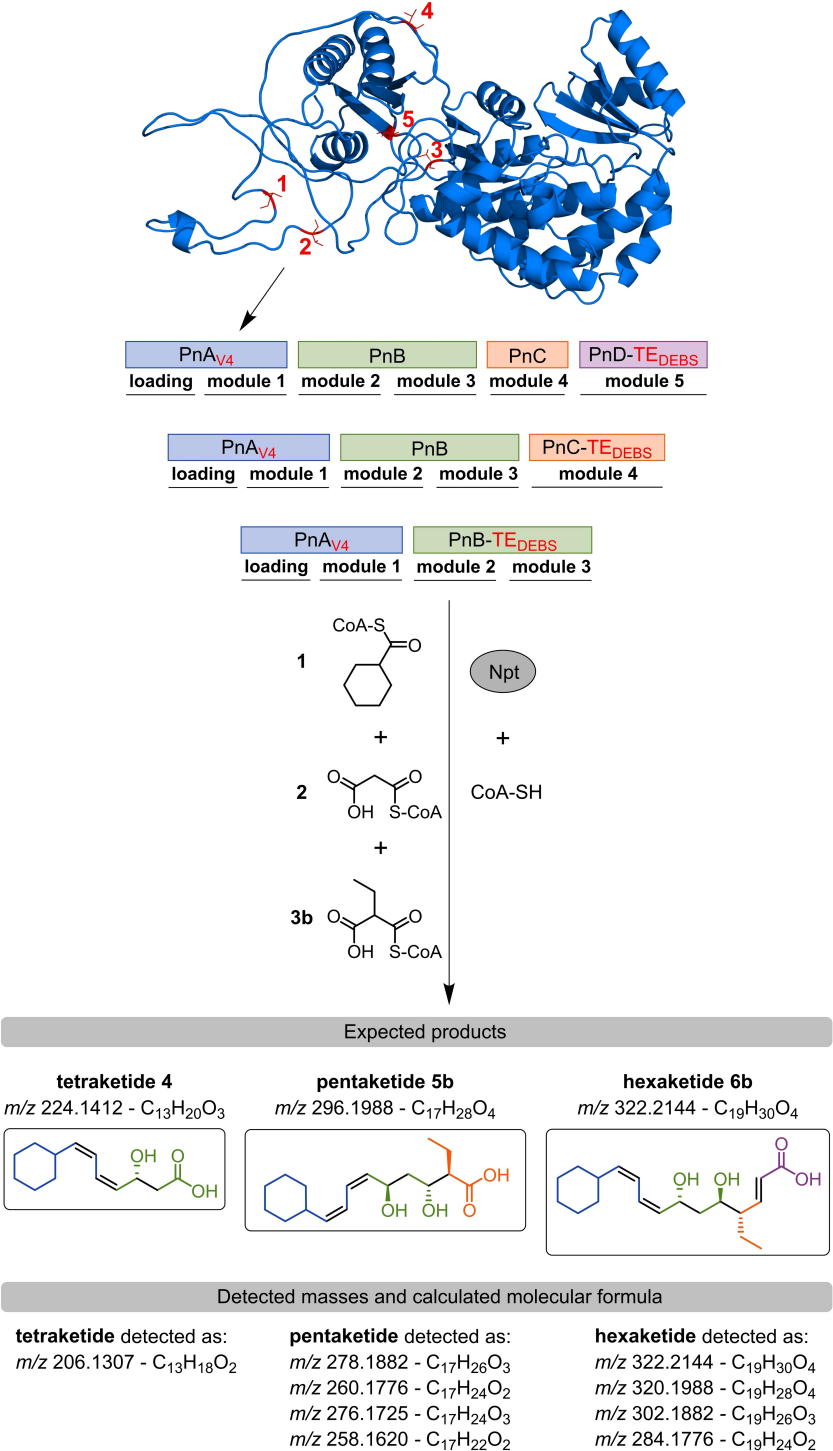
*In vitro* reconstitution of Pn PKS. Structural model of PnA loading AT with the alternative transcription start sites V1 to V5 highlighted in red. *S. platensis* endogenous Ppant transferase Npt and CoA‐SH were used to ensure full conversion into the corresponding holo‐PKS *in vitro*. Compounds **1**, **2** and **3** **b** are the natural substrates of Pn PKS. Termination of polyketide production with PnB‐TE_DEBS_, PnC‐TE_DEBS_ or PnD‐TE_DEBS_ is expected to yield the depicted tetra‐ (**4**), penta‐ (**5** **b**) or hexaketide (**6** **b**). Mass spectrometric analysis detected derivatives thereof indicating dehydrations and oxidations. A detailed discussion of the individual masses, the corresponding putative products and their generation is provided in Figure S6.

We aimed at establishing different Pn‐based minimal model systems for the production of tetra‐, penta‐ and hexaketides to study promiscuity and selectivity, in particular of the **3** **b** incorporating PnC_AT. To that end, we expressed PnA‐PnD as individual proteins in *E. coli* BAP1^7^, ^8^, ^11^, ^12^ (Figure S1 in the Supporting Information). For PnA production, we tested five different transcription start sites (PnA_V1_‐PnA_V5,_ Scheme 2), due to insolubility upon expression of the full‐length annotated PnA protein. Variant PnA_V4_ resulted in highest protein expression and solubility. To enable polyketide release in the tetra‐, penta‐ and hexaketide systems, we used the chain releasing thioesterase (TE) from DEBS to create chimeric PnB‐TE_DEBS_, PnC‐TE_DEBS_ and PnD‐TE_DEBS_ constructs^8^, ^13^, ^14^ (information on the DNA sequence is given in the Supporting Information).

*E. coli* BAP1 encodes for a functional phosphopantetheinyl (Ppant) transferase. Yet, phosphopantetheinylation highly varied across individual Pn acetyl‐carrier proteins (ACPs; Table S1). While PnC_ACP was fully converted to *holo‐*ACP (96 %), only 26 % of PnB_ACP1 was present in the *holo‐*form. Analysis revealed one gene from the 4’‐phosphopantetheinyl transferase superfamily (Npt, OSY40025) with low homology to Sfp (16.1 % sequence identity) in the genome of *S. platensis*. We expressed the protein in *E. coli* BL21 (DE3) and demonstrated that Npt serves as a bona fide Ppant transferase that is able to activate *apo‐*Pn_ACPs (Figure S2). We further added purified Npt to all *in vitro* assays to ensure the full conversion of all Pn proteins to their *holo‐*form.

One‐pot reaction mixtures of PnA_V4_ and PnB‐TE_DEBS_ (tetraketide system), PnA_V4_, PnB and PnC‐TE_DEBS_ (pentaketide system), PnA_V4_, PnB, PnC and PnD‐TE_DEBS_ (hexaketide system), incubated with their natural substrates resulted in production of respective polyketide derivatives, as analyzed by high resolution LC‐MS. Enzymatic production of these derivatives was additionally verified using 2‐C^13^ labelled malonyl‐CoA (Figures S3, S4 and S5). A product corresponding to a dehydrated tetraketide (*m/z* 206.1307, formula C_13_H_18_O_2_) is the exclusive product found in tetraketide assays. In pentaketide assays, a more complex product spectrum was observed, with *m/z* 258.1620 corresponding to a pentaketide of formula C_17_H_22_O_2_ as the main product. Various masses were found in hexaketide assays that correspond to different products with *m/z* 284.1776 corresponding to hexaketide of formula C_19_H_24_O_2_ as the main product (Scheme 2). Based on the combined information from the high resolution mass spectrometer and isotopic labeling, we propose structures for the individual masses detected, and provide an explanation for their generation in Figure S6. Altogether, our analysis demonstrated that the Pn PKS enzymes are functional *in vitro* and can be combined stepwise to produce different truncated Pn polyketide variants (Scheme 2 and Figure S6)

**Scheme 3:**
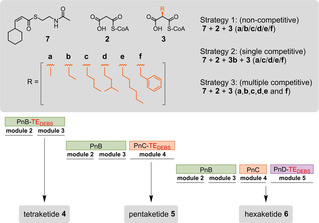
Pn PKS *in vitro* characterization. Diketide product **7** of PnA_V4_ as SNAC thioester analogue, **2** malonyl‐CoA, **3** (*2S*)‐acyl‐malonyl‐CoA derivatives with residues marked in orange: **a** methyl‐, **b** ethyl‐, **c** butyl‐, **d** 3‐methylbutyl‐, **e** hexyl‐, **f** benzylmalonyl‐CoA. Polyketide production is terminated with PnB‐TE_DEBS_, PnC‐TE_DEBS_ or PnD‐TE_DEBS_ to produce **4**, **5** and **6**, respectively.

Successful reconstruction of the first six modules of Pn PKS allowed us to assess the substrate tolerance of the system with seven different extender units (**2**, **3** **a‐f**). In these assays, we bypassed PnA_V4_ by using the *N*‐acetylcysteamine (SNAC) analogue of the diketide product (**7**, see the Supporting Information for synthesis). We tested substrate tolerance across the tetra‐, penta‐ and hexaketide systems employing three different strategies. In strategy 1, we used **7**, **2** (malonyl‐CoA) and one of the six α‐substituted extender units (**3** **a‐3** **f**) to test if substrates other than ethylmalonyl‐CoA (**3** **b**) are incorporated. In strategy 2, we used **7**, **2**, **3** **b** and one additional extender unit (**3** **a**, **3** **c‐3** **f**), to set the incorporation of **3** **b** in direct competition to an alternative non‐native extender unit. In strategy 3, we used **7**, **2**, and all six α‐substituted extender units in parallel (**3** **a‐f**) to allow identification of the preferred substrate(s) (Scheme 3).

**Figure 1:**
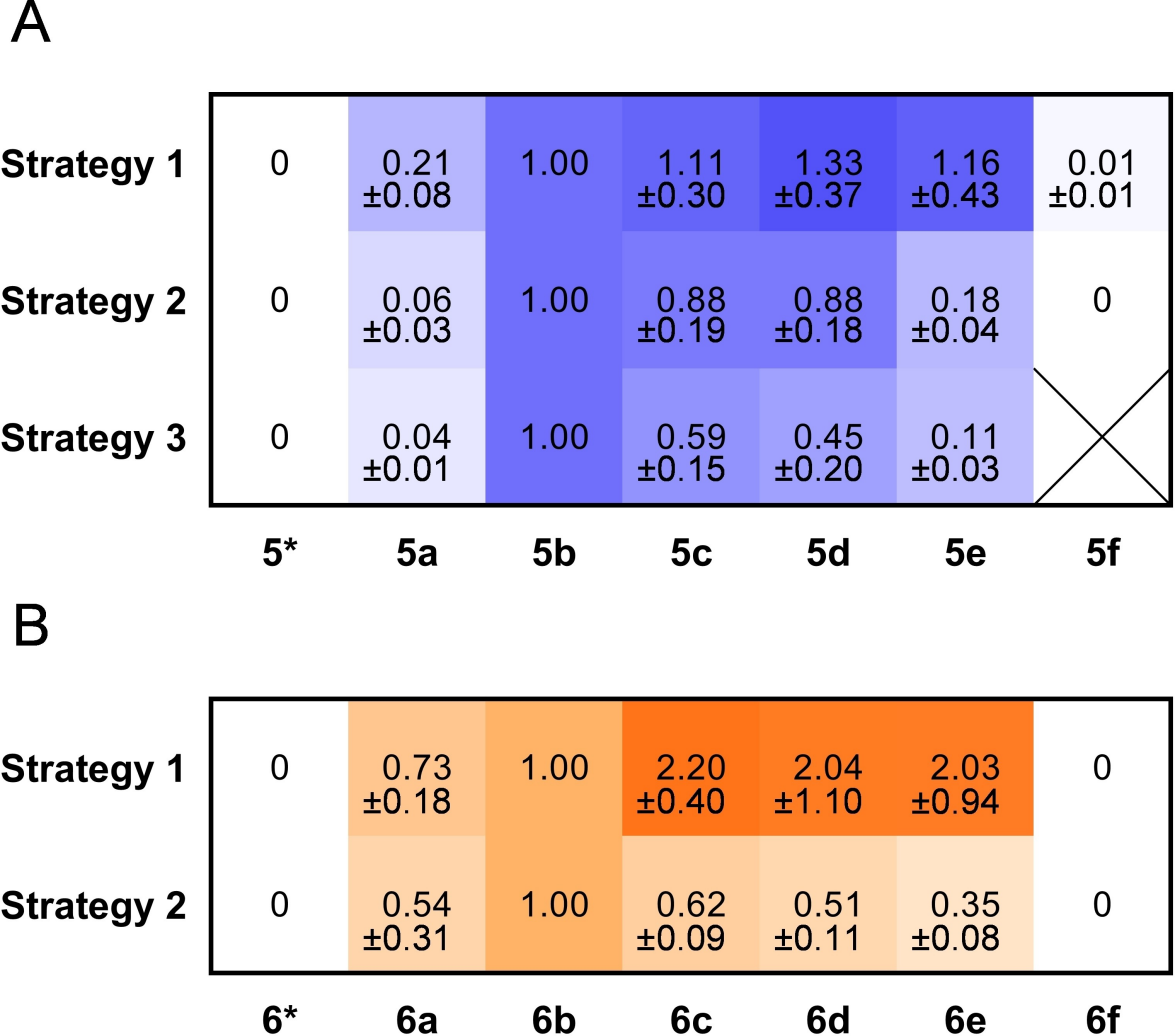
Relative amounts of penta‐ and hexaketides in competition assays. The ion count of each product is set relative to **5** **b** or **6** b. 5* or 6* indicate incorporation of **2** by PnC. The mean of a minimum of three biological replicates is given, with each two technical replicates (pentaketide assays) and the mean of three biological replicates, with each two technical replicates (hexaketide assays).

Using strategy 1 and strategy 2 with the tetraketide system, we observed exclusive incorporation of 2, as indicated by product analysis. No alternative extender unit was accepted, indicating exquisite selectivity by PnB. In the pentaketide system, incorporation of all six α‐substituted extender units **3** **a‐3** **f** was observed, while **2** was not incorporated at the position of PnC (Figure S4). This demonstrates substrate tolerance of PnC towards α‐substituted extender units, but at the same time strong discrimination against **2**. More specifically, similar amounts of **5** **b**, **5** **c**, **5** **d** and **5** **e** were detected in the pentaketide system, when testing substrate tolerance with strategy 1 (Figure 1A). Products of **3** **a** incorporation showed less intensity and products of **3** **f** incorporation were detectable in traces. In strategy 2, when we put incorporation of **3** **b** (natural substrate) in direct competition to another extender unit, we again observed tolerance of PnC towards medium chain extender units (**3** **b‐d**), and discrimination against short (**3** **a**) and long chain (**3** **e** and **3** **f**) extender units. With strategy 3, the trend increased further towards preferred incorporation of **3** **b** (natural substrate) and **3** **c** by the pentaketide system.

Having demonstrated that PnC contains a highly promiscuous AT and is able to form different pentaketides by incorporation of **3** **a‐3** **f**, we asked, whether PnD is able to accept and process the unnatural pentaketides. We used strategy 1 and 2 to study substrate tolerance of the reconstituted hexaketide system. When only one α‐substituted substrate is available, **6** **a**‐**6** **e** but not **6** **f** were formed (Figures 1B and S5). Also, we did not detect multiple incorporations of non‐native extender units, indicating that PnC is the only entry point of non‐native extender units. With strategy 2, natural product **6** **b** became the main product. This demonstrated that Pn PKS is generally flexible for incorporation of unnatural extender units at the level of PnC (Figure 1A, strategy 1) but that in presence of the natural substrate a preference for the latter exists (Figure 1B, strategy 2).

When comparing the product profile of strategy 2 to strategy 1 in hexaketide assays, we observed that relative product formation decreased with increasing size of the side chain. For **6** **a** we observed a decrease of only 26 %, whereas for **6** **c**, **6** **d** and **6** **e** the relative decrease increased between 72 % and 83 %. Furthermore, compared to **5** **a**‐**5** **b** and **6** **a**‐**6** **b** that were detected as reduced product, alongside oxidized products (Figure S6, Table S2), Compounds **5** **c**‐**5** **f** and **6** **c**‐**6** **e** were exclusively found as the non‐reduced products, indicating the polyketide is not fully processed by subsequent domains after incorporation of the non‐native, longer extender unit, which is in line with earlier reports.^8^ Taken together this indicates a selection by PnD for pentaketides with shorter site chains, either due to restrictions in the KS active site and/or by preferred reduction (and further processing) of those pentaketides.

Next, we wanted to study the specificity of acyl transfer at single domain level. To that end, we aimed at measuring kinetic parameters for transacylation and hydrolysis in ketosynthase‐AT (KS‐AT) didomains. In the commonly used assay, these kinetic data are collected by coupling CoA‐SH release by the AT domain to production of succinyl‐CoA by a commercially available α‐ketoglutarate dehydrogenase complex (α‐KGDH).^12^, ^15^, ^16^ However we observed varying apparent (app.) AT activity that was dependent on the α‐KGDH batch used. We therefore developed a steady state kinetic assay based on purified *E. coli* succinyl‐CoA ligase complex SucC/SucD, to minimize AT‐inhibition (Scheme S1). In this assay, when the acyl‐residue is transferred to the AT, free CoA‐SH is released. The free CoA‐SH is coupled to succinate via SucC/D, whereby ADP is released. ADP production is subsequently coupled to NADH consumption via pyruvate kinase/lactate dehydrogenase. Thus, the release of CoA‐SH is coupled to NADH consumption, which can be spectrophotometrically measured and quantified.

With the SucC/SucD‐based assay we determined kinetic parameters of PnB_KS‐AT1, PnB_KS‐AT2, PnC_KS‐AT and PnD_KS‐AT for hydrolysis and transacylation (Table S3, Figure S7). As negative control AT catalytic knockouts were used. All ATs described to incorporate **2** (PnB module 2, module 3 and PnD), showed hydrolysis and transacylation activity only for their natural substrate, which is in line with our *in vitro* experiments in the reconstituted system. These measurements were independently confirmed by mass spectrometric analysis of acylated ACPs, in which we exclusively detected malonyl‐ACP formation (Tables S1 and S4). All three ATs showed comparable hydrolysis rates at an app. *k*_cat_ of approx. 3 min^−1^ and an app. *K*_M_ between 4 μM to 11 μM. Rates for transacylation were between 10‐ to 35‐ fold higher (app. *k*_cat_ of 28 min^−1^–140 min^−1^) at 2.5 to 7.7 fold higher catalytic efficiency (Table S3).

In contrast, PnC_KS‐AT showed pronounced promiscuity and, except for **3** **f**, hydrolytic activity was detected with all tested extender units, including **2** which is not incorporated into the pentaketide (Table 1). The app. *k*_cat_ and in particular the app. *K*_M_ strongly differed, depending on the tested substrate. Interestingly, the app. *k*_cat_ for **2**, **3** **a**‐**3** **e** differed only by a factor of four, ranging between 0.4 min^−1^ to 1.4 min^−1^, while strong differences were observed in the app. *K*_M_, that varied by a factor of 12 between the different substrates, ranging from 6 μM to 73 μM. Transacylation rates of PnC_KS‐AT could not be measured with the SucC/SucD‐based assay, due to high background activity, which could not be reduced even by further purification of the enzymes. Thus, we determined the app. *k*_cat_ for transacylation for **2** and **3** **b**‐**3** **e** by an HPLC‐based assay (Figures S8, S9 and S10). PnC_KS‐AT was able to transfer all tested substrates, however at lower rates compared to transacylation rates of PnB_KS‐AT1, PnB_KS‐AT2 and PnD_KS‐AT. The highest transacylation rates of PnC_KS‐AT were observed for its natural substrate **3** **b** (4±0.1 min^−1^) The slightly longer substrates **3** **c** and **3** **d** were transacylated at approximately 7‐ to 12‐ fold and **2** and **3** **e** at approximately 100‐ to 80‐ fold lower rates, respectively, suggesting that transacylation (more than hydrolysis) is a crucial factor for incorporation by PnC. We independently confirmed these findings by mass spectrometric analysis of acylated PnC_ACP (Tables S1 and S4).

**Table 1:** Apparent *K*_M_ and app. *k*_cat_ values for hydrolysis and transacylation by PnC_KS‐AT.

Substrate	*K*_M_ [μM]	*k*_cat_ [min^−1^]	*k*_cat_/*K*_M_
**Hydrolysis**			
2	53±41	0.6±0.15	0.01
3a	73±33	1.4±0.3	0.02
3b	6±1.8	0.9±0.06	0.15
3c	6.2±1.4	0.7±0.04	0.11
3d	20±6.7	0.8±0.08	0.04
3e	19±15.5	0.4±0.08	0.02
3f	–^[a]^	–	–
**Transacylation**			
2	n.d^[b]^	0.04±0.002	–
3a	n.d	n.d	–
3b	n.d	4±0.1	–
3c	n.d	0.6±0.03	–
3d	n.d	0.3±0.07	–
3e	n.d	0.05±0.001	–
3f	n.d	–	–

[a] Not detected. [b] not determined.

Overall, our kinetic data show that malonyl‐CoA specific domains (PnB_KS‐AT1, PnB_KS‐AT2, and PnD_KS‐AT) exclusively transfer malonate to their cognate ACP and neither show transacylation nor hydrolysis for non‐native substrates. PnC_KS‐AT on the other hand transfers all tested substrates onto its cognate ACP, including **2** which is not incorporated into the polyketide product. PnC_KS‐AT also displays hydrolytic activity against all substrates, indicating that in the natural context selectivity is achieved by the ratio of transacylation and hydrolysis rate in PnC.

## Conclusions

In this study, we successfully reconstituted the first six modules of phoslactomycin PKS as a new *in vitro* model system to assess how different extender units are selectively recruited within one PKS system. Establishing this model system required production of various soluble and functional proteins, as well as their respective substrates. *C*‐terminal chimeric fusions of DEBS_TE to PnB, PnC and PnD enabled production of different polyketide products ranging from tetra‐ to hexaketide (Scheme 2), to study incorporation selectivity of natural and non‐native extender units in non‐competitive and competitive assays.

Experiments on substrate selectivity show that PnB module 2, module 3 and PnD exclusively accept **2**, even in the presence of alternative extender units, demonstrated by *in vitro* reconstitution and kinetic assays. PnC on the other hand shows a high substrate tolerance towards α‐substituted malonyl‐CoA derivatives but does not accept **2** itself, which is present in the cell. Strikingly PnC mainly excludes substrate **3** **a**, while it readily accepts its natural substrate **3** **b** and longer derivatives (**3** **c**‐**e**; Figure 1A, strategy 1). Competition assays show that PnC prefers ethyl‐residues over shorter (methyl‐) and very long (hexyl‐) residues, while in competition PnC does not have a pronounced selectivity against linear and branched medium chain lengths (butyl‐ and 3‐methylbutyl residues; Figure 1A, strategies 2 and 3). The same competition assays also indicate that PnD readily accepts unnatural polyketide intermediates from PnC_ACP, with a preference of shorter side chains at C‐2 position (Figure 1B). The preference of PnD for short side chains at C‐2 position could be located at the KS and ketoreductase, as previously hypothesized.^8^ Thus, the observed trends in product distribution are the result of medium chain length preference of PnC during extender unit incorporation and selection for short(er) side chains at the C‐2 position of the polyketide by PnD during downstream processing.

Detailed kinetic analysis shed light on the question of how PnC discriminates against malonate. Hydrolysis rates of PnC_KS‐AT between different substrates differ only by a factor of four. Note that hydrolysis of natural substrate **3** **b** is even two‐fold higher than for **2**. Assuming saturating levels of malonyl‐CoA on PnC_KS‐AT this suggests that substrate preference is guided by differences in transacylation rates of **3** **b** and **2**. Transacylation of the natural substrate **3** **b** is 100‐fold higher than **2**. The relative transacylation to hydrolysis rate still shows a 63‐fold preferred reaction for **3** **b** (4.4) versus **2** (0.07). The relative transacylation to hydrolysis rate also explains incorporation of the other substrates in following order **3** **c** (0.82), **3** **d** (0.41) and **3** **e** (0.142) that we observed in the pentaketide system. While the hydrolytic side reaction is often referred to as a proofreading function,^7^ it is apparently not the hydrolysis per se in PnC_KS‐AT that discriminates against a substrate, but rather the efficiency of transacylation. Apparently, PnC_KS‐AT was selected against transacylation of malonyl‐CoA, which is of importance in natural context in respect to formation of the correct product. The observed flexibility in transacylation (and incorporation) of non‐native extender units in PnC_AT and its downstream processing is probably due to the fact that these are not present in the natural context.

Substrate selectivity is also linked to unique sequence motifs. A common motif in AT domains is GX_1_SX_2_G around the active site serine, whereas X_1_ mostly is a histidine.^17^ Another important motif is a highly conserved HAFH motif in the binding pocket of malonyl‐CoA specific domains. Ethylmalonyl‐CoA specific ATs show a less well preserved motif at this position, generally XAGH, with X being F, T, V or H.^18^ On both positions PnC_AT shows unique sequence motifs, with a GSS and a CASH motif in the acyl‐CoA binding pocket (Figure S11) These sequence differences provide strong arguments for the observed substrate tolerance and render PnC_AT a highly interesting enzyme for further functional investigations.

In sum, we established an additional, complementary *in vitro* PKS system for the detailed study of substrate specificity. This system will hopefully enable the testing of existing hypotheses on extender substrate selectivity and facilitate PKS engineering by offering the possibility of site specific incorporation of alternative extender units to create novel polyketide structures in the future.

## Conflict of interest

The authors declare no conflict of interest.

## Supporting information

As a service to our authors and readers, this journal provides supporting information supplied by the authors. Such materials are peer reviewed and may be re‐organized for online delivery, but are not copy‐edited or typeset. Technical support issues arising from supporting information (other than missing files) should be addressed to the authors.

Supplementary

## References

[1] J. Staunton , K. J. Weissman , Nat. Prod. Rep. 2001, 18, 380–416. 11548049 10.1039/a909079g

[2] C. Hertweck , Angew. Chem. Int. Ed. 2009, 48, 4688–4716; 10.1002/anie.200806121 19514004

[3] M. Oliynyk , C. B. Stark , A. Bhatt , M. A. Jones , Z. A. Hughes-Thomas , C. Wilkinson , Z. Oliynyk , Y. Demydchuk , J. Staunton , P. F. Leadlay , Mol. Microbiol. 2003, 49, 1179–1190. 12940979 10.1046/j.1365-2958.2003.03571.x

[4] A. Ismail-Ali , E. K. Fansa , N. Pryk , S. Yahiaoui , S. Kushnir , M. Pflieger , A. Wittinghofer , F. Schulz , Org. Biomol. Chem. 2016, 14, 7671–7675. 27452503 10.1039/c6ob01201a

[5] B. Lowry , T. Robbins , C. H. Weng , R. V. O′Brien , D. E. Cane , C. Khosla , J. Am. Chem. Soc. 2013, 135, 16809–16812. 24161212 10.1021/ja409048k PMC3858836

[6] I. Koryakina , J. B. McArthur , M. M. Draelos , G. J. Williams , Org. Biomol. Chem. 2013, 11, 4449–4458. 23681002 10.1039/c3ob40633d

[7] S. A. Bonnett , C. M. Rath , A. R. Shareef , J. R. Joels , J. A. Chemler , K. Hakansson , K. Reynolds , D. H. Sherman , Chem. Biol. 2011, 18, 1075–1081. 21944746 10.1016/j.chembiol.2011.07.016 PMC3184853

[8] E. Kalkreuter , J. M. CroweTipton , A. N. Lowell , J. Am. Chem. Soc. 2019, 141, 1961–1969. 30676722 10.1021/jacs.8b10521 PMC6556384

[9] S. Fushimi , K. Furihata , H. Seto , J. Antibiot. (Tokyo) 1989, 42, 1026–1036. 2753809 10.7164/antibiotics.42.1026

[10] Y. L. Chen , J. Zhao , W. Liu , J. F. Gao , L. M. Tao , H. X. Pan , G. L. Tang , Gene 2012, 509, 195–200. 22940146 10.1016/j.gene.2012.08.030

[11] B. A. Pfeifer , S. J. Admiraal , H. Gramajo , D. E. Cane , C. Khosla , Science 2001, 291, 1790–1792. 11230695 10.1126/science.1058092

[12] B. J. Dunn , K. R. Watts , T. Robbins , D. E. Cane , C. Khosla , Biochemistry 2014, 53, 3796–3806. 24871074 10.1021/bi5004316 PMC4067149

[13] K. J. Weissman , C. J. Smith , U. Hanefeld , R. Aggarwal , M. Bycroft , J. Staunton , P. F. Leadlay , Angew. Chem. Int. Ed. 1998, 37, 1437–1440; 10.1002/(SICI)1521-3773(19980605)37:10<1437::AID-ANIE1437>3.0.CO;2-7 29710889

[14] C. J. Martin , M. C. Timoney , R. M. Sheridan , S. G. Kendrew , B. Wilkinson , J. C. Staunton , P. F. Leadlay , Org. Biomol. Chem. 2003, 1, 4144–4147. 14685317 10.1039/b310740j

[15] A. Rittner , K. S. Paithankar , K. V. Huu , M. Grininger , ACS Chem. Biol. 2018, 13, 723–732. 29328619 10.1021/acschembio.7b00718

[16] J. Molnos , R. Gardiner , G. E. Dale , R. Lange , Anal. Biochem. 2003, 319, 171–6. 12842120 10.1016/s0003-2697(03)00327-0

[17] C. D. Reeves , S. Murli , G. W. Ashley , M. Piagentini , C. R. Hutchinson , R. McDaniel , Biochemistry 2001, 40, 15464–15470. 11747421 10.1021/bi015864r

[18] F. Del Vecchio , H. Petkovic , S. G. Kendrew , L. Low , B. Wilkinson , R. Lill , J. Cortes , B. A. Rudd , J. Staunton , P. F. Leadlay , J. Ind. Microbiol. Biotechnol. 2003, 30, 489–494. 12811585 10.1007/s10295-003-0062-0

