# Supplementary material for: Understanding Substrate Selectivity of Phoslactomycin Polyketide Synthase by Using Reconstituted in Vitro Systems
Source: Chembiochem. 2020 Mar 30;21(14):2080–5. doi: 10.1002/cbic.202000112 (PMC7496768; doi:10.1002/cbic.202000112)
Supplement: Supplementary file 1 — Supplementary [file CBIC-21-2080-s001.pdf]

ChemBioChem

Supporting Information

Understanding Substrate Selectivity of Phoslactomycin Polyketide Synthase by Using Reconstituted in Vitro Systems

Kyra Geyer, Srividhya Sundaram, Peter Sušnik, Ulrich Koert, and Tobias J. Erb*© 2020 The Authors. Published by Wiley-VCH Verlag GmbH & Co. KGaA.
This is an open access article under the terms of the Creative Commons Attribution License, which permits use, distribution and reproduction in any medium, provided the original work is properly cited.

List of content

Supplementary Figures

Figure S1: SDS gel of enzymes 2

Figure S2: Full conversion of Pn_ACPs to *holo*-ACPs 3

Figure S3: Extracted ion chromatograms 4

Figure S4: Mass spectrometric data of pentaketide production 5

Figure S5: Mass spectrometric data of hexaketide production 6

Figure S6: Product spectrum of phoslactomycin *in vitro* assays 7

Figure S7: Michaelis Menten plots of KS-AT hydrolysis and transacylation 11

Figure S8: HPLC UV-vis traces of PnC_ACP loaded by PnC_KS-AT 12

Figure S9: HPLC UV-vis analysis for PnC_KS-AT transacylation rate 13

Figure S10: HPLC based transacylation assay to confirm results of SucC/SucD assay 14

Figure S11: Sequence alignment of AT domains from Pn PKS 15

Supplementary Schemes

Scheme S1: Steady state kinetic assay, relying on purified proteins 16

Supplementary Tables

Table S1: phosphopantetheinyl coverage, selfacylation and transacylation of Pn_ACPs 17

Table S2: List of polyketide masses (*m/z*) from the competition assays 18

Table S3: full table of app. K_M and app. k_{cat} values for hydrolysis and transacylation 19

Table S4: Calculated and detected masses of *holo*-, *apo*-, and acyl-ACP 20

Experimental section 21

Synthesis of (2*Z*)-cylcohexanepropenyl-SNAC 24

DNA Sequence information 29

References 39

Figure S1: SDS gel of enzymes used in this study. **A**) PnAV$_4$ (251.75 kDa), PnB (354.42 kDa), PnB-TE (374.95 kDa), PnC (176.81 kDa), PnC-TE (198.99 kDa) and PnD-TE (221.18 kDa) after affinity purification and size exclusion. **B**) Npt (25.78 kDa) after affinity purification, SucC (43.56 kDa) and SucD (31.94 kDa) after affinity purification and size exclusion. **C**) PnB_KS-AT1 (97.19 kDa), PnB_KS-AT2 (96.76 kDa), PnC_KS-AT (101.32 kDa), PnD_KS-AT (100.57 kDa) and **D**) PnB_ACP1 (12.77 kDa), PnB_ACP2 (12.77 kDa), PnC_ACP (12.01 kDa) and PnD ACP (13.84 kDa) after affinity purification and size exclusion.

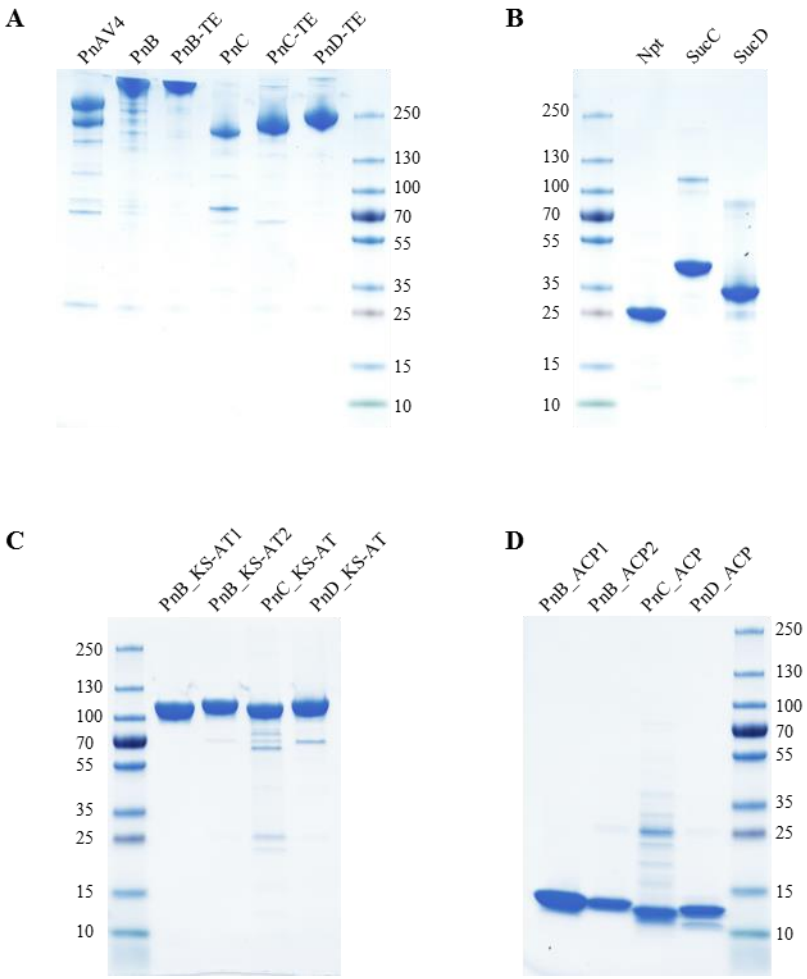

Figure S2: Full conversion of Pn_ACPs to *holo*-ACPs. Npt from *Streptomyces platensis* is able to fully convert *apo*-Pn_ACPs to *holo*-Pn_ACPs. **A)** PnB_ACP1, **B)** PnB_ACP2, **C)** PnC_ACP, **D)** PnD_ACP. For no reaction residual *apo*-Pn_ACPs could be detected. 100 µM ACP was incubated with 300 µM CoA, 1 µM Npt in 200 mM NaH_2PO_4 for 30 min at 28°C.

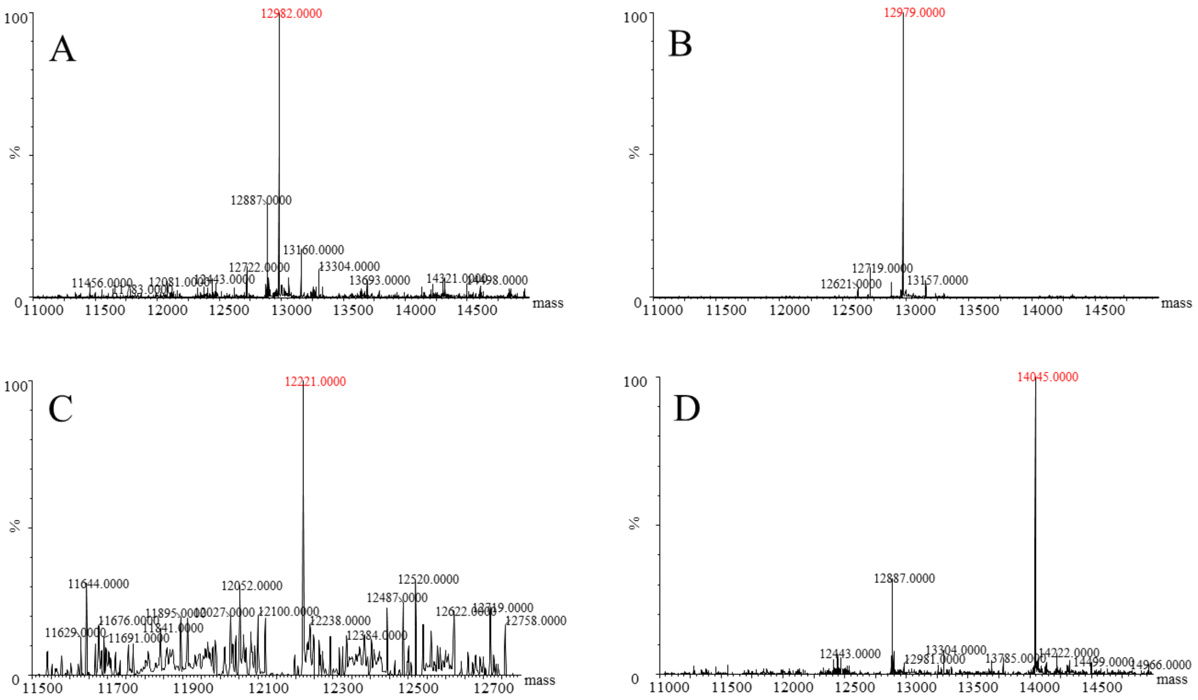

Figure S3: **Extracted ion chromatograms** from assays with PnA_{V4} and PnB (tetraketide system), PnA_{V4}, PnB, PnC-TE_{DEBS} (pentaketide system) and PnA_{V4}, PnB, PnC and PnD-TE_{DEBS} (hexaketide system). Products of the tetraketide and pentaketide system are three times labelled, upon incorporation of 2-C^{13} labelled malonyl-CoA, products of the hexaketide system four times. The tetraketide product (**4**) can only be found as in the dehydrated form (*m/z* $[M+H]^+$ 207.1380). Three masses are shown for the pentaketide product (*m/z* $[M+H]^+$ 277.1798, $[M+Na]^+$ 299.1618 and as *m/z* $[M+H]^+$ 259.1693) (**5b.2**, **5b.4**, **Figure S6**). For the hexaketide one mass corresponding to the oxidized, dehydrated (**6b.5**, **Figure S6**) ion is shown as an example (*m/z* $[M+H]^+$ 303.1955).

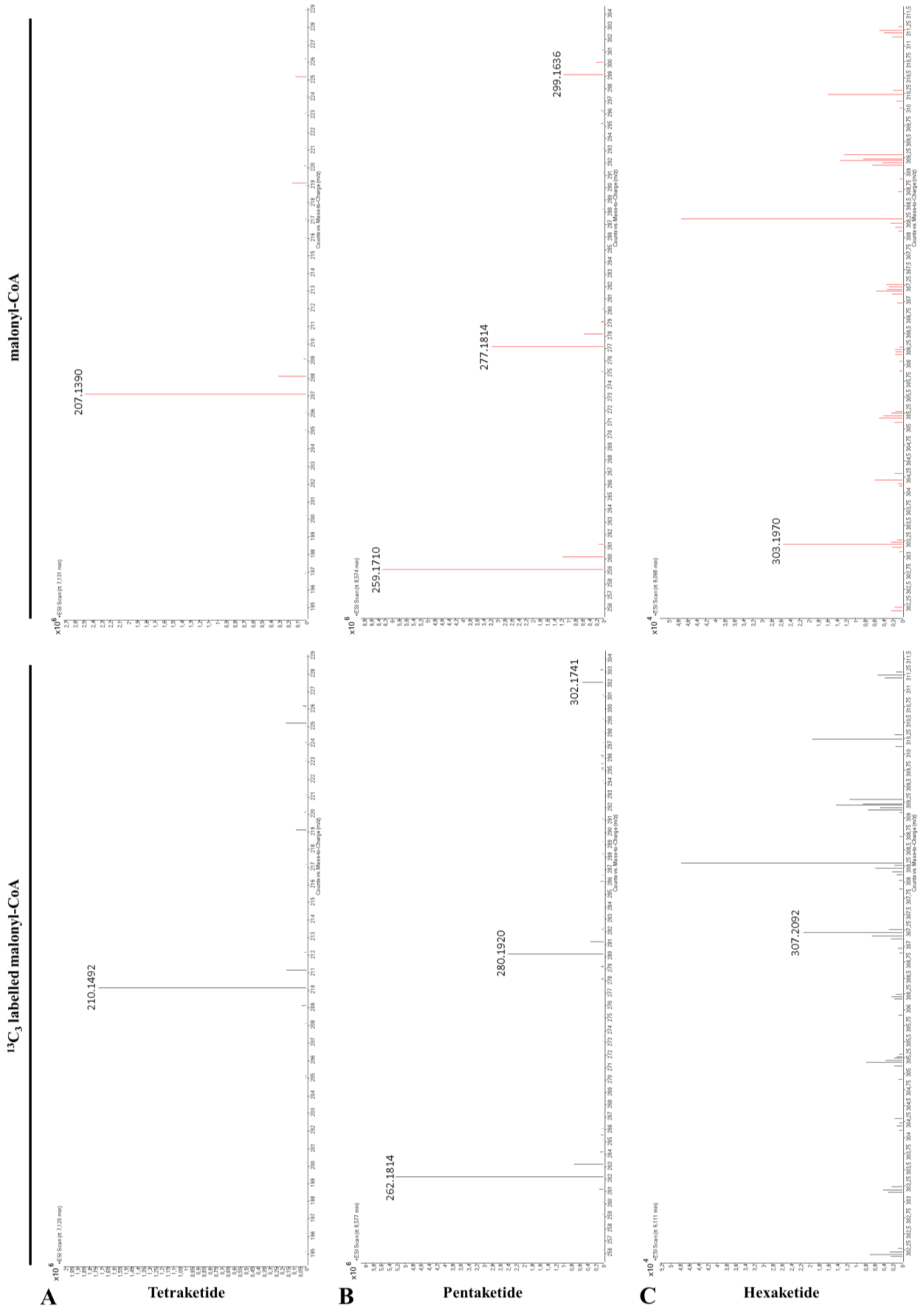

Figure S4: **Mass spectrometric data of pentaketide production. A)** Overlaid extracted ion chromatograms of **5a**, **5b**, **5c**, **5d** and **5e** from single competition assays. **B)** All assays were run with either malonyl-CoA (red) or 2-C^{13} malonyl-CoA (black). This resulted in double labeled pentaketide (PnB module 1 and module 2 incorporate malonyl-CoA). Searched for and shown are the masses of **5a-e.2** and **5f.4** (**Figure S6**). (**5a**: *m/z* 263.1645 and *m/z* 265.1702, **5b**: *m/z* 277.1798 and *m/z* 279.1859, **5c**: *m/z* 305.2111 and *m/z* 307.2174, **5d**: *m/z* 319.2268 and *m/z* 321.2331, **5e**: *m/z* 333.2424 and *m/z* 335.2487, **5f**: *m/z* 321.1849 and *m/z* 323.1914)

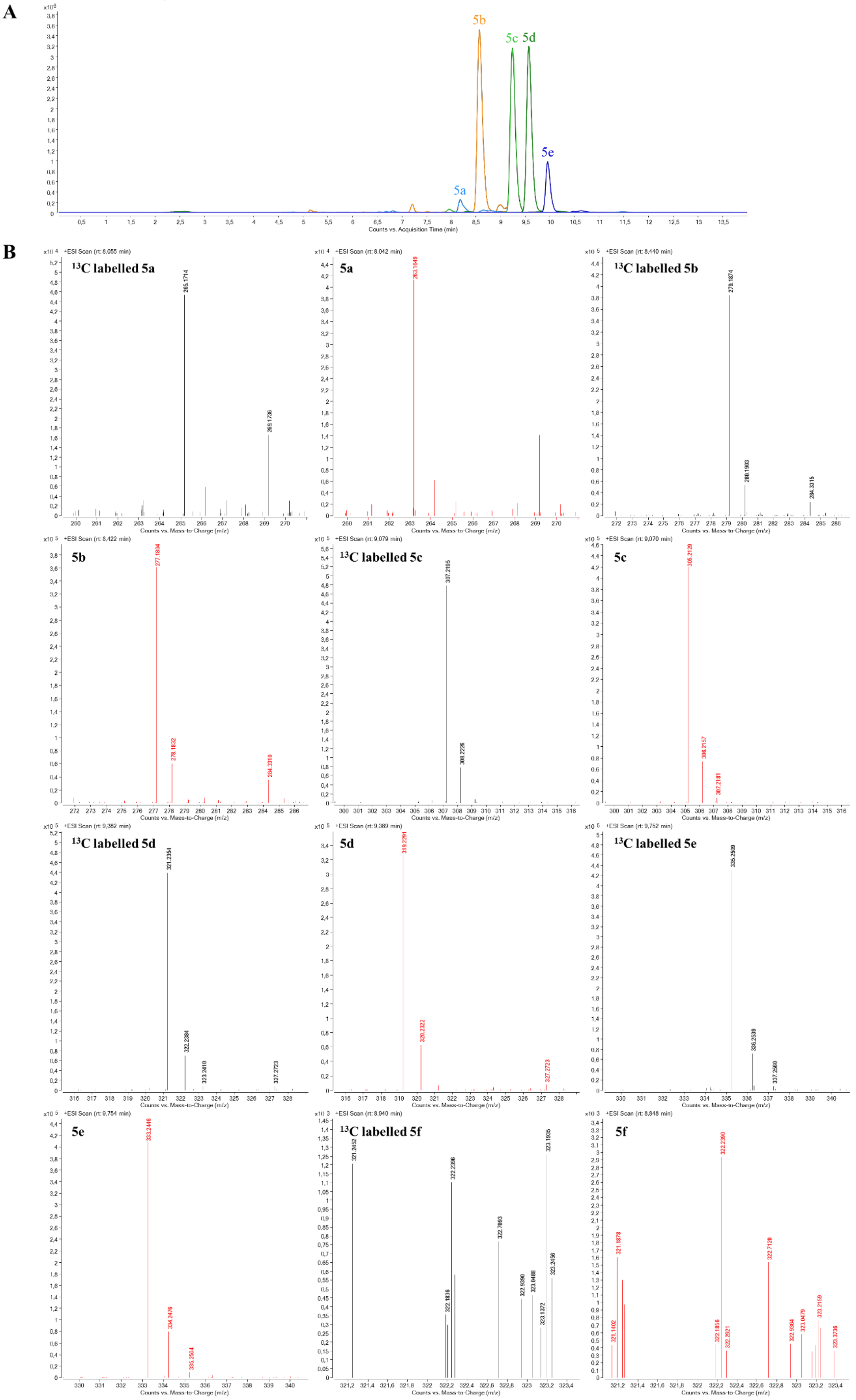

Figure S5: Mass spectrometric data of hexaketide production. **A)** Overlaid extracted ion chromatograms of **6a**, **6b**, **6c**, **6d** and **6e** from single extender unit assays. **B)** All assays were either run with malonyl-CoA (red) or 2-C[13] malonyl-CoA (black). This resulted in three time labeled hexaketide (PnB module1 and module 2 and PnD incorporate malonyl-CoA). Searched for and shown are the masses of **6a-e.6** (**Figure S6**). (**6a**: *m/z* 271.1693 and *m/z* 274.1784, **6b**: *m/z* 285.1849 and *m/z* 288.1941, **6c**: *m/z* 313.2162 and *m/z* 316.2255, **6d**: *m/z* 327.2319 and *m/z* 330.2414, **6e**: *m/z* 341.2475 and *m/z* 344.2569)

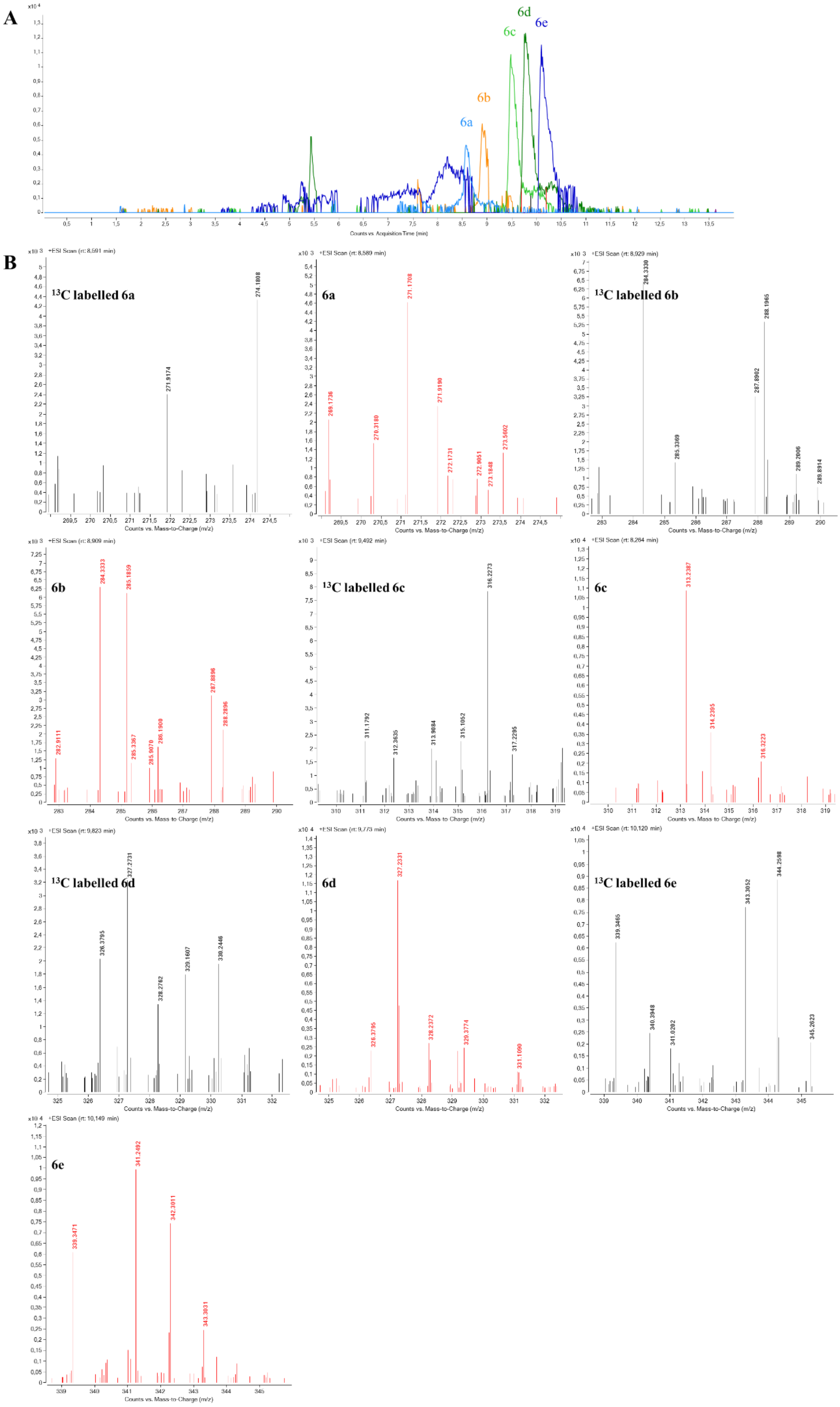

Figure S6: Product spectrum of phoslactomycin *in vitro* assays. Panel **A**, **B** and **E** show the proposed structures of the tetra-, penta- and hexaketide products of the reconstituted phoslactomycin *in vitro* polyketide system. Panel **C**, **D**, **F** and **G** show the products that are expected when respective KR are catalytically knocked out. Marked in blue, by the formula and retention time (RT), are the masses that were detected in the mass spectrometric measurements, as well as their derived structures. The structures were derived from the proposed biosynthetic pathway for the phoslactomycin polyketide backbone[1].

Tetraketides

A) After synthesis the tetraketide **4** is bound to the ACP of PnB_ACP2, which can be released as an acid (**4**). Tetraketide **4** can only be found as the dehydrated product **4.1**. Strong conjugation by the double bonds destabilize the hydroxyl group at C-3 and **4.1** is formed. Alternatively, DEBS_TE forms a four membered lactone ring, although such a function was never reported for DEBS_TE.

Pentaketides

B) The linear pentaketide **5.b** is bound to PnC_ACP after synthesis. However, **5.b** cannot be detected by mass spectrometric analysis, pointing towards lactonization by DEBS_TE and/or spontaneous lactonization, resulting in **5b.1**. Electron spray ionization (ESI) during mass spectrometric measurements causes dehydration, forming **5b.3**, with the same RT as **5b.1**. The hydroxyl group at C-3 of **5b.1** is prone to oxidation in solution, leading to the expected formation of **5b.2**. Further dehydration by ESI leads to formation of **5b.4**, with the same RT as **5b.2**. An alternative route for the synthesis of **5b.2** would be a (partially) inactive PnC_KR. However, since **5b.1** correspond to the reduced pentaketide, it seems rather likely that PnC_KR is fully functional and that **5b.2** is generated by subsequent oxidation of the fully reduced pentaketide.

C) In the catalytic knock out of the KR of PnB module 2, PnB^{Y3074F}, the C-5 of the pentaketide should carry a ketone group instead of a hydroxyl-group, **5b.5** is bound to PnC_ACP. Keto groups are prone to enolization, enabling the formation of product **5b.6**, either by releasing itself by ring formation from the thioester linkage on the ACP or catalyzed by DEBS_TE. Due to the loss of water by mass spectrometric analysis **5b.4** can also be measured in reaction with PnB^{Y3074F}.

D) Product **5b.7**, the product of assays with PnC_KR catalytic knockout PnC^{Y1367F}, is bound to PnC_ACP and is the expected product if the free acid is released by DEBS_TE. **5b.7** can be detected in mass spectrometric analysis. This product can form the lactone **5b.2** (as discussed in panel B) by spontaneous lactone ring formation, or catalyzed by DEBS_TE on PnC_ACP. Formation of **5b.4** can be explained by ESI-induced dehydration.

Hexaketides

E) The linear hexaketide **6b** is bound to PnD_ACP, the acid **6b** and product **6b.1** cannot be distinguished by mass, therefore generation of the double bond by PnD_DH cannot be verified. Product **6b.1** could be a result of spontaneous and/or DEBS_TE catalyzed lactone ring formation prior to dehydration by PnD_DH. The following product **6b.4** could result from **6b.1** by oxidation in solution. An alternative for the formation of **6b.4** is the release from the thioester on PnD_ACP by ring formation of the unreduced hexaketide **6b.7**, catalyzed by DEBS_TE or spontaneous ring formation. The product **6b.4** can both dehydrate in water (indicated by shift in RT) and by mass spectrometric measurement (same RT), forming **6b.5**. Formation of **6b.6** can be explained by ESI-induced dehydration.

F) Linear hexaketide **6b.8** with a keto group at C-7 as the product of assays containing PnB^{Y3074F} is bound to PnD_ACP but cannot be found as an acid in solution, however a mass corresponding to **6b.9** can be detected. **6b.9** corresponds to a hexaketide where PnD_DH did not dehydrate the hydroxyl group at C-3, probably due to premature release by DEBS_TE. Following products **6b.10**, **6b.11** and **6c.12** corresponding to lactone ring formation, oxidation or dehydration by ESI (reactions that were observed with the pentaketide) cannot be found. However masses corresponding to product **6b.5** can be found additionally to **6b.9**.

G) Product **6b.13** as the expected product of assays containing PnC^{Y1367F} would be bound to PnD_ACP but cannot be found by mass spectrometric analysis. Like observed before, the hexaketide could be released by pyrone ring formation of the thioester with the enolized ketogroup at C-5, prior to dehydration by PnD_DH. Subsequently water can be lost to form **6b.15** By ESI measurement another water is lost and **6b.6** is formed (same RT as **5b.15**).

Conclusion

Altogether, our analysis demonstrated that the Pn PKS enzymes are functional *in vitro* and can be combined stepwise to produce tetra- penta- and hexaketide Pn polyketide derivatives. In all cases, we detected dehydrated and oxidized derivatives of the expected polyketides as the main products and are able to propose structures to the calculated molecular formula. To improve formation of the linear hexaketide, we created a catalytic knockout of the PnC_KR (PnC^{Y1367F}). Combining PnC^{Y1367F} with PnD TE_{DEBS} did not improve linear hexaketide formation but led to the formation of **6b.15** (similar spontaneous ring formation is also observed in other PKS [2]). In hexaketide assays, we observed premature release of pentaketide, pointing to autocatalytic lactonization. Lactonization and self-release has also been observed for the DEBS system [3]. To circumvent autocatalytic release and increase hexaketide formation, we decided to remove the hydroxyl group attacking the thioester that is installed by the KR in PnB module 2 (PnB^{Y3074F}). This resulted in production of **6b.9**, albeit total hexaketide production was reduced (18 ± 0.5% residual production). Similar deleterious effects of KR mutants have been observed in other PKS systems [4]. These experiments allowed us to establish and characterize the behavior of the Pn system for subsequent studies on extender unit selectivity.

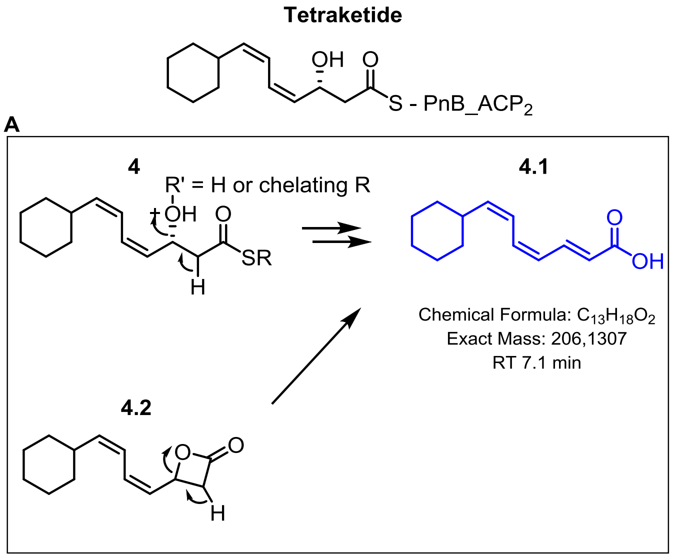
Tetraketide
OH
O
S - PnB_ACP2
A
4
R' = H or chelating R
OH
O
SR
H
4.1
O
OH
Chemical Formula: C13H18O2
Exact Mass: 206,1307
RT 7.1 min
4.2
O
O
H

Pentaketide

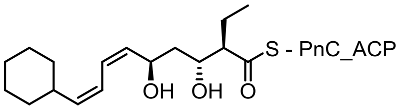

B

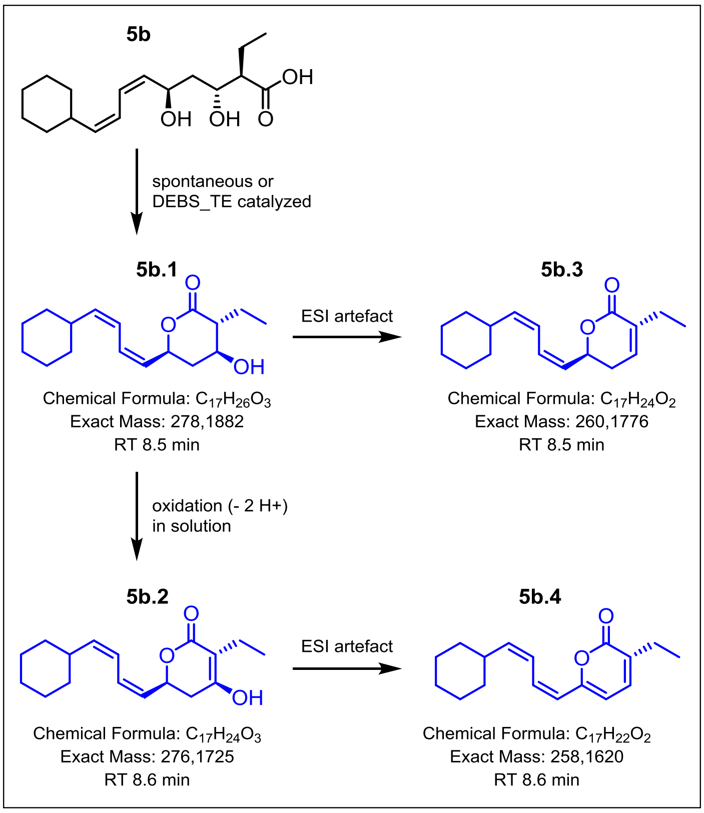

C **Pentaketide PnB^{Y3074F}**

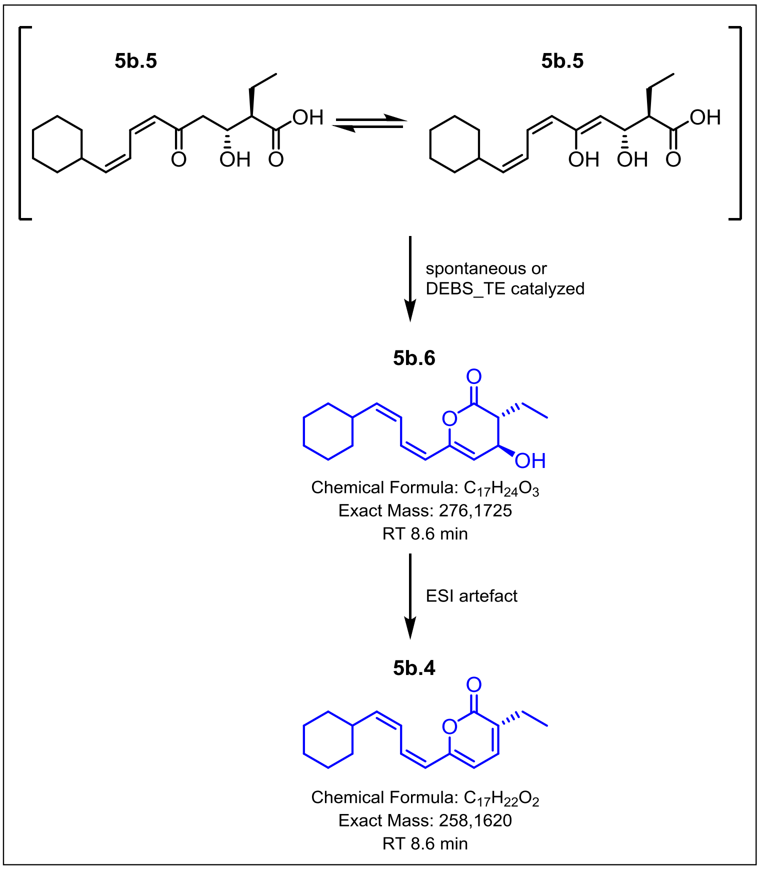

D **Pentaketide PnC^{Y1367F}**

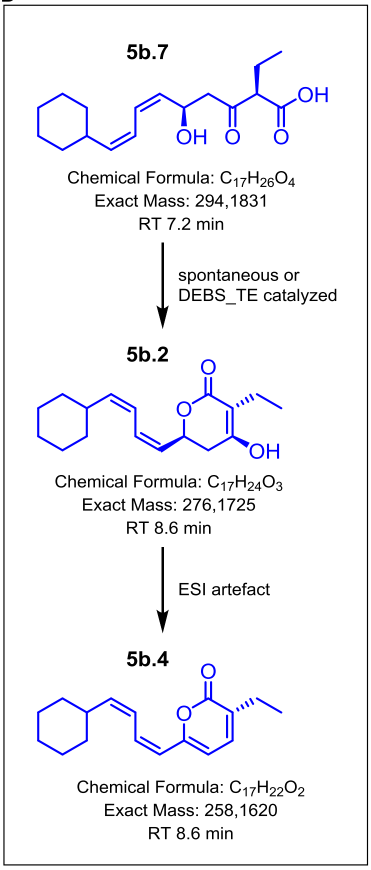

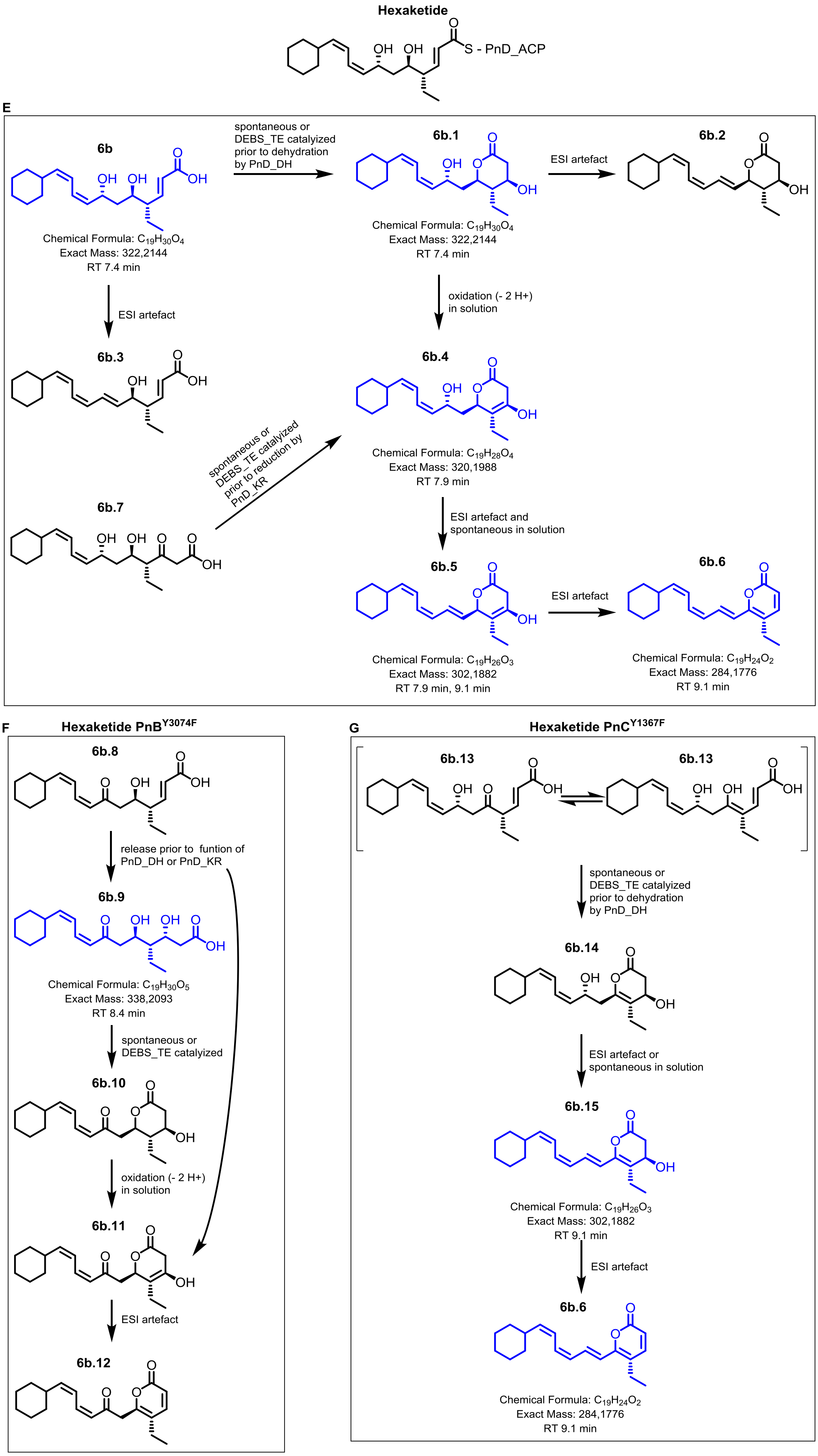
Hexaketide
S - PnD_ACP
E
6b
Chemical Formula: C19H30O4
Exact Mass: 322,2144
RT 7.4 min
spontaneous or DEBS_TE catalyzed prior to dehydration by PnD_DH
6b.1
Chemical Formula: C19H30O4
Exact Mass: 322,2144
RT 7.4 min
ESI artefact
6b.2
ESI artefact
6b.3
oxidation (- 2 H+) in solution
6b.4
Chemical Formula: C19H28O4
Exact Mass: 320,1988
RT 7.9 min
spontaneous or DEBS_TE catalyized prior to reduction by PnD_KR
6b.7
ESI artefact and spontaneous in solution
6b.5
Chemical Formula: C19H26O3
Exact Mass: 302,1882
RT 7.9 min, 9.1 min
ESI artefact
6b.6
Chemical Formula: C19H24O2
Exact Mass: 284,1776
RT 9.1 min
F
Hexaketide PnB^Y3074F
6b.8
release prior to funtion of PnD_DH or PnD_KR
6b.9
Chemical Formula: C19H30O5
Exact Mass: 338,2093
RT 8.4 min
spontaneous or DEBS_TE catalyzed
6b.10
oxidation (- 2 H+) in solution
6b.11
ESI artefact
6b.12
G
Hexaketide PnC^Y1367F
6b.13
6b.13
spontaneous or DEBS_TE catalyzed prior to dehydration by PnD_DH
6b.14
ESI artefact or spontaneous in solution
6b.15
Chemical Formula: C19H26O3
Exact Mass: 302,1882
RT 9.1 min
ESI artefact
6b.6
Chemical Formula: C19H24O2
Exact Mass: 284,1776
RT 9.1 min

Figure S7: Michaelis Menten plots of KS-AT hydrolysis and transacylation measured with the SucC/SucD based steady state kinetic assay. Each reaction was measured in three technical replicates. **A**) PnB_KS-AT1, **B**) PnB_KS-AT2, **C**), PnC_KS-AT (substrates used are malonyl- (**2**), methylmalonyl- (**3a**), ethylmalonyl- (**3b**), butylmalonyl-CoA (**3c**) 3-methylbutylmalonyl- (**3c**), hexylmalonyl-CoA (**3e)**), **D**) PnD_KS-AT

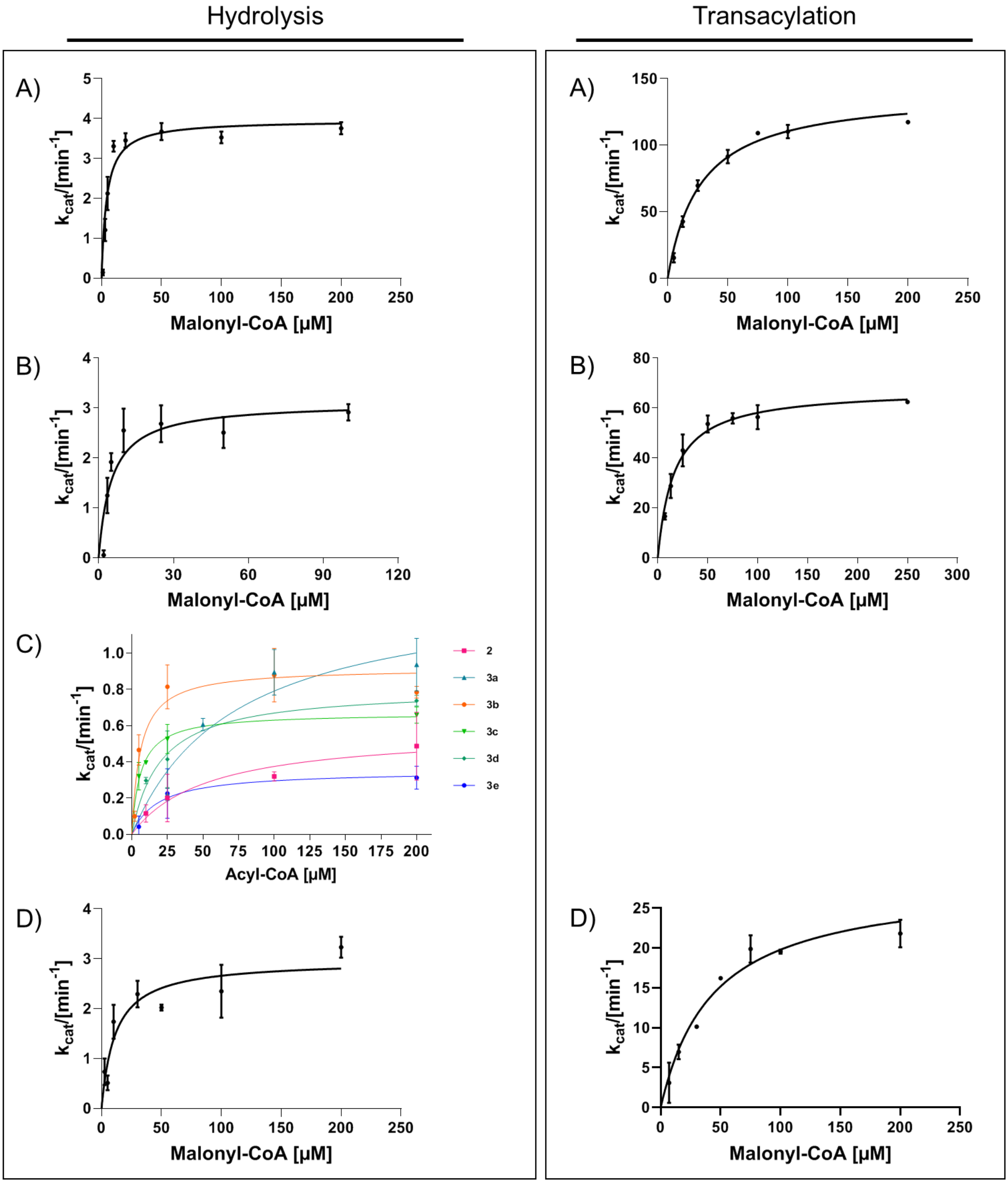

Figure S8: HPLC UV-vis traces of PnC_ACP loaded by PnC_KS-AT (indicated with arrows) with the tested extender units. Incubation time of 5 µM PnC_KS-AT, 100 µM PnC_ACP and 300 µM CoA substrate was 2h, except for ethylmalonyl-CoA (5 min incubation time) Reaction was run at 25 °C. A shift of retention time can be observed for all substrates, except methylmalonyl-PnC_ACP, which elutes at the same retention time as *holo*-PnC_ACP. Substrates used were malonyl- (**2**), methylmalonyl- (**3a**), ethylmalonyl- (**3b**), butylmalonyl-CoA (**3c**) 3-methylbutylmalonyl- (**3c**), hexylmalonyl- (**3e**) and benzylmalonyl-CoA (**3f**).

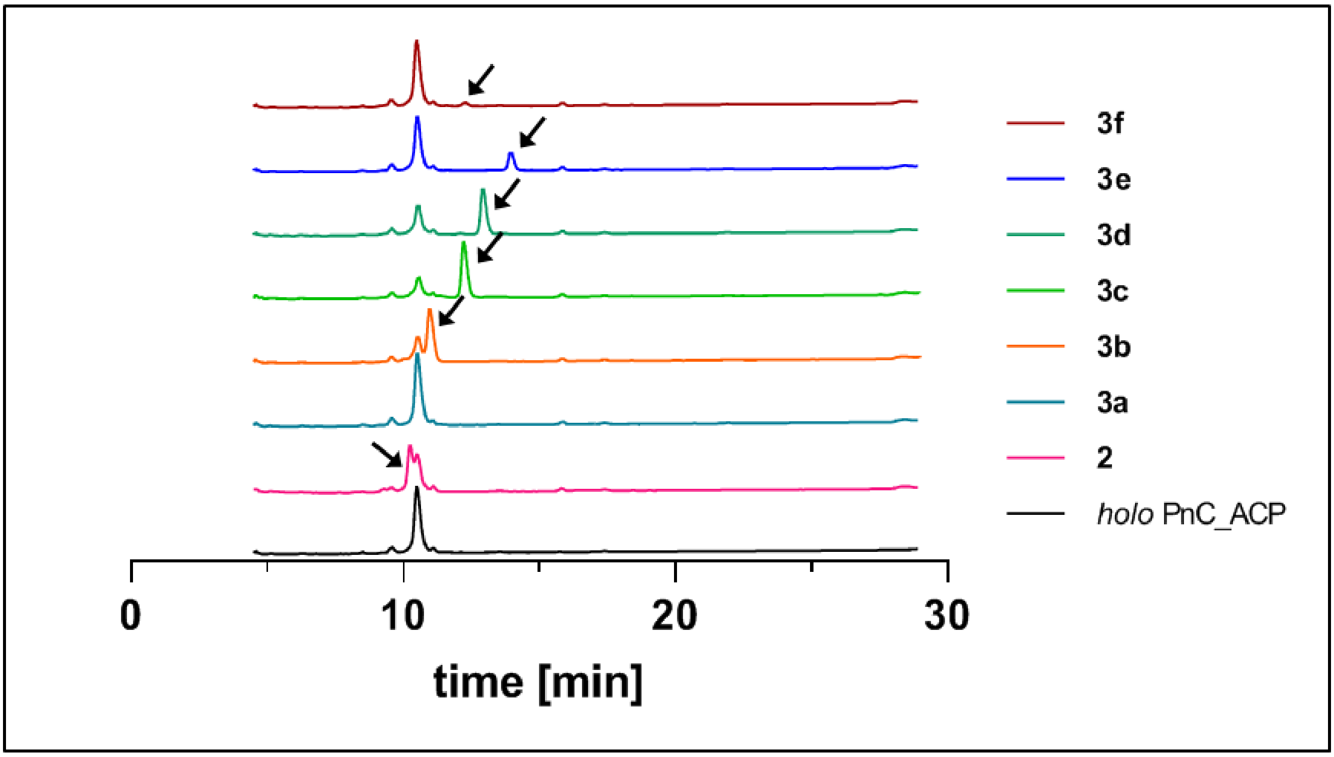

Figure S9: **HPLC UV-vis analysis for PnC_KS-AT transacylation rate**. The three colors represent three technical replicates. Slopes from the linear increase were used for the calculation of k_{cat}. Relative amount of acyl-ACP is given on the y-axis. Substrates used are **A**) ethylmalonyl-CoA, **B**) malonyl-CoA, **C**) butylmalonyl-CoA, **D**) 3-methylbutylmalonyl-CoA, **E**) hexylmalonyl-CoA. For ethylmalonyl-ACP and butylmalonyl-ACP a decrease of acyl-ACP can be observed after 25 min and after 150 min, respectively. Note also the maximum conversion reached in the transacylation assay varies between approx. 70% for ethylmalonyl-, butylmalonyl-, 3-methylbutylmalonyl-ACP and approx. 40% for malonyl-ACP. Transacylation rate could not be determined for methylmalonyl-CoA, due to same retention time of *holo*-PnC_ACP and methylmalonyl-PnC_ACP (see also **Figure S7**).

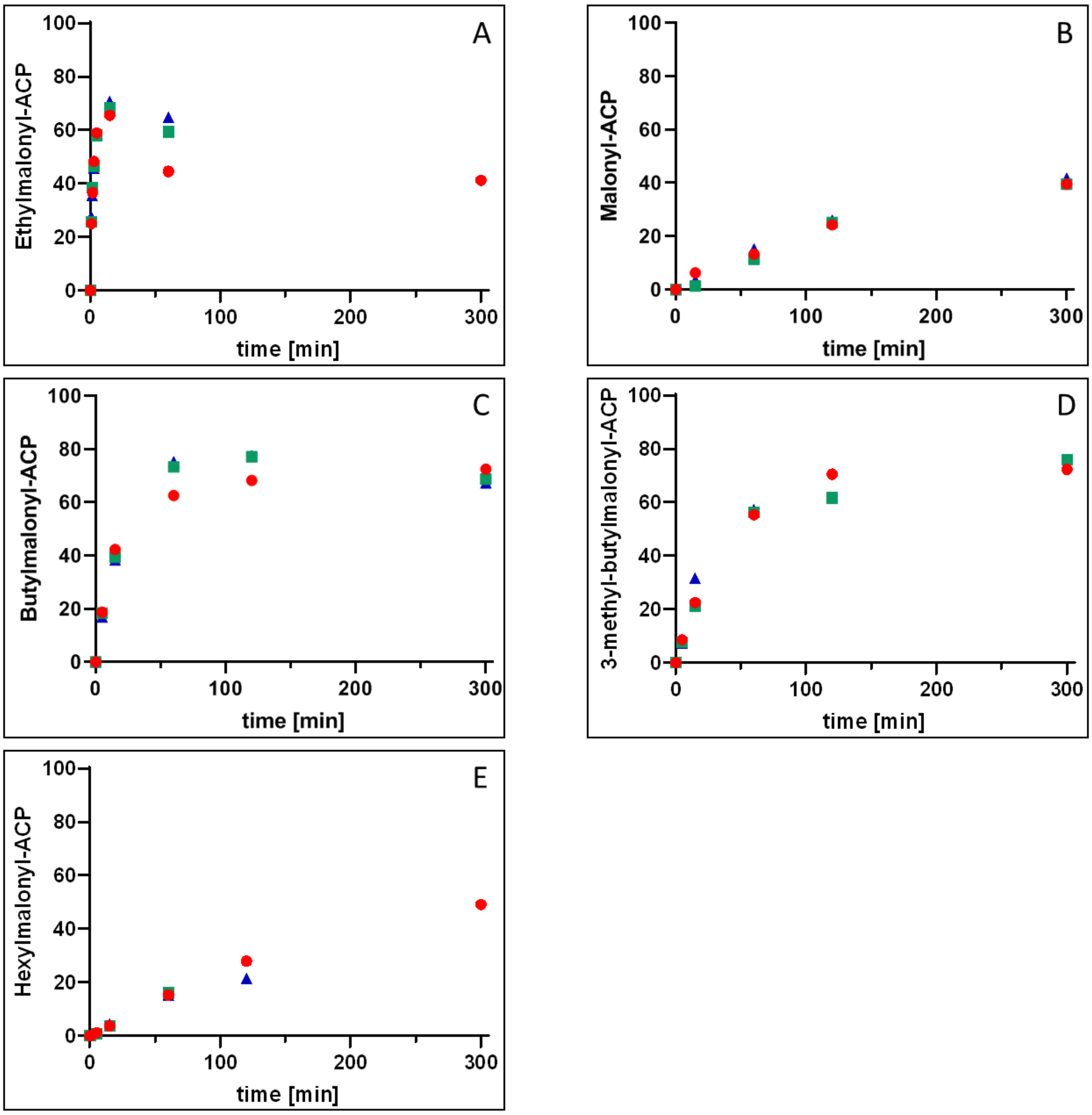

Figure S10: HPLC based transacylation assay to confirm results of SucC/SucD assay. For the measurement of the k_{cat} of transacylation by PnC_KS-AT the SucC/SucD based assay could not be used due to high background activity, which could also not be reduced by repeated size exclusion chromatography of the used proteins. To assure that the results from the SucC/SucD based assay and the HPLC assay are comparable the control described here was done. Turnover of **A)** PnB_KS-AT2 (substrate malonyl-CoA) and **B)** PnC_KS-AT (substrates used were ethylmalonyl- and butylmalonyl-CoA). 500 nM KS-AT and 400 µM cognate ACP were mixed with 600 µM substrate in 200 mM NaH_2PO_4, pH 7.2 (same protein concentrations in SucC/SucD based assay). The reaction was stopped by adding formic acid and samples were analyzed using HPLC. With the linear slope the turnover was calculated. The k_{cat} of PnB_KS-AT2 with malonyl-CoA was 49.3 per minute and for PnC_KS-AT with ethylmalonyl-CoA 3.4 per minute, with butylmalonyl-CoA 0.31 per minute. With the SucC/SucD based assay a k_{cat} of 74 (PnB_KS-AT2), 4 (PnC_KS-AT with ethylmalonyl-CoA) and 0.6 (PnC_KS-AT with butylmalonyl-CoA) was measured. The determined turnover rates are similar which assures that the measured kinetics displayed in **Table S3** and **Table 1** can be compared.

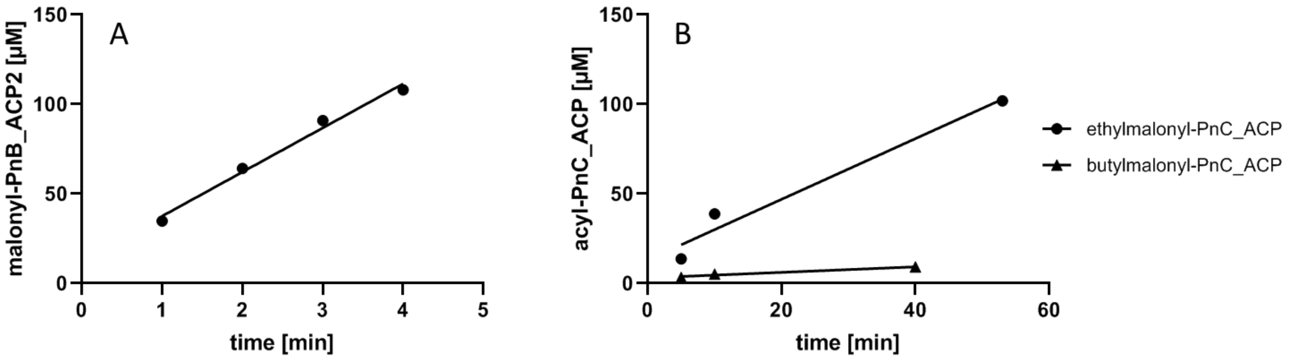

Figure S11: Sequence alignment of AT domains from Pn PKS. Indicated are three extender unit specificity conferring motifs. The canonical GHS can be found in all malonyl-CoA specific Pn_AT domains (amino acid position 92-96), while PnC shows a GSS. Malonyl-CoA specific AT domains contain the highly conserved HAFH motif, methylmalonyl-CoA specific domains most commonly use the YASH motif. Ethylmalonyl-CoA specific AT domains have a less well conserved motif at this position, generally XAGH, with X being F, T, V or H [5]. A motif close to this, IAGH is found in the loading AT domain from PnA. PnC shows the motif CASH in the binding pocket, PnE shows IAAH (amino acid position 198-201).

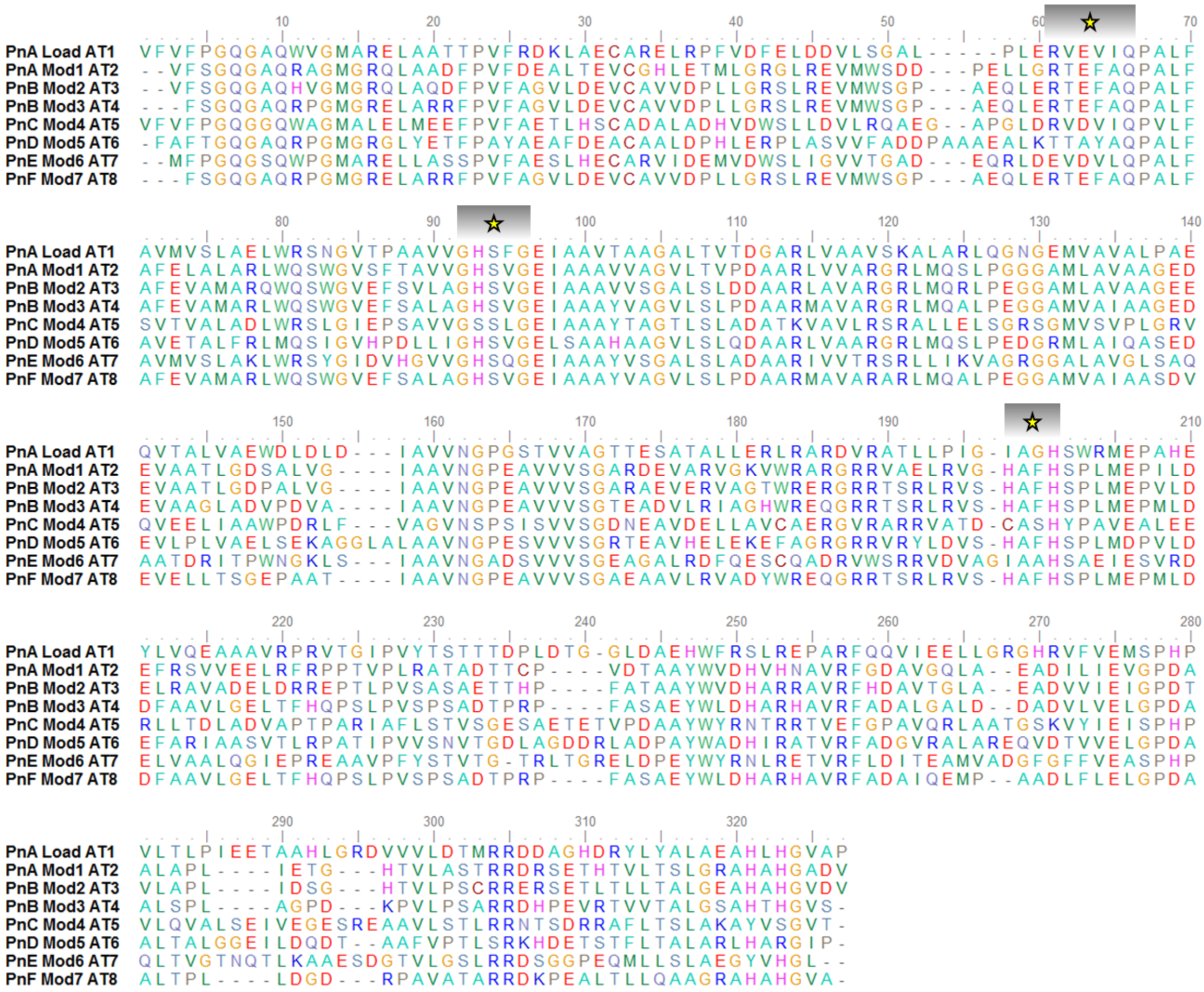

Scheme S1: Steady state kinetic assay, relying on purified proteins. KS-AT didomains and standalone ACPs are used. Upon covalent binding of the acyl-residue to the AT, CoA-SH is released. With no ACP acceptor available, the acyl-residue is subsequently hydrolyzed by nucleophilic attack of water and the AT can bind a new acyl-residue. If *holo*-ACP is available, the acyl-residue is transferred and the AT can bind a new acyl-residue. Free CoA-SH is converted to succinyl-CoA by *E. coli* SucC/SucD. In this reaction ADP is produced, which can be used by pyruvate kinase/lactic dehydrogenase (PK/LDH) under NADH consumption. For Michaelis Menten plots with data collected with this assay refer to **Figure S6.**

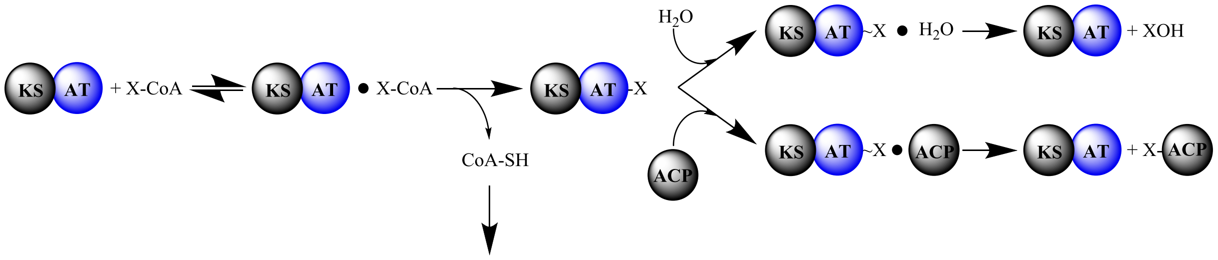

$$\text{succinate} + \text{ATP} + \text{CoA-SH} \xrightleftharpoons{\text{SucC/SucD}} \text{succinyl-CoA} + \text{ADP}$$

$$\text{phosphoenolpyruvate} + \text{ADP} \xrightleftharpoons{\text{PK}} \text{pyruvate} + \text{ATP}$$

$$\text{pyruvate} + \text{NADH} \xrightleftharpoons{\text{LDH}} \text{lactate} + \text{NAD}^+$$

Table S1: phosphopantetheinyl coverage, selfacylation and transacylation of Pn_ACPs. Extent of phosphopantetheinlyation of Pn_ACP purified from *E. coli* BAP1. Acyl-ACP buildup upon 2 h incubation of 100 µM *holo*-ACP with 300 µM extender unit at 25°C ("selfacylation") and acyl-ACP buildup upon 2 h incubation of 5 µM KS-AT, 300 µM CoA thioester, 100 µM ACP at 25°C. The percentage of *apo*-, *holo*- and acyl-ACP was determined by mass spectrometric measurements. Substrates used were malonyl- (**2**), methylmalonyl- (**3a**), ethylmalonyl- (**3b**), butylmalonyl-CoA (**3c**) 3-methylbutylmalonyl- (**3c**), hexylmalonyl- (**3e**) and benzylmalonyl-CoA (**3f**).
Malonyl-, methylmalonyl- and 3-methylmalonyl-ACP can be detected without the presence of an AT domain. This is likely due to transesterification of the acyl-residue from CoA to the thiolgroup of the ACP. In case of malonyl- and methylmalonyl-CoA this is also partly due to sfp mediated loading of the ACP during expression in *E. coli* BAP1. Subtracting already acylated ACPs ("selfacylation") from the transacylated ACP produced by the AT-mediated transacylation shows that only malonyl-CoA **2** is transferred in the case of PnB_AT1, PnB_AT2 and PnD_AT and that all substrates (BenzM-ACP under detection limit) are transferred in the case of PnC_AT.

	PnB_ACP1	PnB_ACP2	PnC_ACP	PnD_ACP
***holo*-ACP**	26%	75%	96%	66%
**Selfacylation of ACP (%)**				
**M-ACP (2)**	0	6.4	8.3	0
**MM-ACP (3a)**	30	5.7	0	12.5
**EM-ACP (3b)**	0	0	0	0
**BM-ACP (3c)**	0	0	0	0
**3MBM-ACP (3d)**	9.1	0	0	3.5
**HM-ACP (3e)**	0	0	0	0
**BenzM-ACP (3f)**	0	0	0	0
**Transacylation of ACP (%)**				
**M-ACP (2)**	100	63.8	55.9	19.1
**MM-ACP (3a)**	35.8	9.3	78.8	6.6
**EM-ACP (3b)**	0	0	62	0
**BM-ACP (3c)**	0	0	68.8	0
**3MBM-ACP (3d)**	9.5	0	60.4	3
**HM-ACP (3e)**	0	0	16.3	0
**BenzM-ACP (3f)**	0	0	0	0

Table S2: List of polyketide masses (*m/z*) from the competition assays. Both, *m/z* calculated and detected are given together with the retention time. **4** corresponds to the tetraketide, **5** to the pentaketide and **6** to the hexaketide, **b** indicates ethyl-, **a** methyl-, **c** butyl-, **d** 3-methyl-butyl-, **e** hexyl- and **f** benzyl-residue.

Compound	Retention time [min]	[H+] m/z calculated	[H+] m/z detected	Δ [m/z]	[Na+] m/z calculated	[Na+] m/z detected	Δ [m/z]
**4**	7.1	207.1380	207.1390	0.0010	229.1199	-	
**5b**	8.5	279.1955	-		301.1774	301.1757	0.0017
	8.5	261.1849	261.1852	0.0003	283.1669	-	
	8.6	277.1798	277.1802	0.0004	299.1618	299.1624	0.0006
	8.6	259.1693	259.1694	0.0001	281.1512	-	
**5a**	8.1	265.1798	265.1799	0.0001	287.1618	287.1599	0.0019
	8.1	247.1693	247.1697	0.0004	269.1512	-	
	8.2	263.1642	263.1648	0.0006	285.1461	285.1468	0.0007
	8.2	245.1536	245.1543	0.0007	267.1355	-	
**5c**	-	307.2268	-		329.2087	-	
	-	289.0313	-		311.1982	-	
	9.2	305.2111	305.2103	0.0008	327.1931	327.1920	0.0011
	9.2	287.2006	287.2001	0.0005	309.1825	-	
**5d**	-	321.2424	-		343.2243	-	
	-	303.2318	-		325.2138	-	
	9.6	319.2264	319.2269	0.0005	341.2087	341.2079	0.0008
	9.6	301.2162	301.2168	0.0006	323.1981	-	
**5e**	-	335.2581	-		357.2400	-	
	-	317.2475	-		339.2295	-	
	9.9	333.2424	333.2416	0.0008	355.2244	355.2236	0.0008
	9.9	315.2319	315.2312	0.0007	337.2138	-	
**5f**	-	341.2111	-		363.1930	-	
	-	323.2005	-		345.1825	-	
	9.0	339.1955	339.1972	0.0017	361.1774	361.1772	0.0002
	9.0	321.1849	321.1857	0.0008	343.1668	-	
**6b**	7.4	323.2217	-		345.2036	345.2046	0.0010
	7.9	321.2060	-		343.1880	343.1872	0.0008
	7.9; 9.1	303.1955	303.1953	0.0002	325.1774	325.1775	0.0001
	9.1	285.1849	285.1848	0.0001	307.1669	-	
**6a**	7.1	309.2060	-		331.1880	331.1919	0.0039
	7.6	307.1904	307.1923	0.0019	329.1730	-	
	7.6; 8.7	289.1798	289.1819	0.0021	311.1618	311.1647	0.0029
	8.7	271.1693	271.1718	0.0025	293.1512	-	
**6c**	-	351.2530	-		373.2349	-	
	8.4	349.2373	-		371.2193	371.2222	0.0029
	9.7	331.2268	331.2220	0.0048	353.2087	353.2109	0.0022
	9.7	313.2162	313.2176	0.0014	335.1982	-	
**6d**	-	365.2686	-		387.2505		
	8.9	363.2529	-		385.2349	385.2378	0.0029
	10.0	345.2424	345.2441	0.0017	367.2243	367.2281	0.0038
	10.0	327.2319	327.2342	0.0023	349.2138	-	
**6e**	-	379.2843	-		401.2662	-	
	9.3	377.2686	-		399.2506	-	
	10.1	359.2581	359.2613	0.0032	381.2400	381.2440	0.0040
	10.1	341.2475	341.2530	0.0055	363.2295	-	

Table S3: full table of app. K_M and app. k_{cat} values for hydrolysis and transacylation by PnB_KS-AT1, PnB_KS-AT2, PnC_KS-AT and PnD_KS-AT.

	**Hydrolysis**											
	PnB_KS-AT1			PnB_KS-AT2			PnC_KS-AT			PnD_KS-AT		
**Substrate**	K_M [µM]	k_{cat} [min^{-1}]	k_{cat}/K_M	K_M [µM]	k_{cat} [min^{-1}]	k_{cat}/K_M	K_M [µM]	k_{cat} [min^{-1}]	k_{cat}/K_M	K_M [µM]	k_{cat} [min^{-1}]	k_{cat}/K_M
**2**	4.3±0.8	4±0.15	0.9	5.2±0.8	3±0.14	0.6	53±41	0.6±0.15	0.01	11.5±3.3	3±0.2	0.26
**3a**	-[a]	-	-	-	-	-	73±33	1.4±0.3	0.02	-	-	-
**3b**	-	-	-	-	-	-	6±1.8	0.9±0.06	0.15	-	-	-
**3c**	-	-	-	-	-	-	6.2±1.4	0.7±0.04	0.11	-	-	-
**3d**	-	-	-	-	-	-	20±6.7	0.8±0.08	0.04	-	-	-
**3e**	-	-	-	-	-	-	19±15.5	0.4±0.08	0.02	-	-	-
**3f**	-	-	-	-	-	-	-	-	-	-	-	-
	**Transacylation**											
	PnB_KS-AT1			PnB_KS-AT2			PnC_KS-AT			PnD_KS-AT		
**Substrate**	K_M [µM]	k_{cat} [min^{-1}]	k_{cat}/K_M	K_M [µM]	k_{cat} [min^{-1}]	k_{cat}/K_M	K_M [µM]	k_{cat} [min^{-1}]	k_{cat}/K_M	K_M [µM]	k_{cat} [min^{-1}]	k_{cat}/K_M
**2**	27±2.6	140±4.3	5.2	16±1.7	74±2	4.6	n.d[b]	0.04±0.002	-	44±7.2	28±1.8	0.64
**3a**	-	-	-	-	-	-	n.d	n.d	-	-	-	-
**3b**	-	-	-	-	-	-	n.d	4±0.1	-	-	-	-
**3c**	-	-	-	-	-	-	n.d	0.6±0.03	-	-	-	-
**3d**	-	-	-	-	-	-	n.d	0.3±0.07	-	-	-	-
**3e**	-	-	-	-	-	-	n.d	0.05±0.001	-	-	-	-
**3f**	-	-	-	-	-	-	-	-	-	-	-	-

[a] not detected. **[b]** not determined

Table S4: Calculated and detected masses of *holo*-, *apo*-, and acyl-ACP. Given are the masses for PnB_ACP1, PnB_ACP2, PnC_ACP and PnD_ACP for the substrates malonyl-CoA **2** (M-ACP), methylmalonyl-CoA **3a** (MM-ACP), ethylmalonyl-CoA **3b** (EM-ACP), butylmalonyl-CoA **3c** (BM-ACP), 3-methylbutylmalonyl-CoA **3d** (3MBM-ACP), hexylmalonyl-CoA **3e** (HM-ACP) and benzylmalonyl-CoA **3f** (BenzM-ACP)

		PnB_ACP1	PnB_ACP2	PnC_ACP	PnD_ACP
***holo*-ACP**	**calculated**	12983.32	12980.41	12222.47	14046.58
	**found**	12982.01	12979.0	12221.00	14045.00
***apo*-ACP**	**calculated**	12643.23	12640.32	11882.38	13706.50
	**found**	12642.00	12639.00	11863.00	13705.00
**M-ACP (2)**	**calculated**	13069.32	13066.41	12308.47	14132.58
	**found**	13068.00	13065.00	12307.00	14131.00
**MM-ACP (3a)**	**calculated**	13083.33	13080.43	12322.48	14146.60
	**found**	13082.00	13079.00	12321.00	14146.00
**EM-ACP (3b)**	**calculated**	13097.35	13094.44	12336.50	14160.61
	**found**	-	-	12335.00	-
**BM-ACP (3c)**	**calculated**	13125.38	13122.47	12364.53	14188.64
	**found**	-	-	12364.00	-
**3MBM-ACP (3d)**	**calculated**	13139.40	13136.49	12378.55	14202.66
	**found**	13139.00	-	12378.00	-
**HM-ACP (3e)**	**calculated**	13153.41	13150.50	12392.56	14216.67
	**found**	-	-	12391.00	-
**BenzM-ACP (3f)**	**calculated**	13159.37	13156.46	12398.52	14222.63
	**found**	-	-	12399.00	-

Experimental section

Phoslactomycin PKS *in vitro* assays. The production of phoslactomycin polyketide derivatives was initiated using PnA_{V4} and CHC-CoA (**1**), or PnB and (2*Z*)-cyclohexanepropenyl-SNAC (**7**). The assay was run at a volume of 50 µL and contained the PKS proteins at 9 µM concentration, Npt at 3.5 µM and all substrates at 1 mM concentration (in the case of strategy 3 competition assays, the substrate concentration of α-substituted acyl-CoA derivatives was reduced to 0.5 mM to prevent inhibition by high substrate concentrations). The assays additionally contained 5 mM NADPH, 0.1 mM CoA, 5 mM $MgCl_2$ and 100 mM NaH_2PO_4, pH 7.2. The assays were prepared on ice and 10 µL sample was mixed with 1:1 MeOH and stored at -80 °C as the negative sample. The assay was run at 25°C overnight, stopped with 1:1 MeOH and stored at -80 °C until analysis. Epimerase, to yield in the production of (2*S*)-acyl-malonyl-CoA derivatives, was added but did not have an impact on product spectrum and was therefore left out. UPLC-high resolution MS analysis was carried out using an Agilent 6550 iFunnel Q-TOF LC-MS system equipped with an electrospray ionization source set to positive ionization mode. The analyte was separated on a RP-18 column (50 mm x 2.1 mm, particle size 1.7 µm, Kinetex EVO C18, Phenomenex) using a mobile phase system comprised 0.1% formic acid in water (Solvent A) and acetonitrile (Solvent B). Chromatographic separation was carried out using the following gradient condition at a flow rate of 250 µL/min: 0 min 5% B; 1 min 5% B, 6 min 95% B; 6.5 min 95% B; 7 min 5% B. The column oven was set to 40 °C and auto sampler was maintained at 8 °C. Standard injection volume was 10 µL. Capillary voltage was set at 3.5 kV and nitrogen gas was used as nebulizing (20 psig), drying (13 L/min, 225 °C) and sheath gas (12 L/min, 40 °C). MS data were acquired with a scan range of 50-1200 *m/z*. LC-MS data were analyzed using MassHunter Qualitative Analysis software (Agilent).

Acyltransferase steady state kinetic assay. The assay buffer (200 mM Tris, glycerol 5%v/v, 20 mM $MgCl_2$, pH 7.4) was used to set up all other solutions for this assay. Four master mixes (MM1 to MM4) in 4x concentration were prepared. For MM1 containing the KS-AT proteins (KS-AT or KS-$AT^{S\text{->}A}$ mutants), the enzymes were adjusted to 2 µM (for transacylation) or to 4 µM (for hydrolysis) with assay buffer additionally containing 1 mM DTT and 0.1 mg/mL BSA. For MM2 highly pure and concentrated ACP were adjusted to 1600 µM concentration with the assay buffer. Substrate acyl-CoA containing MM3 was prepared with the assay buffer in varying substrate concentrations ranging from (4x concentration indicated here) 1 µM to 1 mM. MM4 contained 1.6 mM NADH, 20 mM ATP, 30 mM succinate, 10 mM phosphoenolpyruvate (stock solutions should be resuspended in assay buffer, or Tris 200 mM, pH 7.4). First, these compounds were mixed and assay buffer was added, leaving volume for Pyruvate Kinase/Lactic Dehydrogenase (PK/LDH), SucC and SucD. After mixing of the compounds, the enzymes were added to MM4 like following: 0.06 mM SucC, 0.06 mM SucD and 20% v/v of PK/LDH (Sigma-Aldrich) (40 µL PK/LDH for 200 µL MM4). To measure transacylation 25 µL MM3, 25 µL MM4, 25 µL MM2 were mixed and incubated 30 °C for one minute. The reaction was started using 25 µL MM1. To measure hydrolysis 25 µL MM3, 25 µL MM4, 25 µL assay buffer were mixed, incubated at 30 °C for one minute. The reaction was started with 25 µL MM1. In both cases after starting the reaction, the mixture was immediately transferred into a 1 cm quartz cuvette, preheated to 30 °C and the absorption was monitored at 340 nm with a Cary 60 UV vis (Agilent technologies). The reactions were measured in three to four technical replicates. Simultaneous to the transacylation/hydrolysis reaction a negative control was measured. For transacylation, MM1 was replaced by assay buffer (MM2 caused the highest background activity), for hydrolysis MM3 was replaced by assay buffer. For the transacylation reaction the linear slope during the first minute (seconds 30-60) was used for measurement, for the hydrolysis the linear slope during the first two minutes. The slope of the negative control was subtracted of the slopes measured for the reactions. Prior to measuring the single kinetics of each KS-AT for each substrate, the KS-AT concentration was halved and the velocity was calculated, to assure measurements where the assay is not rate limiting.

HPLC analysis of ACP loading by KS-AT. Loading of *holo*-ACPs was initiated by adding KS-AT with a final concentration of 5 µM to the cognate ACP 100 µM and a single acyl-CoA ester 300 µM. Phosphopantetheinylation of *apo*-ACPs by the 4'-phosphopantetheinyl transferase was initiated by adding Npt with a final concentration of 1 µM to a mix containing 100 µM ACP, 5 mM $MgCl_2$, 200 mM NaH_2PO_4 and 1.5 µM CoA. The reactions were run at 25 °C and stopped by addition of formic acid and injected onto a Discovery BIO Widepore C5 HPLC column. Solvents contained 0.1% TFA. Solvent A

was distilled water and Solvent B was acetonitrile. Chromatographic separation was carried out using following gradient condition at a flow rate of 1.5 mL/min: 0 min 5% B; 3 min 35% B; 28 min 50% B; 29 min 95% B; 30 min 5% B. Colum temperature was 40 °C. ACP elution was monitored at 220 nm wavelength.

HPLC kinetic analysis of PnC_KS-AT. The assay contained final concentrations of 100 µM PnC *holo*-ACP, 5 µM PnC KS-AT, 300 µM acyl-CoA thioester and 50 µM NaH_2PO_4, pH 7.2. Reactions were run at 25 °C and stopped by addition of formic acid and injected onto a Discovery BIO Widepore C5 HPLC column. Solvents used were distilled water (Solvent A) and acetonitrile (Solvent B), both containing 0.1% TFA. Chromatographic separation was carried out using following gradient condition at a flow rate of 1.5 mL/min: 0 min 5% B; 3 min 35% B; 28 min 50% B (for butyl-, 3-methylbutyl- and hexylmalonyl-CoA)/ 3 min 36,5% B; 28 min 37,8% B (for malonyl-CoA)/ 3 min 35% B; 28 min 43% B (for ethylmalonyl-CoA). Followed by 29 min 95% B; 30 min 5% B. Colum temperature was 40 °C. ACP elution was monitored at 220 nm wavelength. Time points were measured in technical triplicates. The linear increase during the first minutes was used to calculate the transacylation rate.

Mass spectrometric measurements of standalone ACPs. Loading of *holo*- and *apo*-ACPs by KS-AT and Npt was done as described above. All reactions were run at 25 °C for 2 h and placed on 4 °C until analysis by mass spectrometry. 2 µL of the buffered protein solutions were desalted online using a Waters ACQUITY H-Class HPLC-system equipped with a MassPrep column (Waters). Desalted proteins were eluted into the ESI source of a Synapt G2Si mass spectrometer (Waters) by the following gradient of buffer A (water with 0.05% formic acid) and buffer B (acetonitrile with 0.045% formic acid) at a column temperature of 60 °C and a flow rate of 0.1 mL/min: Isocratic elution with 5% A for two minutes, followed by a linear gradient to 95% B within 8 minutes and holding 95% B for additional 4 minutes. Positive ions within the mass range of 500-5000 *m/z* were detected. Glu-Fibrinopeptide B was measured every 45 s for automatic mass drift correction. Averaged spectra were deconvoluted after baseline subtraction and eventually smoothing using MassLynx instrument software with MaxEnt1 extension.

Synthesis of acyl-CoAs. Acyl-CoA synthesis was done like previously described. CHC-CoA was synthesized by chemical CDI coupling of the free acid, analogous as previously explained [6].

Construction of plasmids used in this study. The coding sequence for the phoslactomycin polyketide synthase genes from Streptomyces platensis was synthesized and codon optimized by the Joint Genome Institute. Inserts were amplified by PCR. Oligonucleotides with overhangs for the restriction enzymes NdeI (CATATG) and HindIII (AAGCTT) were used and the agarose gel purified fragments were ligated into the cloning vector pJET1.2. From there the insert was cut out with NdeI and HindIII and inserted into expression vectors. SucC (Accession number WP_113400154) and SucD (Accession number WP_096861694) coding sequence was amplified by colony-PCR from *E.coli* strain Top10 and cloned following the procedure above. Npt coding sequence was codon optimized for *E.coli* and synthesized. All cloning was done using the *E.coli* strain Top10. Catalytic knock out mutants were constructed using mismatching oligonucleotides, containing the DNA triplet for the desired amino acid. The plasmid was divided into two or three parts and amplified by PCR using Q5® High-Fidelity DNA polymerase. The purified fragments were assembled using the Gibson Assembly ® Master Mix purchased from New England Biolabs.

List of plasmids used in this study

Vector	Insert	His_6-tag	Protein size (kDA)	Extinction coefficient (ε)
pET21a(+)	PnA_{F4}	C-terminal	251.74	251.79
pET21a(+)	PnB	C-terminal	354.42	401.39
pET21a(+)	PnB^{Y3074F}	C-terminal	354.39	399.90
pET21a(+)	$PnB\text{-}TE_{DEBS}$	C-terminal	374.95	437.96
pET21a(+)	PnC	C-terminal	176.81	202.08

pET21a(+)	PnC ^{Y1367F}	C-terminal	176.79	200.59
pET21a(+)	PnC-TE_{DEBS}	C-terminal	198.99	238.65
pET21a(+)	PnC^{Y1367F}-TE_{DEBS}	C-terminal	198.98	237.16
pET21a(+)	PnD	C-terminal	197.11	172.40
pET21a(+)	PnD-TE_{DEBS}	C-terminal	221.18	202.10
pET21a(+)	Npt	C-terminal	25.78	36.11
pET28b(+)	PnB-KSAT1	N-terminal	97.19	102.25
pET28b(+)	PnB^{S669A}-KSAT1	N-terminal	97.17	102.25
pET28b(+)	PnB-ACP1	N-terminal	12.77	/
pET28b(+)	PnB-KSAT2	N-terminal	96.76	89.76
pET28b(+)	PnB^{S671A}-KSAT2	N-terminal	67.74	89.76
pET28b(+)	PnB-ACP2	N-terminal	12.77	/
pET28b(+)	PnC-KSAT	N-terminal	101.32	114.74
pET28b(+)	PnC^{S684A}-KSAT	N-terminal	101.30	114.74
pET28b(+)	PnC-ACP	N-terminal	12.01	/
pET28b(+)	PnD-KSAT	N-terminal	100.57	85.75
pET28b(+)	PnD^{S681A}-KSAT	N-terminal	100.55	85.75
pET28b(+)	PnD-ACP	N-terminal	13.84	/
pET28b(+)	SucC	N-terminal	43.56	27.18
pET28b(+)	SucD	N-terminal	31.94	9.19

Protein expression and purification. Expression constructs encoding for proteins with an ACP domain were transformed into *E.coli* BAP1 or BL21 (DE3) competent cells, all other constructs were transformed into *E.coli* BL21 (DE3) and supplemented with the respective antibiotics. Overnight cultures of 10 mL LB were inoculated with a single colony and used for inoculation of 1 L Terrific Broth medium. Cells were grown at 37 °C until they reached an OD_{600} of 1, induced with 100 µM isopropyl-β-D-thiogalactopyranoside and PKS proteins were expressed at 16 °C shaking at 90 rpm for approximately 15 h. Cells were harvested, immediately used for purification or stored at -20 °C. The other proteins were expressed at 18 °C. SucC and SucD were expressed at 23 °C.

Protein Nickel NTA-purification. Cell pellets were resuspended in Buffer A (500 mM NaCl, 50 mM NaH_2PO_4, 10% v/v glycerol, pH 7.5) and lysed by sonication, centrifuged at 42.000 g, 4 °C for 45 min and the supernatant was mixed with 3.5 mL Protino® Ni-NTA Agarose purchased from Macherey Nagel and incubated for 2 h on ice with slow shaking. The beads solution was transferred into Protino® Columns 14 mL, washed with 50 mL Buffer A. Following the beads were washed with 20 mL washing buffer (25 mM Imidazole, 500 mM NaCl, 50 mM NaH_2PO_4, 10% v/v glycerol, pH 7.5). Proteins were eluted with 8 mL Buffer B (500 mM Imidazole, 500 mM NaCl, 50 mM NaH_2PO_4, 10% v/v glycerol, pH 7.5). SucC, SucD, Npt and all standalone ACP proteins were further purified by size exclusion using HiLoad 16/600 Superdex 200 pg column (300 mM NaCl, 25 mM NaH_2PO_4, pH 7.5). All other proteins were subjected to anion exchange. Protein concentration was determined by UV-vis measurements at 280 nm, in the case of ACPs concentration was determined by Bradford assay.

Anion exchange. The eluate from the Nickel beads purification was diluted to 120 mL with Anion A (50 mM NaH_2PO_4, pH 7.5) and loaded onto a 5 mL HiTrap Q HP anion exchange 5 mL chromatography column, purchased from GE Healthcare Life Sciences, with a flow of 3 mL/min. A gradient to 100% Anion B (50 mM NaH_2PO_4, 500 mM NaCl pH 7.5) with a flow of 4 mL/min in 30 min was run and protein containing fractions were collected and concentrated using Amicon® Ultra Centrifugal Filters, purchased from Merck Millipore. Proteins were used immediately for assays or stored with 30% v/v glycerol in -80 C after shock freezing in liquid nitrogen.

Synthesis of (2*Z*)-cylcohexanepropenyl-SNAC

General Information. All non-aqueous reactions were carried out using flame-dried glassware under argon atmosphere. All solvents were distilled by rotary evaporation. Solvents for non-aqueous reactions were dried as follows prior to use: Tetrahydrofuran (THF) was dried with potassium hydroxide and subsequently distilled from Solvona®. Dichloromethane and triethylamine were distilled from calcium hydride. *N*-acetylcysteamin (HSNAC) was synthesised according to literature [7]. All other commercially obtained reagents were used as received. Reactions were monitored by thin layer chromatography (TLC) using Merck Silica Gel 60 F_{254}-plates and visualised by fluorescence quenching under UV-light. In addition, TLC-plates were stained using a potassium permanganate stain. Chromatographic purification of products was performed on Merck Silica Gel 60 (230-400 mesh) unless otherwise noted using a forced flow of eluents. Concentration *in vacuo* was performed by rotary evaporation at 40 °C and appropriate pressure and by exposing to fine vacuum at room temperature if necessary. NMR spectra were recorded on a Bruker AV 300 MHz, AV III 500 MHz, AV III HD 500 MHz spectrometer at room temperature. Chemical shifts are reported in ppm with the solvent resonance as internal standard. Data are reported as follows: s = singlet, d = doublet, t = triplet, q = quartet, quint = quintet, m = multiplet. Mass spectra were recorded by the mass service department of the Philipps-Universität Marburg. HR-ESI mass spectra were acquired with an LTQ-FT mass spectrometer (Thermo Fischer Scientific). The resolution was set to 100 000. IR spectra were recorded on a Bruker IFS 200 spectrometer. The absorption bands are given in wave numbers (cm^{-1}), intensities are reported as follows: s = strong, m = medium, w = weak, br = broad band. Melting points were determined on a Mettler Toledo MP70 using one end closed capillary tubes. Optical rotations were determined at 20 ^{o} with a Krüss P8000-T polarimeter.

Synthesis of methyl (*Z*)-3-cyclohexylacrylate (9)

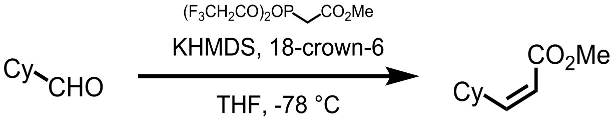

10 9

To a solution of Still-Gennari-reagent (0.64 mL, 3.03 mmol, 1.20 eq.) and 18-crown-6 (0.77 g, 2.90 mmol, 1.15 eq.) in tetrahydrofuran (15 mL) at 0 °C under an argon atmosphere was added a solution of potassium bis(trimethylsilyl)amide (0.5 M in toluene, 5.80 mL, 2.90 mmol, 1.15 eq.) dropwise. After 30 min the solution was cooled to -78 °C and aldehyde **10** (0.30 mL, 2.52 mmol, 1.00 eq.) was added dropwise. The solution was stirred 1 h at -78 °C and afterwards reaching room temperature overnight. A sat. solution of ammonium chloride and diethyl ether were added, the layers separated and the aqueous was extracted with diethyl ether (3x). The combined extracts were dried (magnesium sulphate), the volatiles removed *in vacuo* and the residue was purified by flash chromatography (silica gel, pentane/diethyl ether 50:1) to give (*Z*)-ester **9** (0.37 g, 2.20 mmol, 87%) as colourless liquid. TLC: R_f = 0.40 (pent/diethyl ether 50:1); ^{1}H-NMR (500 MHz, $CDCl_3$): δ = 6.03 (dd, J = 11.5, 10.0 Hz, 1H, CyC*H*=CH), 5.66 (dd, J = 11.5, 1.0 Hz, 1H, CyCH=C*H*), 3.70 (s, 3H, OCH_3), 3.34-3.25 (m, 1H, Cy-C*H*), 1.74-1.64 (m, 5H, Cy-CH_2), 1.40-1.30 (m, 2H, Cy-CH_2), 1.23-1.13 (m, 1H, Cy-CH_2), 1.12-1.03 (m, 2H, Cy-CH_2) ppm; ^{13}C-NMR (125 MHz, $CDCl_3$): δ = 166.8 (*C*O_2Me), 156.1 (Cy*C*H=), 117.1 (CyCH=*C*H), 51.0 (O*C*H_3), 37.3 (Cy-*C*H), 32.3 (2xCy-*C*H_2), 25.9 (Cy-*C*H_2), 25.4 (2xCy-*C*H_2) ppm; HRMS (ESI+): *m/z* calcd. for $C_9H_{13}O$ $[M+Na]^+$ 191.1043, found 191.1042; υ_{max}(film): 2965 (w), 2926 (s), 2854 (w), 2358 (m), 2035 (m), 2029 (w), 1728 (s), 1193 (w), 1176 (m), 483 (m), 453 (m), 439 (m), 424 (s) cm^{-1}.

Synthesis of (*Z*)-3-cyclohexylacrylic acid (8*)*

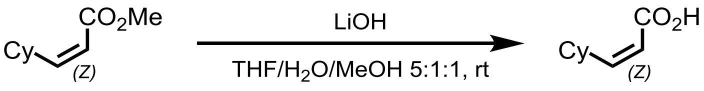

9

8

To a solution of (*Z*)-ester **9** (104 mg, 0.62 mmol, 1.00 eq.) in a 5:1:1 mixture of tetrahydrofuran/water/methanol (7 mL) at room temperature was added lithium hydroxide monohydrate (259 mg, 6.18 mmol, 10.0 eq.) and the mixture was stirred overnight. After addition of water the pH-value was adjusted (pH=3) with hydrochloric acid (1 M), the mixture extracted with diethyl ether (3x) and the combined extracts were dried (magnesium sulphate). The volatiles were removed *in vacuo* and the residue purified by flash chromatography (silica gel, pentane/ethyl acetate 3:1) to give (*Z*)-acid **8** (80 mg, 0.52 mmol, 84%) as white solid. TLC: $R_f = 0.55$ (pent/ethyl acetate 1:1); ^{1}H-NMR (500 MHz, $CDCl_3$): $\delta = 6.15$ (dd, $J = 11.3$, 10.2 Hz, 1H, Cy-C*H*=), 5.67 (d, $J = 11.3$ Hz, 1H, Cy-CH=C*H*), 3.33-3.26 (m, 1H, Cy-C*H*), 1.73-1.64 (m, 5H, Cy-CH_2), 1.42-1.30 (m, 2H, Cy-CH_2), 1.25-1.06 (m, 3H, Cy-CH_2) ppm (CO_2H was not detectable due to fast exchange with the solvent); ^{13}C-NMR (125 MHz, $CDCl_3$): $\delta = 170.0$ (*C*O_2H), 158.1 (Cy-*C*H=), 116.6 (Cy-CH=*C*H), 37.4 (Cy-*C*H), 32.3 (2xCy-*C*H_2), 25.9 (Cy-*C*H_2), 25.4 (2xCy-*C*H_2) ppm (*C*O_2H was not detectable due to low C_q-intensity); HRMS (ESI−): *m/z* calcd. for $C_9H_{13}O_2$ $[M]^-$ 153.0921, found 153.0921; υ_{max}(film): 2926 (s), 2852 (w), 2734 (w), 2575 (w), 1694 (s), 1638 (w), 1439 (m), 1351 (w), 1290 (w), 1270 (w), 1242 (m), 1215 (w), 1135 (w), 1029 (w), 965 (w), 931 (w), 892 (w), 827 (w), 798 (w), 729 (w), 695 (w), 451 (w) cm^{-1}; m.p.: 106.5 °C (dichloromethane).

Synthesis of *S*-(2-acetamidoethyl) (*Z*)-3-cyclohexylprop-2-enethioate (7)

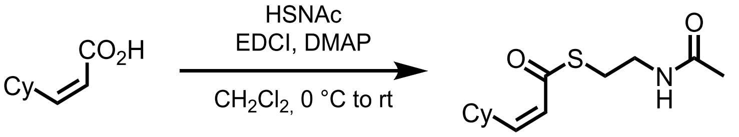

8

7

To a solution of acid **8** (20 mg, 0.13 mmol, 1.00 eq.) in dichloromethane (2 mL) at 0 °C under an atmosphere of argon were added *N*-acetylcysteamine (62 mg, 0.52 mmol, 4.00 eq.), 2,2-dimethylaminopyridine (cat.) and 1-ethyl-3-(3-dimethylaminopropyl)carbodiimide hydrochloride (40 mg, 0.21 mmol, 1.60 eq.) successively. The mixture was stirred reaching room temperature over night after which water was added. The aqueous layer was extracted with dichloromethane (3x), the combined extracts were dried (magnesium sulphate) and the volatiles removed *in vacuo*. The residue was purified by flash chromatography (silica gel, pentane/ethyl acetate 1:1) to give SNAC-ester **7** (30 mg, 0.11mmol, 82%) as pale yellow oil. TLC: $R_f = 0.17$ (pent/ethyl acetate 1:1); ^{1}H-NMR (500 MHz, $CDCl_3$): $\delta = 5.97$ (dd, $J = 11.3$, 0.67 Hz, 1H, Cy-C*H*=), 5.87 (dd, $J = 11.3$, 9.7 Hz, 1H, Cy-CH=C*H*), 5.83 (bs, 1H, N*H*), 3.47 (q, $J = 6.2$ Hz NCH_2), 3.23-3.15 (m, 1H, Cy-C*H*), 3.08 (t, $J = 6.2$ Hz 2H, SCH_2) 1.97 (s, 3H, CH_3), 1.75-1.64 (m, 5H, Cy-CH_2), 1.37-1.29 (m, 2H, Cy-CH_2), 1.22-1.06 (m, 3H, Cy-CH_2) ppm; ^{13}C-NMR (125 MHz, $CDCl_3$): $\delta = 189.8$ (O*C*S), 153.1 (Cy-*C*H=), 124.0 (Cy-CH=*C*H), 39.8 (N*C*H_2), 38.4 (S*C*H_2), 32.2 (2xCy-*C*H_2), 28.6 (Cy-*C*H), 25.8 (Cy-*C*H_2), 25.4 (2xCy-*C*H_2) ppm; HRMS (ESI+): *m/z* calcd. for $C_{13}H_{21}NO_2SNa$ $[M+Na]^+$ 278.1185, found 278.1187; υ_{max}(film): 3283 (w), 3080 (w), 2924 (s), 2850 (w), 1672 (w), 1653 (s), 1623 (w), 1550 (m), 1446 (w), 1408 (w), 1373 (w), 1289 (w), 1262 (w), 1197 (w), 1099 (w), 1000 (s), 951 (s), 891 (w), 830 (m), 803 (w), 776 (w), 600 (w), 552 (w), 535 (w) cm^{-1}.

NMR-Spectra

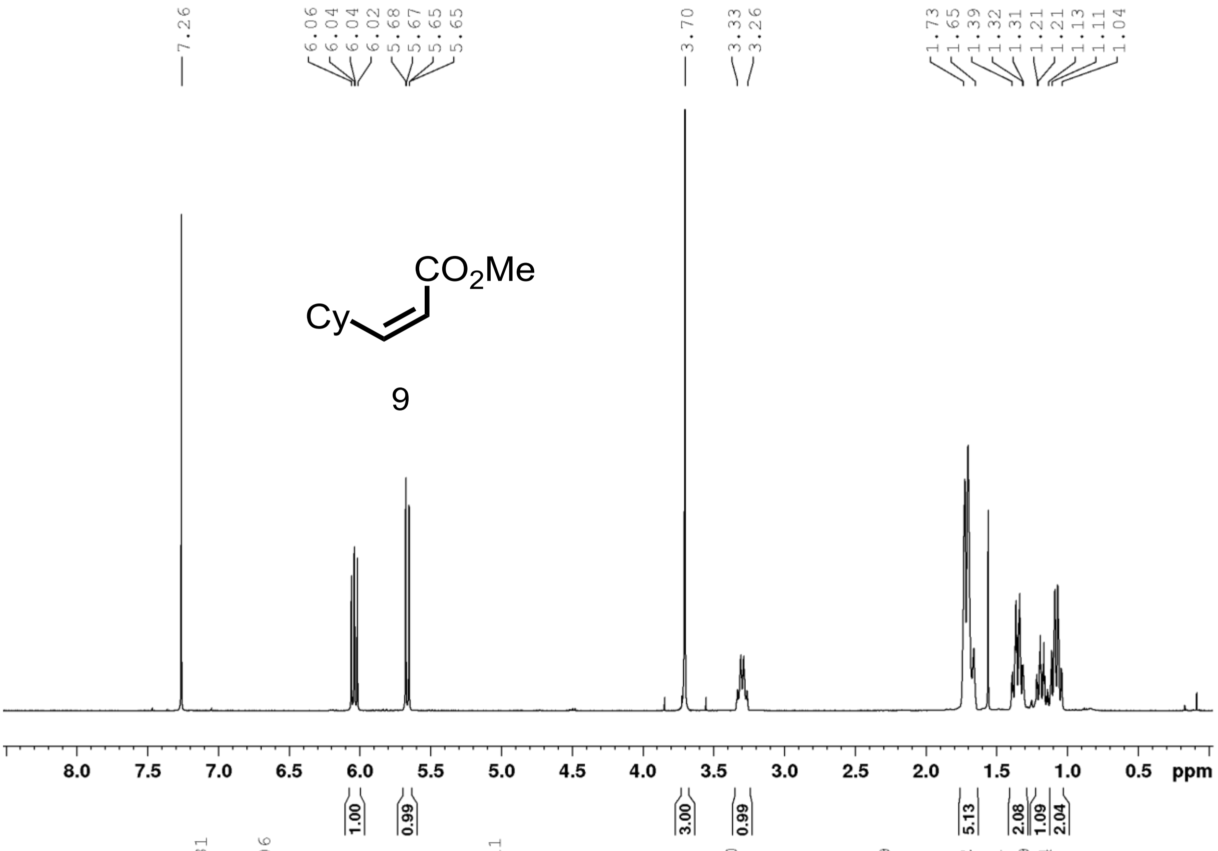
7.26
6.06 6.04 6.04 6.02 5.68 5.67 5.65 5.65
3.70
3.33 3.26
1.73 1.65 1.39 1.32 1.31 1.21 1.21 1.13 1.11 1.04
CO2Me
Cy
9
8.0 7.5 7.0 6.5 6.0 5.5 5.0 4.5 4.0 3.5 3.0 2.5 2.0 1.5 1.0 0.5 ppm
1.00 0.99 3.00 0.99 5.13 2.08 1.09 2.04

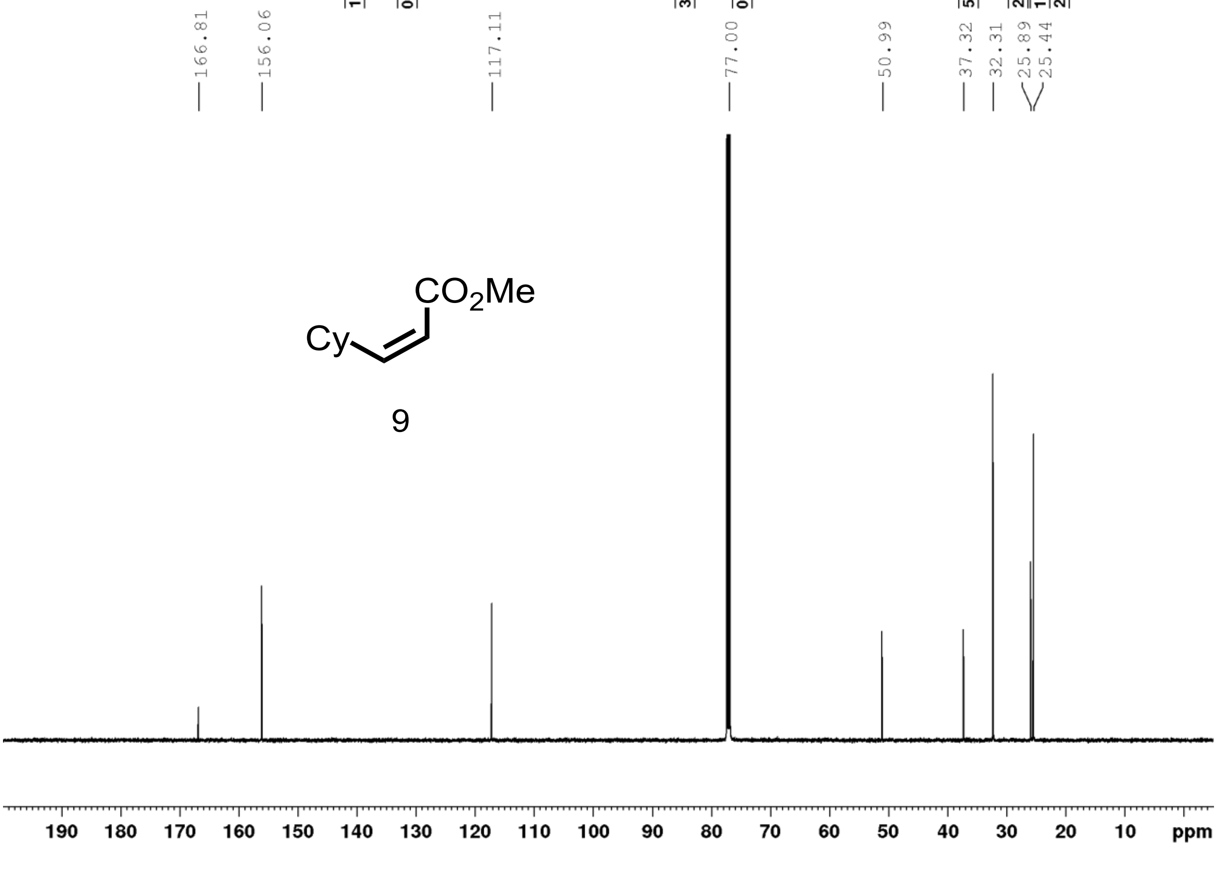
166.81
156.06
117.11
77.00
50.99
37.32
32.31
25.89 25.44
CO2Me
Cy
9
190 180 170 160 150 140 130 120 110 100 90 80 70 60 50 40 30 20 10 ppm

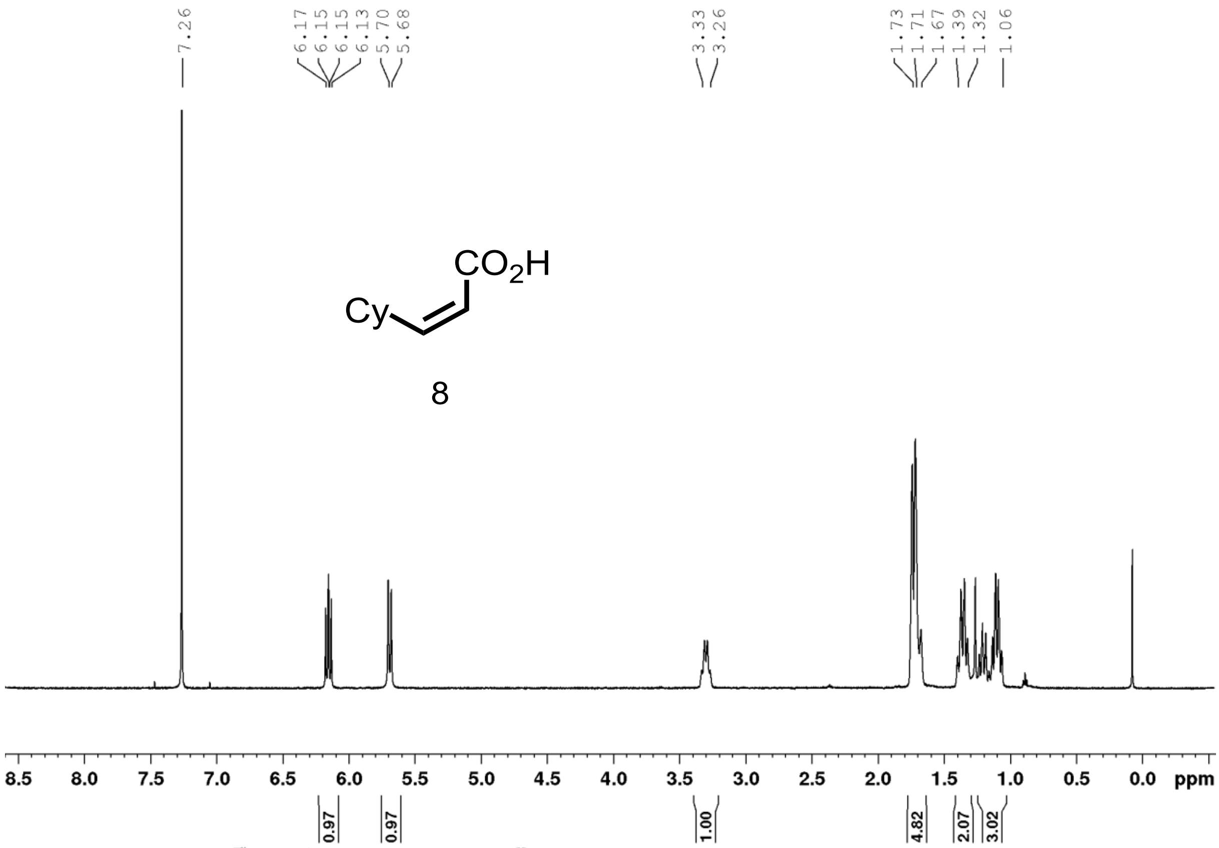
7.26
6.17
6.15
6.15
6.13
5.70
5.68
3.33
3.26
1.73
1.71
1.67
1.39
1.32
1.06
CO2H
Cy
8
8.5 8.0 7.5 7.0 6.5 6.0 5.5 5.0 4.5 4.0 3.5 3.0 2.5 2.0 1.5 1.0 0.5 0.0 ppm
0.97
0.97
1.00
4.82
2.07
3.02

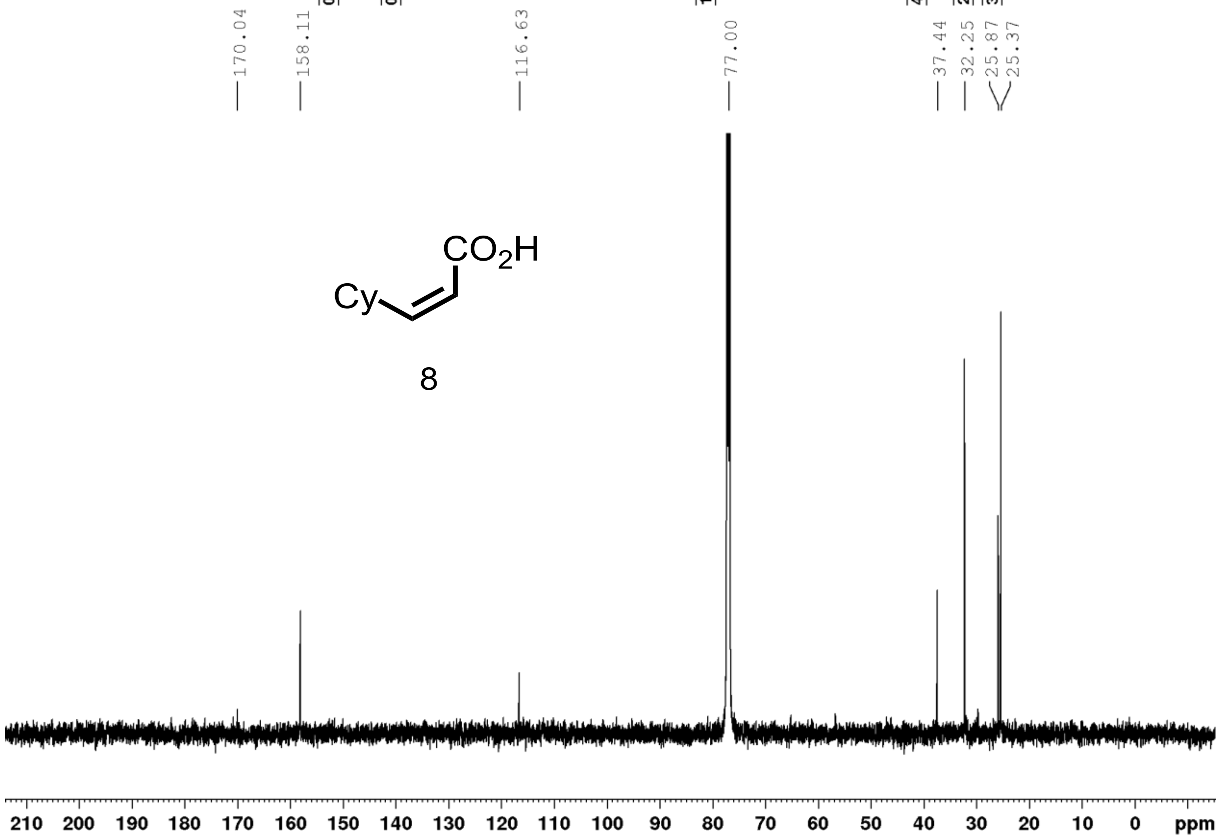
170.04
158.11
116.63
77.00
37.44
32.25
25.87
25.37
CO2H
Cy
8
210 200 190 180 170 160 150 140 130 120 110 100 90 80 70 60 50 40 30 20 10 0 ppm

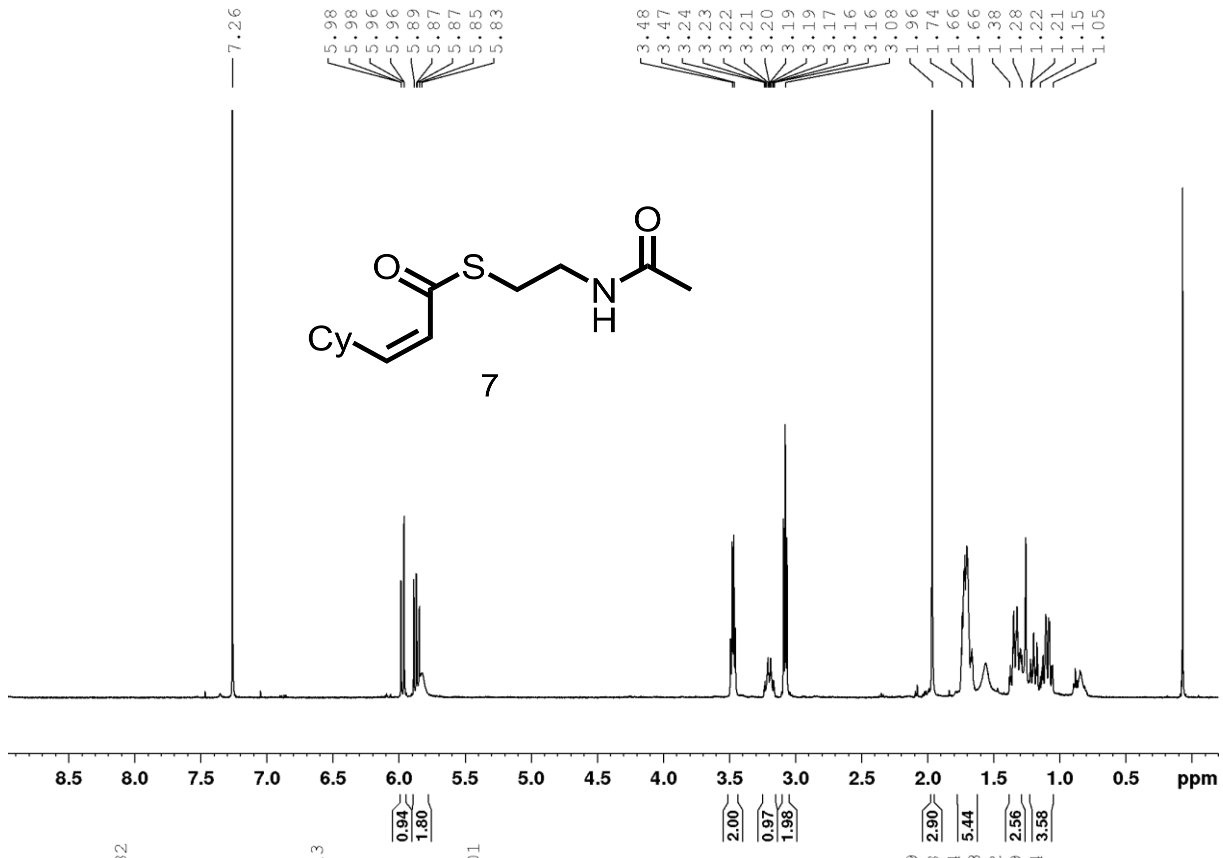
7.26
5.98 5.98 5.96 5.96 5.89 5.87 5.87 5.85 5.83
3.48 3.47 3.24 3.23 3.22 3.21 3.20 3.19 3.19 3.17 3.16 3.16 3.08
1.96 1.74 1.66 1.66 1.38 1.28 1.22 1.21 1.15 1.05
O S N H O
Cy
7
8.5 8.0 7.5 7.0 6.5 6.0 5.5 5.0 4.5 4.0 3.5 3.0 2.5 2.0 1.5 1.0 0.5 ppm
0.94 1.80 2.00 0.97 1.98 2.90 5.44 2.56 3.58

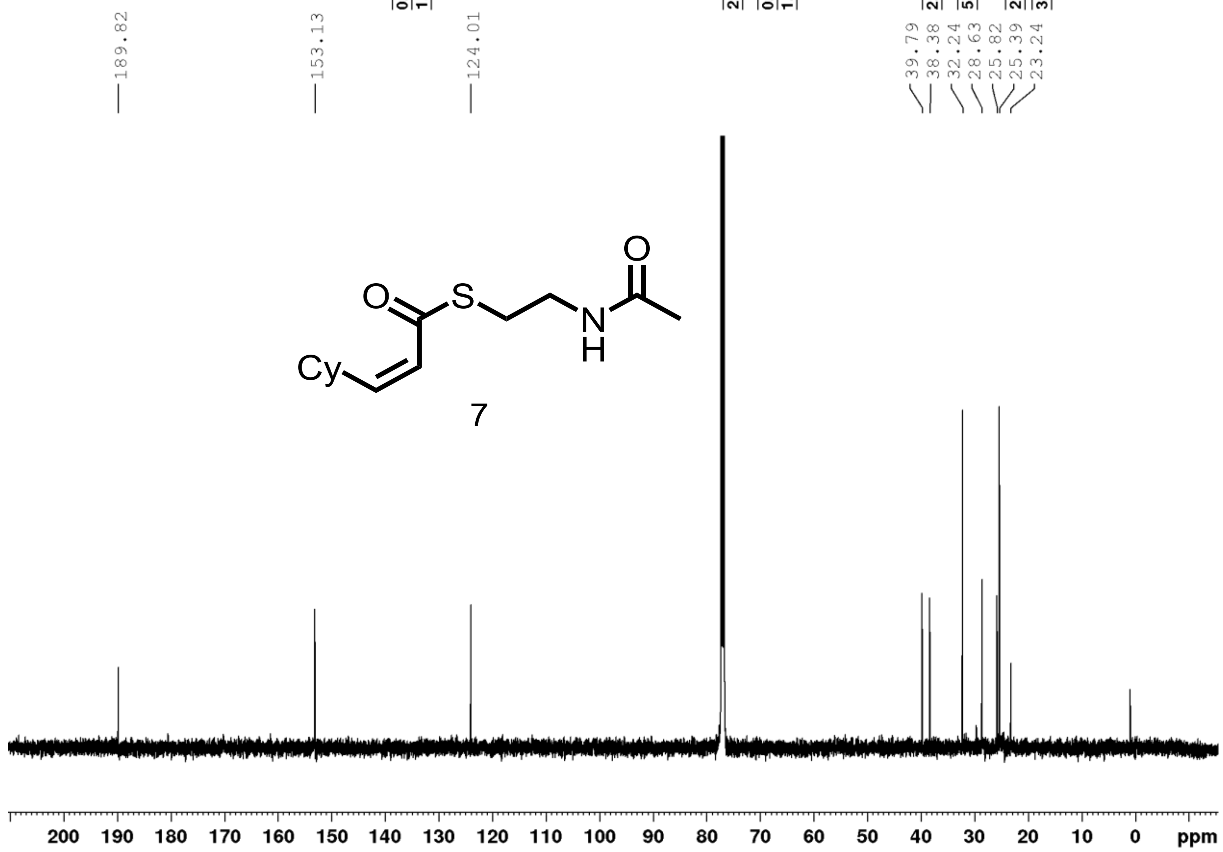
189.82
153.13
124.01
39.79 38.38 32.24 28.63 25.82 25.39 23.24
O S N H O
Cy
7
200 190 180 170 160 150 140 130 120 110 100 90 80 70 60 50 40 30 20 10 0 ppm

DNA sequences of the constructs used for this study. The in this study used KS-AT and ACP constructs are indicated by caption in the following DNA sequences. Marked in bold is the module linker sequence that is replaced with DEBS_TE in the cases of PnB, PnC and PnD for PnB-TE$_{DEBS}$, PnC-TE$_{DEBS}$ and PnD-TE$_{DEBS}$ constructs.

Insert	Domain	Coding DNA Sequence
**PnA$_{V4}$ (PnA Accession number AFJ05078)**		ATGGTAGCTGTAGCACTCGCACTGGAAACCGCTGATGCGGAACATTACGG<br>CCCACTGTCCGACGACGCTGAAGAACGCGTTGCAGAGCTGCGTGCACTGC<br>GCCCTCGTGACCGCACCTCCGCTCCGGATGCTACGTCTACCTGCGCAGTAC<br>CGCTGCTGCTGTCTGGTCGCACTGACGCAGGCCTGCGTGCTCACGCAGGTC<br>GTCTGGCAGGTCATCTGCAAGAACGTACAGACATCTCCCTGTCTGATGCA<br>GCATTCACCCAGGCTACTACTCGCACTCTGTGGCCGCACCGTGCAATGGTG<br>ACCGCAGCCGACCGCGACGCTGTTGTTGCTGCGCTGCGCGCTGTTGCCGA<br>AGGTCGTGCTTCTGACGCAGCTGTACGCGCTGGTAGTGGTGGTCCGGAAT<br>CCGCTGTTTTCGTTTTCCCGGGCCAGGGTGCGCAGTGGGTAGGCATGGCAC<br>GTGAACTGGCTGCTGCCGCTCCGGTATTCCGTGACAAACTGGCTGAGTGTG<br>CTCGTGAACTGCGCCCGTTCGTTGACTTCGAACTGGACGACGTTCTGTCTG<br>GTGCCCTGCCGCTGGAACGTGTTGAAGTTATCCAGCCGGCTCTGTTCGCCG<br>TAATGGTTAGCCTGGCGGAACTGTGGCGTTCCAACGGCGTTACGCCGGCT<br>GCTGTTGTGGGTCACTCTTTCGGTGAAATCGCTGCGGTTACTGCGGCAGGC<br>GCTCTGACACTCGCAGACGGCGCGCGTCTGGTTGCTGCTGTATCCAAAGCT<br>CTGGCACAACTGCAAGGTAACGGCGAGATGGTTGCAGTAGCGCTGCCGGA<br>TGCTCAGGTAACCGAACTGGTTGCAGAATGGGACCTGGACCTGGACATCG<br>CAGTTGTAAACGGCCCGCGTTCTACCGTGGTAGCTGGCCCAACCGAAGCA<br>GCTACCGCTCTGGTTGAACGCCTGCGCGACCGCGATGTACGTGCAACTCTG<br>CTGCCTATCGGTATCGCTGGTCACAGCCGCCGTATGGAACCGGCACACGC<br>ATACCTGGTACAGGAGGCGTCTGCTGTCCGTCCGCGTGCTACTGGTATCCC<br>AGTATACACATCTACCACTACCGACCCGCTGGACACTGGTGACCTCGACG<br>CCGAACACTGGTTTCATTCTCTGCGCGAACCAGCACGTTTCCAGCAGGTTA<br>TCGAAGAGCTGCTGGGTCAGGGTCACCGCGTATTCGTAGAAATGTCCCCG<br>CATCCGGTTCTGGCTCTGTCTATCGAAGAAACCGCAGCTCACCTGGGTCGT<br>GATGTAGTAGTTCTGGAAACGATGCGTCGTGACGATGCGGGTCACGACCG<br>TTATCTGCGTGCTCTGGCGGAAGCACATCTGCACGGTGTTGCGCCGGACTG<br>GTCTACTGTTCTGCCGGATGCTCGTCGTGTGACTCTGCCGCCGTACCGTCT<br>GGACCTGGACACAGCTGACGCTGCTGGCTCTGGCGGCACCGAACCGGGTG<br>CCGGTCTCCGTGAACGCCTGCTGGGCGTTGCACCTGAACGTCGCATCGAA<br>GAAGCCGTGCGTCTGGTTGTTGACGCTGTGGCAGCACACATCGAACCGTC<br>TGCTACAGCTATTGGTGCAGACCAGGCATTCCGTAGCTTAGGCGTTGACTC<br>CGCTGGCGCACTGCGTGTGCGCAACCGTCTGGTAGAAACTACCGGCCTGC<br>GCCTGCCGGCGACTATCCTGTTCGACCACCCGACCCCGCGTGCTCTGGCCG<br>AAGAGCTGGTTCGCGCTGTACTGGGCGGTGACGAACCGGCACCGGCAGCT<br>ACCGCTCCGGCTGACCCGGACGAGCCGCTGGCAATCGTAGGTATGGCGTG<br>CCGTTTTCCAGGTGGTGTGGGTAGCCCGGACGAACTGTGGCAGCTCGTAC<br>ACGAAGGCGGGGATGCCGTTGCGGCCTTCCCGGCAGATCGTGGATGGGAC<br>CTGACCGCTCTGTACGACTCTGATGCCTCCCAGCCGGGCACCTCTTACCAG<br>CGTGAGGCTGCATTACTGGACGGTGTAGCTGAGTTCGACGCTGCATTCTTC<br>GGTATCTCTTCTCGTGAGGCTCTCGCAATGGACCCACAGCAGCGTCTGCTG<br>CTCGAAACTTCTTGGGAGGCTCTGGAACGTGGCGGTATTGACAGCACTAC<br>CCTGCGTGGTTCCCGCACTGGCGTTTTCACTGGTGTTATGTCTCTGCCGTAC<br>GGCCGCCCGCTCCATCAGGCGCGTCCGGACCTGGAGGGTTACGTAATGAC<br>TGGTACAACTTCTTCCGTGGTTTCTGGCCGTCTGTCTTATGTTTTAGGTCTG<br>GAAGGTCCGGCTCTGACTCTGGACACCGCTTGCTCTAGTTCTCTGGTTGCT<br>CTGCACTTGGCAGGTCAGTCCCTGCGTCGTGGTGAATCTGACCTGGCTCTG<br>GTCGGCGGTGCAACTGTGATGGCTGAACCGGGCCTGTTCATCGAGTTCTCT<br>CGTCTGCGCGCTCTGGCTCCGGACGGCCGTAGCAAGCCATTCAGCGCGGA<br>CGCTGACGGTTTCGGCATGGCAGAGGGTGTAGCTGTTCTGGTTGTTGAACG<br>CCTGTCTGACGCACGCCGTCTGGGTCACGACGTACTGGCCGTAGTTCGCGG<br>TTCCGCTGTGAACCAGGACGGCGCTTCTAACGGTCTGACCGCACCGAACG<br>GCCCGGCTCAGCAGCGTGTAATTCGCGCTGCTCTGGACTCCGCAGGTCTGC

		GTGCGGCTGACATCGATGTGGTTGAAGCACACGGCACAGGCACCCGCTTA<br>GGCGACCCGATCGAAGCTCAGGCTCTGATCGCTACTTACGGTAGCGACCG<br>TCCGGCAGAACGTCCGCTGTACGTTGGTTCTCTCAAATCTAACATCGGCCA<br>CGCACAAGCAGCAGCTGGTGTTGCTGGCGTTGTTAAGATGGTTCAGGCCC<br>TCCGTCATGGTGTGATGCCGCGCTCTCTGTATGCGGACAACCCGACCACCA<br>AAGTTGAGTGGTCTTCCGGCCCGGTAGAACTGCTGGCTGAAGCACGTGAA<br>TGGGAGCGCGCAGACGGTCGCCCGCGTCGTGCAGGCGTTTCTGCCTTCGG<br>TATCTCTGGTACTAACGCACACGTGATCCTGGAAGAGTTCGCTCCGGGTAC<br>TCCGCACATCTCTGAGGGTGCTGAATCTGCTGTTCCGTGGGCGGACGTTGC<br>AGTTCCGCTGGTGCTGAGCGGCAAAACCCCGGATGCGGTTCGCGGTCAGG<br>CTCTGGCACTGCGTGAACACCTGGCTTCTCACCCGGAGCTGCCATTACCGC<br>ACGTTGCGCGCGAACTGGCTACCTGCCGTACCCGTTTCGAGCACCGTGCA<br>GTAGTTACTGGTGACCGTGAGGCCGTGCGTGCAGGTCTGGACGACGTTAG<br>CCCAGTTCTGGCTGGCTCTGGCCGCGTAGCGGCAGTGTTCTCTGGTCAGGG<br>TGCGCAGCGTGCAGGCATGGGTCGTCAGCTGGCGGACCATTTCCCGGTTTT<br>TGACGAAGCTCTGAAGGAGGTATGTGGTCACCTCGACCCGGAACTGGGTC<br>GCGGCCTGCGTGAAGTTATGTGGTCTGATGACCCGGAACAGCTGGGTCGT<br>ACCGAGTTCGCTCAGCCGGCTCTCTTCGCATTCGAGGTAGCACTGGCTCGT<br>CTGTGGCAGTCTTGGGGCGTGTCTTTCACCGCAGTTGTTGGTCACTCCGTA<br>GGCGAAATCGCGGCCGCTGTGGTGGCAGGTGTACTGACCGTGCCGGATGC<br>TGCGCGCCTCGTAGTTGCTCGTGGTCGCCTGATGCAGAGCCTGCCGGGCG<br>GCGGTGCCATGTTAGCAGTCGCTGCCGGTGAGGAGGAAGTTACCGCAACT<br>CTGGGCGACTCTGCTCTGGTTGGTATCGCGGCTGTTAACGGCCCAGAAGC<br>AGTTGTGGTTTCTGGTGCTCGTGACGAAGTAGCTCGCGTTGGCGGCATCTG<br>GCGTGAACGTGGTCGTCGTGTGGCTGAGCTGCGCGTTTCCCACGCTTTCCA<br>CTCTCCGCTCATGGAGCCAATCCTGGACGAGTTTCGTGCAGTTGTAGAAAA<br>GCTCCCGTTCCGTCCGCCAACTGTGCCGATCATCGCTACCGCAGACACCGC<br>GCACCCGGTTGACACCGCTGCATATTGGGTAGATCACGCGCGTAACGCTG<br>TACGTTTTGGCGACGCAATCGGTCGCATCCCGGACGCTGACCTGCTGATCG<br>AAGTTGGCCCGGACGCAGCTCTGGCACCGCTGATCGAAACTGGTCACACT<br>GTACTGCCGTCTACCCGTCGCGGTCGTTCTGAAACCCACGCGGTGGTTACC<br>GCTCTGGGTCAGGCACACGCACACGGTGCAGACGTGGACTGGGCGGCACT<br>GCTCCCGCCGGCTCCACGCACCGATCTGCCGACCTACGCATTCCAGCGCC<br>AGCGTTACTGGGACTCTGCTTCTGACGCCACTGCGCGTGTACGTGCAGGTG<br>CTGACCCGCGCCCGCACCCGATGCTGACCTCTCGTACTGACCTGCCGGGTG<br>CCGGTGGTCTGCTGCTGTCTGGTCGCCTGACTCCGGGCTCTGACCCGTGGC<br>TGTCTCATCACGTTGTAATGGGCACCATGCTGCTCCCGGGCACCGGCTTCG<br>TTGAGCTGGCACTGGAGGCTGCACGCTCTGCGGGTGCAGGTTCCGTTGAT<br>GAGCTGGTACTGCGCGCTCCAATGGTGTTCACCGAAGGCCGTCCGCGTGA<br>CCTGCAAGTTTGGGTTGGTCCGGACCAGGGCGATGAACGCGAACTGCAAA<br>TTCGTACACGTGAGACTGGTGGTGACTGGACTCTGCACGCCACTGGTCTGC<br>TGGCTGCACGTACAGCAGACACTGAAGGTTTCCGTGATGGTGACTGGGCA<br>GGTGAGGTATGGCCACCGGCTGGTGCCCAGCAGCTGGTCGGCTCCTCCTTC<br>TACGAAGAACTGGCTGCTCGCGGTTATGAATACGGCCCGGCATTCCACGG<br>TACGAAGGCTCTGTGGGAGCGTGACGGTGACCTGTTTGCTGAGGTTGTACT<br>GCCGGAAGGTCAGCCACGTGGTTTCGGTATCCATCCGGCACTGCTGGACG<br>CTGCACTGCACGCGCTCCCGATCACCGGCTCTCTGTACGAAGCAGGTGAA<br>GTTCGCCTGCCGTTCTCCTTCAACTCTGTGTCTCTGTTCTCCTCTGATGCAC<br>GCCGCCTGCGTGTACGCATTCGTGCTGAGGCTGACAGCGCAGCTGTTTGG<br>ATCACCGACGATGCGGGCTCTCCGGTTCTGGCTATGGAGGGCCTGATCCTG<br>CGCAGCATTGAACGCGCACAGCTGGAAGCAGCTGGTGCTACTGGGCGTAC<br>TGGTTGGTTCTCTGTGACCTGGCGCCAGCTGGCACGCGCAGCTACCACCGA<br>CCGTGTTCCCGGTAACTGGCTGCTCCTGGGTGAAGTCCCGCCGGCTCTGGG<br>TTCTCTGTTCGACGATCCGGTTACCGCAGCGTCTTGGGATCGCTCTACTGC<br>TCCGGACGGTGTTCTGGTGGGCGCTGGTCGTGCAGAAGACCTGCTGGCTG<br>CTCTGCACGAAGTAGCTGGCCACCGTACCGGCCCAGTATGGTGCGTAACC<br>TCAGGTGCAGTAACTGTTGGTACTGACGACCCGGCAGCAGACGTACGTGC<br>TGCCGGTGTTTGGGGTCTGGGTCGTGTAGCAGGCCTGGAACTGCCGGATC<br>GTTGGGGTGGCCTGGTTGACCTGCCGGAACGCATCGACGACGCTACCCGT<br>CGCGCACTGGCTGGCATTCTGACTGGTGACGATGAAGGCGAAGGTGCTGA<br>CGACGCGGCAGAAGACCAGCTGGCGCTGCGTGACGGTTGCCTGTGGGCAC

		GCCGTCTGGTAACTGCTTCTGCCCCGCAAGCCCGCACCTGGACCCCGAAA<br>GGCACTGTTCTGATCACTGGTGGCACGGGTGGCCTGGGTGCGCACGTTGC<br>CCGCCGTATTGCGGAACAGGGCTCTGCTGATCGTATCCTCCTGCTGTCTCG<br>CCAGGGTCCGGCAGCTCCGGGTGCGACCGAACTGCTGGCAGAAATCAGCG<br>CTTTCGGCGCAACTGCTGAAGCCGTAGCTGCCGACGTGACCGACCGCGCA<br>GCAATGGGCGGTCTGCTGGCAACTCTGGCAGCTGAAGGTGCTCCGGTTCG<br>CACCGTTGTACATGCAGCGGGCGTTGTTCGTGACGTGCGTATTGTTGAGAC<br>TGGTGCAGAAGCTCTGGCGGCTCAGATGGCAGCAAAAGTTGAAGGCGCAC<br>TGCTGCTGGATGAACTGCTGCCGGACCTGGACGATTTCGTTCTGTTCTCCA<br>GCATCTCTGGTGTATGGGGTGCGGCTGGTCAGGCTGGTTATGCTGCCGGTA<br>ACGCTTGCCTGGATGCGCTCGCTCGTCGCCGTCGTGAGCAGGGTCGCCCG<br>GCTACCAGCGTTGCATGGGGTCCGTGGGCTGGCGGTGGTATGCTGACTGA<br>ACACGACGAACGTGAACTGCGTAAACGTGGTCTGACTCCGCTGCTGGTTC<br>CGGCTGCTCTGCAAGCAATGGAACAGTCTATTATGGCAGACTCTGCGTGG<br>GACCCGGTAGTGGCGGACATCACCTGGTCTCGTTTCCTGCCGGCCTTCACC<br>GTATCTCGTCCGTCTCCGCTGCTGGGCAGCTTCGAAGAAAAAACTGCACC<br>GGGTCCGCTGGCTCCGTCTGATGGTCGTCGTACCGAAGGCGAGGCCCAGT<br>CTCTGACTCACCGTCTCGCAGCACTGCCGGACGCTGAACGTCTGCCGGTGC<br>TGGCGGAAGTTGTGCGCACCCACGTTGCTGCTCTGATCGGCGAATCTGGCC<br>CGGAACGTGTTGGCCCTGATCGTGCTCTCAAGGAAATCGGTTTCGATAGCC<br>TGATGTCTGTGGAGCTGCGCAACCGCTTCGCTGGTCTGATCGGTGTGAAAC<br>TGCCGGCTACCCTGGTTTTCGACCATCCGACTCCGAACGCACTTGCTGTAC<br>ACCTGCTGGGTCACCTGGACCTGGGCGCAGCTGGTACTGCCACCGCAGAG<br>AAACCGCTGCTGGAAGTTGTTGAACAGATCGAATCTCGCGTTTTATCTCCG<br>CTGACCGACACATCCACTCGTAAGGCACTGGCTGGTCGCTTAACTGAACT<br>GCTGGATCGTCTGGCTGCTCTGGATGCTCGTCCGGTTACTGGCACCGATGG<br>TCTGGCCTCTGCAAGCGCAGCTGAACTGATGCAGTTCATCGACACTGAACT<br>GGGTGACATCTCA
**PnB (Accession number AFJ05079)**	**KS-AT 1**	ATGACCACCGATGAAAGCCAGCTGGTTGAATATCTGCGTAAAGTTACCAC<br>CGATCTGCAAAAAACCCGTAGCCGTCTGCGTGATGCAGAAACCAAACATC<br>ATGAACCGATTGCCATTGTTGGTATTGGTTGTCGTTATCCGGGTGGTGTTC<br>ATGGTGCAGATGATCTGTGGCGTCTGGTTGTTGAAGGTCGTGATGCAATTA<br>GCGGTTTTCCGCAGGATCGTGGTTGGGATCTGGCAGGTCTGTATGATCCTG<br>ATCCGGATGCAGTTGGCACCAGCTATACCCGTGAAGGTGGTTTTCTGACCG<br>GCATTGACCTGTTTGATGCAGATTTTTTTGGTCTGAGTCCGCGTGAAGCAC<br>TGGCAATGGACCCGCAGCAGCGTCTGCTGCTGGAAACCAGCTGGGAAGCA<br>CTGGAAAGCGCAGGTATTCGTCCGGCAACCCTGCGTGGTAGCCGTACAGG<br>TGTTTTTACAGGTGTTATGCATCATGATTATGGTAGCCGTTTTAGCAAAGC<br>ACCGGATGGTTTTGAAGGTTATGTTCACATGGGTAGCGCAGGTAGCATGG<br>CAGTTGGTCGTGTTGCATATCATTTTGGTCTGGAAGGTCCGGCAGTTACCG<br>TTGATACCGCATGTAGCAGCAGCCTGGTTAGCCTGCATCTGGCATGTCAGA<br>GCCTGCGTACAGGTGAATGTGATCTGGCACTGGCAGGCGGTGTTGCAATT<br>ATGGCAGGTCCGACCTTTTTTGTTGAATATAGCCGTCAGCGTGTTCTGAGC<br>ACCGATGGTCGTTGTCATAGCTTTGCAGATGATGGTGATGGTACAGGTTGG<br>AGCGAAGGTGTTGGTGTTCTGGCAGTTGAACGTCTGAGTGATGCACGTCG<br>TCTGGGTCATGATGTTCTGGCCGTTGTTCGTGGTAGCGCAGTTAATCAGGA<br>TGGTGCAAGCAATGGTATGACCGCACCGAGTGGTCCGGCACAGCAGCGTG<br>TTATTGATAGCGCACTGCGTAATGCACGTCTGAGCGCAGATGAAATTGAT<br>GTTGTTGAAGCACATGGCACCGGCACCCGTCTGGGTGATCCGATTGAAGC<br>ACAGGCCCTGATGGCAACCTATGGTCGTGGTCGTAGCGCACAGCGTCCGC<br>TGTGGCTGGGTAGCCTGAAAAGCAATATTGGTCATACCCAGGCAGCAGCC<br>GGTGTTGGTGGTGTGATTAAAATGGTTCAGGCACTGCGTCATGGTGTTCTG<br>CCTCGTAGCCTGTATGCAGAACGTCCGAGCACCGAAGTTGATTGGAGCGC<br>AGGCACCGTTGCACTGCTGGCACGTGAACGTGCATGGGAACCTGTTGAAG<br>ATCGTCCGCGTCGTGCCGGTGTTAGCAGCTTTGGTATGAGCGGCACCAATG<br>CACATGTTATTGTTGAAGAATTTGTTCCGGAACCGGCAGGCGAAAGCGAA<br>GCAGAACCGAGCGCACCGTGGGCAGAAAATAGCGTTCCGCTGGTTCTGAG<br>CGGTCGTACACCGGAAGCAGTTCGTGCACAGGCAGCAGCACTGCGTGATC<br>ATCTGGCAGCACGTCCGGAACTGAGCCTGGCACGTGCAGCACGTGAAAGC<br>GCAGTTCATCGTACCGCATTTGAACATCGTGCAACCGTTACAGGTGATCGT

		GATGCAGTTCTGGCAGGCCTGGGTCATGTTACTCCGGTTGCAGCCGGTGGT<br>GGTCGCGTTGCAGCAGTTTTTAGCGGTCAGGGTGCACAGCATGTTGGTATG<br>GGTCGTCAGCTGGCAGCAGATTTTCCGGTTTTTGCCGGTGTTCTGGATGAA<br>GTTTGTGCAGTTGTTGATCCGCTGCTGGGTCGTAGCCTGCGTGAAGTTATG<br>TGGTCAGGTAGCGCAGAACAGCTGGAACGTACCGAATTTGCACAGCCTGC<br>ACTGTTTGCATTTGAAGTTGCACTGGCACGTCTGTGGCAGAGCTGGGGTGT<br>TGAATTTAGCGTTCTGGCAGGTCATAGCGTTGGTGAAATTGCAGCAGCAG<br>TTGTTGCCGGTGCACTGAGCCTGGATGATGCAGCCCGTCTGGCAGTTGTGC<br>GTGGTCGTCTGATGCAGCGTCTGCCGGAAGGTGGTGCAATGCTGGCAGTT<br>GCAGCGGGTGAAGAAGAAGTTGCAGCAACCCTGGGCGATCCGGCACTGGT<br>TGGTATTGCAGCAGTTAATGGTCCGGAAGCCGTTGTTGTTAGCGGTGCACG<br>TGCCGAAGTTGAACGTGTTGCCGGTGTTTGGCGTGAACGTGGTCGTCGTAC<br>CAGTCGTCTGCGTGTTAGCCATGCATTTCATAGTCCGCTGATGGAACCTGT<br>GCTGGATGAATTTCGTGCCGTTGCAGATGAACTGGATCGTCGTGAACCGA<br>CCCTGCCGGTTATGGCAAGCGCAGAAACCACCCATCCGTTTGCAACCGCA<br>GCATATTGGGTTGATCATGCACGTCGTGCAGTTCGTTTTCATGATGCAGTT<br>ACCCGTCTGGCCGAAGCAGATGTTGTTGTTGAAATTGGTCCGGATGCGGTT<br>CTGGCACCGCTGATTGATACAGGTCGTACCGTTCTGAGCAGCTGTCGTCGT<br>GAACAGAGCGAAACCCTGACCCTGCTGACCGCACTGGGTGAAGCACATGC<br>ACATGGTGTTGAAGTGGATTGGACCACCCTGTTTCCGGCAGCACCGCGTG<br>CCGATCTGCCTGCATATCCGTTTCAGCATCGTCGTTATTGGCTGGCACCG
	**DH-KR 1**	CCTGCACTGACAGGTGCCGGTGCAGATGCCCTGGACCATCCGCTGCTGAG<br>CAGCCTGGTGGAACTGCCTGGTCAGGGTGGTGTTGTTCTGACAGGTCGTAT<br>TAGTCCGGATCGTGATCCGTGGCTGGCAGATCATGCAGTTAGCGGTGCCG<br>TTCTGTTTCCGGGTACAGGTTTTCTGGAACTGGCACTGCGTGCCGCACGTC<br>AGGCAGGTTGGCGTCAGGTTGCCGATCTGGTTGTTCAGGCACCGCTGGTG<br>CTGCCAGCAGCGGGTGCAGATATTCAGGTTTGGGTTGAACCGCCTGGTGA<br>AGCAGAACGTGCACTGGTGGTTCGTGGTCGTTCAGGTGATGGTGATTGGG<br>TTGAATATGCAACAGGTCGTCTGGTTGAAGAACCGCCTGAAACCAGTCCG<br>CAGGGTAGCCCTCCGACCGATGCAGAATGGGCAACAGGTCAGTGGCCTCC<br>GCAGGGTGCAGAAGAAATGCCGGTTGAAGAACTGTATGATGCACTGGCAG<br>CCCGTGGTTATGGTTATGGTCCGGTTTTTCGTGGTCTGCGTGCACTGTGGC<br>GTAGCGGTGATGAACTGTATGCCGAAGTTGTTCTGCCGGAACGTGCACAG<br>GATGGTCGTTATGATTGTCATCCGGCACTGCTGGATGCAGCACTGCATCCG<br>CTGGCAGCAGCAGCAGATAGTGGTCAGCAGGTTCGTCTGCCGTTTGCATTT<br>GGTCGTGCCACCGTTCATAGTCCGGGTGCAAGCGAACTGCGTGTTCGTCTG<br>CGTACGGGTGCAGAAACCATGCGTCTGGATGCAGTTAGCCCGACAGGTGA<br>ACCTGTTCTGACCATTGGTGAACTGGTTCTGCGTGCAGCAGATACCGCACG<br>TCTGACAGCCCGTGCCGAAGGTCATGTTCATCGTCTGGGTTATGAAGTTAC<br>CTGGCAGCTGCTGCCTGATCCGCCTCGTGCAGATCGTCTGCCTGGCACCTG<br>GATTGTTCTGGCACCTCCGGCAACCGATGTTAGCTGGACCCGTGATCTGGC<br>AGAACATACCCTGGCAGTTGATCTGGATACCACACCGGATCGTCCGGGTC<br>TGGTTGCACGTCTGGCAGATGTTACCCATGTTCCGACCGCAACCGCACCGG<br>ATGGCGTTCTGCTGTTTGCAGCACATCCTGATCTGCTGGTTACCGCACTGC<br>AAGCACTGACCGATGCCGGTGTTGATGCTCCGGTTTGGTGTCTGACCCATG<br>AAAGTGATGATGAGCTGGATACCGCAGCAGTTTGGGGTGCAGGTCGTGCA<br>GCAGCCCTGGAACTGCCGGATCGTTGGGGTGGTCTGGTTGATCTGCCTCCG<br>GCAGGCGTTCGTCCGGATCTGGGTTGTCTGGCTGGTCTGCTGAGTACCGAT<br>ACAGGTGAAGATCATCTGCGTCTGCGTCCGGATGGTGTTAGCGTTCGTCGT<br>CTGATTAAACCTGATCGTCCGACCCAGCCTCCGGCAGATTGGACCCCGAG<br>CGGCACCGTTCTGATTACAGGTGGTACAGGTGCACTGGGTGGTCATGTTGC<br>ACGTTGGGTTGCAGGTCTGGGTGGTTGTAGCCTGCTGCTGGTTAGCCGTCG<br>TGGTCCTGAAGCACCGGGTGCACAAGAACTGCTGGCAGAACTGGCAGCAA<br>CCGGCACTCCGGTTCGTATTGTTGCAGCAGATGTTGCAGATCGTAGCGCAA<br>TGGCAGGTCTGGTTCTGGAAGCAGCAGATGCAGGCACCCCTGTTCGTGCA<br>GTTTTTCATGCAGCGGGTGTTGCCGATGAAACACCTCTGCTGGAAACAACC<br>CTGGGTCGTTTTCATGCCGTTCTGGAAGGTAAAGCCGGTGGTGCACGTGTG<br>CTGGACGAAGTTCTGGGTGAAACCGATCTGGATGCATTTGTTGTTTTTAGC<br>AGCGTTAGCGGTGTTTGGGGTGGTGCAGGTCAGAGCGCCTATGGTGCAGG<br>TAATGCAGTGCTGGATGCCCTGGCAAGCCGTCGTCGTGCACGTGGTCTGG<br>CAGGCACCGCACTGGCATGGGGTCCGTGGTCAGGTGGTGGTATGGTTGAT

		GATGATCTGGAACGTCGTCTGCGTCGTCAGGGTCTGAATCCGCTGCCGGTT<br>GATGATGCCATTGCAGCACTGAGCACCGCAGTTCCGCTGGGTGAAAATCG<br>TGTTCTGGCGGATGTTGCATGGTCACGTTTTCTGCCGCTGTTTGCCGCAAC<br>CCGTCCGGCACCGCTGTTTACAGCACTGCCTCCGACACCGCGTCATACCGC<br>AAGCGCAGCAACAGCAGCACCGGGTCCGACCGCAGAA
	**ACP 1**	CGTCTGGCAGCACTGCCTGCCGCAGATCGTGCAAAAGCACTGAGCGATCT<br>GGTTCGTGCAGAAATTGCAAGCGCAGTGGGTCATCATACCATGAGCGCAG<br>TTGATGCCGAACGTCCGCTGGGCGAATTTGGTTTTGATAGCCTGATGAGCG<br>TTCAGCTGCGTAATCGTCTGAGTGCAGCAACAGGTGTGCGTCTGCCTGCAA<br>CCCTGGTTTTTGATCATCCGACCGCAGGCGCACTGGCACGCCATCTGGAAA<br>GCGAACTGGCAACCGAACCGAGCACCCGTCCGCGTCAGCCTGCC
	**KS-AT 2**	ACCGCAGAAACCGCAGTTGCGGATGAACCGCTGGCGATTGTGGGTATGGC<br>CTGTCGCTATCCTGGTGGCGTTGCAAGCCCTGAGGACCTGTGGCGTCTGGT<br>GGCACAGGGTCGTGATGCCGTTACCGCCTTTCCGACCGATCGCGGATGGG<br>ACCTGGCTGGCCTGTATCATCCGGATCGTAGCCGTGCAGGCACCACCTATG<br>CAAGCGAAGGTGGCTTTCTGGATGATCCGGCAGGTTTTGATGCCGATTTCT<br>TCGGTATTTCACCGCGTGAGGCCCTGGCTATGGACCCTCAACAACGCCTGC<br>TGCTGGAAGCAAGTTGGGCAGCAGTTGAACATGCAGGTATTGCACCGGAT<br>ACCCTGCGTGGCTCACGTACGGGTGTGTTTGTTGGCACCATGTATAATGAT<br>TATTTCAGCCGTCTGAGCAGCACACCGGAAAGCCTGGAAGGTATTATTGG<br>TATTGCAAATAGCAATAGCGTTATGAGCGGTCGCATTAGCTATCTGCTGGG<br>TCTGCAAGGTCCTGCCGTGACCCTGGATACCGCCTGTAGCTCAAGCCTGGT<br>GGCACTGCACCTGGCAGGTCAGTCACTGCGTCGTGGCGAATGCGACCTGG<br>CCCTGGCTGGGGGTGCCACCGTTATGGCAAGTCCGCATATTTTTGTGGAAT<br>TTAGTCGCCAGGGTGGTCTGGCCGTTGATGGTCGTTGCAAAAGTTTTAGCG<br>CAGATGCAGATGGCACCGGCTGGTCAGAAGGTGTGGGTCTGCTGGTGCTG<br>GAACGTCTGTCAGATGCCCGTCGCCTGGGCCACGAAGTGCTGGCGGTTGT<br>TCATGGTTCAGCCGTGAACCAGGATGGTGCCTCAAATGGCCTGACCGCAC<br>CGTCAGGTCCTGCCCAGCAGCGCGTTGTGGAAGCAGCACTGGCACAGGCA<br>GGTATTGCCGCAACCGAAGTGGATGCAGTTGAAGCGCATGGTACAGGTAC<br>ACGCCTGGGCGACCCGATCGAAGCCCAAGCACTGATTGCCACGTATGGTC<br>GTGAACGTGAAGCCGAACGTCCTCTGTATCTGGGTAGTCTGAAATCAAAT<br>ATTGGCCACGCGCAGGCAGCCGCAGGCGTGGGTGGCGTTATCAAAATGGT<br>GCAAGCCCTGCGCCATGAACTGCTGCCTCGTACCCTGCACGCAGATACCC<br>CGAGTCCGGAAGTAGATTGGAGCAGCGAAGCCGTTCGTCTGCTGACCGAA<br>GAACGTCCGTGGCCTCGTGGTGATCGTCCTCGTCTGGCAGGCGTTAGCTCA<br>TTTGGTATTAGCGGTACAAACGCCCATGTTGTTCTGGCTGAAGGTGATCCG<br>CTGGATGGTGAACCTGCCGAAGGTGCTCCGGTTAATGGTGAAAGCGTTGA<br>AAGCGGCACCCCGATGGATACCAGCGATAGCGCAGCAGCCCGTGCACCGC<br>TGCCGTATGTTCTGACCGCACGTAGCGCAGCAGGTCTGCCTGCACAGGCA<br>CGTGCACTGCATAGCCATCTGATTGGTCGTCCTGGTCTGGAACCGGCAGA<br>AGTTGCACGTAGCCTGGTTACCACCCGTAGCCTGCATGATCATCGTGCGGT<br>TGTTGTTGCAGAAGATCGTCAAGAGCTGCTGGACGCACTGGCAGCGCTGG<br>CAGATCCGGCAACCACCACACCGCGTCGCACCGTTAGTACCGGCACCGCA<br>GGTCGTGGCCGTGTTGCGGGTCTGTTTTCAGGTCAGGGTGCCCAACGTCCG<br>GGTATGGGTCGCGAACTGGCAGGTCGTTTTCCGGTGTTTGCAGGCGTGCTG<br>GACGAGGTTTGTGCCGTGGTGGACCCTCTGCTGGGACGTTCACTGCGCGA<br>GGTGATGTGGTCAGCACCGGGTGAAGTTCTGGAACGCACAGAGTTTGCCC<br>AGCCAGCCCTGTTTGCCTTCGAGGTGGCTCTGGCTCGCCTGTGGCAGTCAT<br>GGGGAGTGGAATTTTCAGTGCTGGCAGGCCATTCAGTGGGTGAAATCGCA<br>GCCGCATGTGTTAGCGGTGTGCTGAGCCTGCCGGATGCCGCACGTCTGGTT<br>GTGGCACGTGGACGCCTGATGCAGGCACTGCCTGAAGGCGGAGCAATGGT<br>TGCGATTGCAGCAGGCGAAGATGAAGTTAGCGCCAGCCTGGCCGATGTTC<br>CGGATGTTGCCATTGCCGCTGTGAATGGTCCAGAAGCAGTGGTAGTTTCA<br>GGCACCGAAGCCGATGTTCTGCGTAGCGCAGATCATTGGCGTGAACAGGG<br>ACGTCGCACCTCACGTCTGCGTGTGTCACATGCCTTTCATTCACCTCTGAT<br>GGAACCAATGCTGGACGATTTTGCAGCAGTTCTGACCGAACTGACCTTTCA<br>TGAACCGGCACTGCCGATTAGCCCGAGCGCAGATAGCAGCCGTAGCTTTG<br>CCAGCGCAGAATATTGGCTGGACCATGCCCGTCATGCCGTGCGTTTTGCAG<br>ATGCACTGAGTGGTCTGGATGGTGCGGATGTTCTGGTTGAACTGGGTCCTG<br>ATGCAGCCCTGGCACCTCTGGCAGGTACAGATAAACCTGTTCTGGTTTGTG

		CACGTCGTAATCAGCCGGAAGTTCGTACCCTGGTTACAGCACTGGGTGCC GGTCATGCGCATGGTGTGGCGGTTGATTGGACAGCAGTGCTGGGTGAAGG TCGTCAGGTGCGTCTGCCGACCTATAGCTTTCAGCATGAACGCTATTGGCT GGATGAAGATACCGCAGCCGGTCGTGGTACTGGTGCA
	**KR 2**	GCACATGATGCAGCGCAGGCACGTTTTTGGCAGGTTGTTGAAGATCAGGA TCTGGATGGTCTGACCCGTACCCTGGGTCTGGATGCCGAAACCAGCCTGC GTGCAGCCCTGCCGGTTCTGCATGATTGGCATCGTACCAGCAGCACCCTGG CGCAGGCAGCGGGTTGGCGCTATCGTGTTGCATGGGATCGTGTTCCGACC GATGATAGCGCAGTTGCACTGGATGGCACCTGGTGGATTGTTGTTCCGAGT GATGCAGCAGATGCCACCACCGCAGATGCAGTTCGTCGTGCCCTGGCAGC GGCAGGCGCAAATCCGCGTATTCTGACGATTGATCCGCATCGTACCGATC GTGCAGCACTGGCCAAAGAGCTGGCAGCCGCAGCCGATGGTACAGTTGCA GGTACAGTTAGCCTGCTGGCCGAAAGCGGTGGTGAAGATACAGGTCATCG TGGTGTGGCAGCCGGTGCCCTGGCCACCCTGGTTCTGCTGCAAGCGCTGCA TGATGCAGATATTACCACCCGTCTGTGGACCCTGACCCGTGGTGCAGTTCG TACAGGTCCGGCAGATACAGCTCCGGGTCCGTGGCACGCACAGGTTTGGG GTCTGGGTCGTGTTGCCGCACTGGAACATCCTGCACTGTGGGGTGGCCTGA TCGATCTGCCAGCAGAAGGTGAACCGGCAGGTCTGGCAGCCGTTCTGAGT GGTACAGCCGGTGAAGATCAGGTTGCACTGCGTGAAGATGGTGTTCGTGC CCGTCGTCTGACACCGGCAGATACCCATGATGCACCGGGTGCCGATGCAC AGGCACCGGGTGCTGATCGTTGGACCGATGGTGCAGTTCTGATCACGGGT GGCACGGGTGCCCTGGGTGCACATACCGCACGTATGCTGGCCAGCCGTGG TGCAAGCCGTCTGGTGCTGGTTTCACGTCGTGGTGCAGCCGCACCGGGTGC GGAAGCACTGCGTGCGGAACTGGAAACCCTGGGTGCAACCGTTGATCTGA CCGCATGTGATGTTACCGATCGTGATGCGGTTGGTCGTCTGGCAGAAGCCC TGGCAGCCGAAGGTACACCTGTTCGTGCCGTGGTTCATGCCGCAGGCGTT GCAGCGGAACGTCCACTGACAGAACTGGTTGGTGATGATTTTTCAGCAGT TAGTGATGCAAAAGTTACCGCAGCAGAAATTCTGGATGATGTGCTGGGTG ATGACCTGGCAGCCTTTGTTGTGTATAGCAGCATTGCAGGCACCTGGGGTT CAGCCGGTAATGGTCCGTATGCAGCCGCAAATGCACATCTGGATGCACTG GTTGAACGTCGCCGTGCCCGTGGTGCCGTTGGCACCGCTATTGCCTGGGGT CCTTGGAGCGGTGGCGGTATGGCAGATGATCGTTTTCAAGAAGAAATGCT GCGTCGCGGTGTTAGCGCACTGTCACCGCAGGGTGCCACCGCAGCACTGG CGCAAGCCCTGGAACATGATGATACCACCGTTACCGTTGTTGATGTTGATT GGGATCGTTTTGCACGTGTTTTTGCATGTAATCGTCCGAGTCCGCTGCTGC GTCATCTGACCGAACCTGCCGCAGCTGCCGGTGAAGCACCGGCACGTACC GAACTGGCAGAG
	**ACP 2**	CGGCTGGCAGCCCTGGATGCAGGTACACGTCGTGGCGCAGTTCTGGATCT GGTGCGTGCGGAAGTTGCGGGTGTTCTGGGTCATGCAACCAGCCAGGCAG TTGCAGTTGATCGTGCATTTACCGATATGGGTTTTGATTCACTGATGGCCG TTGAACTGCGTGCACGTCTGGGTGCCGTTACAGGTCTGGCACTGCCGACCA CCCTGGTGTTCGATCGTCCGAGCCCTGCCGAAGTTGCAGGTTTTCTGTGTG ATCGTTTTGAA**CCAGATCGTGATGCCCTGACACATGAAATTCTG**
	**linker**	**GAAAAACTGGATTGGCTGGAAAAAACGCTGCTGGATGCTGCGCATAG CCAGGGTGCCCGTGCACGTTTTGGTAGCCGTCTGGATGCGCTGCTGG CACGCCTGGATCGTACCACCGTTCCGCCTGAAAGTCGTCTGAGCGAT CCGGATACCGATACCGATGCCATTGAAAGCGCAACCGCAGAGGAACT GCTGGCCTTTGTTGAAAAACACTTTGATTCA**
**PnC (Accession number AFJ05066)**	**KS-AT**	ATGGTTATGACAGGCGAAGGTCGTAGCGATCAGCTGGTGACCGCACTGCG CAAGGTAACTCGTGACCTGCGTGACACCCAACGTCGCCTGCAAGTTGAAG AGGACCGTAACGGTGAGCCAATCGCTGTCGTTGGTATGAGCTGCCGTTTCC CGGGTCACGTCGACTCTCCGGAAGCACTGTGGGAACTGGTTTCTTCCGGCC GTGGTGCTTCTGGTTCTTTCCCGGTTGATCGTGGTTGGGACCTGGACGCTC TGTTCTCCGAATCTGCTGACGGTAAAGTTCCGTCCTACGTTCGTGAAGGTG GCTTCCTGTATGATGCTGGTTGGTTCGACGCTTCTTTCTTCGGCATTTCTCC GCGTGAGGCGGTAACTATCGACCCGCAGCAACGTCTGCTGCTGGAAGTTG CTTGGGAATCCCTGGAACGTGCACGCATCAGCCCGCAGGCACTGCGTGGT TCTGACGTAGGTGTTTTCGCTGGTGCAATGAACCAGGACTACGCAGTTCGC CTGCATGAAAGCATCGAAGATTTCGAAGGTTTCCTGACCACCGGCAACAC CGGCTCTGTAGTTTCCGGTCGTCTGAGCTATACGCTGGGTCTGGTAGGCCC GGCTGTTACCGTAGACACTGCTTGTTCTTCCTCTCTGGTAACCGTGCACAT

		GGCAGCACGCTCTCTGCGCTCTCGTGAATGCTCTCTGGCCCTCGCAGGCGG<br>CGTAACCGTAATGTCTATGCCGACCACCTTTACTGAGTTCTGCCGTCAGCG<br>CAACCTGGCGCCGGACGGTCGCGCTAAGTCCTTCGCAGCAGCAGCTGATG<br>GTACTGCTTGGGCTGAAGGCGCAGGTATGCTGGTGCTGGAACGTTTAAGC<br>GACGCTGAGCGTAACGGTCATCCGGTGCTGGCTCTGATCCGTGGGTCTGCT<br>GTAAACCAGGACGGTGCTTCTAACGGCCTGTCTGCTCCGAACGGCCCGTCT<br>CAAGAACGTGTGATCTGGCAGGCCCTGCGCGATGCTCGTCTGACTGCTGA<br>CGAGATCGATGCAGTTGAAGCTCACGCAACCGGGACTCGTCTGGGTGACC<br>CGATCGAAGCTCAAGCACTGCTGGCAACCTACGGTCAGGGCCGTTCTGCT<br>GACCGTCCGCTGTGGCTGGGTTCTCTGAAATCCAACATCGGTCACGCACA<br>GGCAGCTGCTGGTGTGGGCGGTGTTATCAAGATGGTAATGGCTTTACGCC<br>ACGGCGTTCTGCCGCAGACCCTGCACGTGGACGCGCCGACCCCGGAAGTT<br>GATTGGTCTGCTGGTGAGGTTCGCCTGCTGACGGAGCGTCGTGAATGGCC<br>GCGTGCAGGCCGCCCGCGTCGTGCTGGCGTATCCTCTTTTGGTGTGTCCGG<br>TACTAACGCTCACCTGATCCTGGAAGAGGCGCCGGCAGCTCCGCCTCGTG<br>ACGGTGCAGCTGACCGTGCTGACGATGGTGGTGGCGCTACCCTGCCGTGG<br>GTTCTCTCTGCACGTACCGCAGCTGCTGTGCGTGAGCAGGCTCGCCGTCTT<br>CATGGTCACCTGACTGACCATCCGGAGCTTGTTCCGGCACAGGTTGCTCGT<br>TCTCTGGTAACCACTCGTTCCACCTTCGAACAGCGCGCAGTTGTTCTGGGT<br>ACTGACCGCGCTGAGCTGCTGGACGGTCTGGATTCTATCGTTCAGGGTGCC<br>CCGGACGCACGTACTGTTACCGGCTCCGCTGTTGGTGGTCGTGAGGTAGTA<br>TTCGTGTTCCCAGGGCAGGGTGGTCAGTGGGCTGGCATGGCACTGGAACT<br>GATGGAAGAATTTCCGGTTTTCGCTGAAACCCTGCACTCTTGCGCTGACGC<br>CCTGGCTGACTACGTTGACTGGTCTCTGCTGGACGTACTGCGCGAGGCAG<br>AGGGTGCTCCGGGTCTGGACCGTGTTGACGTCATCCAGCCGGTTCTGTTCT<br>CTGTAACCGTTGCACTGGCAGACCTGTGGCGTAGCCTGGGCATCGAGCCG<br>TCTGCTGTCGTTGGTTCTAGTCTGGGCGAGATCGCAGCTGCATACACTGCT<br>GGTACTCTGAGTCTGGCAGACGCGACCAAAGTGGCCGTTCTGCGCAGCCG<br>TGCACTGCTGGAACTGTCTGGTCGTTCTGGCATGGTTTCCGTGCCGCTGGG<br>TCGTGCTCAGGTAGAAGAACTGATCGCAGCTTGGCCGGATCGCCTGTTCGT<br>GAGCGGTGTTAACTCTCCATCTATCTCCGTAGTTTCTGGCGACAACGAGGC<br>AGTGGACGAACTGCTGGCGGTTTGTGCTGAGCGTGGTGTTCGTGCACGTC<br>GTGTTGCTACCGACTGTGCTTCTCACTACCCGGCTGTAGAAGCTCTGGAAG<br>AGCGTCTGCTGACGGACCTGGCAGACCTGGCTCCGACTCCGGGTCGTATC<br>GCTTTCCTGTCCACCGTTTCTGGTGAAAGCGCAGAAACTGAACCAGTACCG<br>GATGCTGCTTACTGGTATCGTAACACTCGCCGCACCGTTGAGTTCGAGCCG<br>GTTATTCAGCGTCTGGCAGCGACTGGTTCCAAAGTTTACATCGAAATCTCT<br>CCACATCCGGTGCTGCAAGTTGCTCTGAGCGAGATCGTGGAAGGTGAATC<br>TCGTGAAGCTGCGGTTCTGAGCACTCTGCGTCGTAACACTTCTGACCGCCG<br>CGCTTTCCTGACCAGCCTGGCGAAAGCATACGTTTCTGGTGTAACTGTGGA<br>CTGGGCCGCACTGCCGTCCCTGGCGGGCGCAGCACAGGTGGACCTGCCGA<br>CTTATGCATTCCAACGTGAGCGCTACTGGCCGCGCCCGGCAGCTGCAAGT<br>AACGGCGGTCACGGT
	**KR**	GCTGGTACAGGTGCTCGTGCTGGTGTTGGCCACGGTACTGTTGACGCCCAC<br>TTCTGGGAAGCTGTAGAGAACGGCGACCTGGGTAGCCTGGGTCCAGACGT<br>ACGCTTTGACGACGAAACCCCGCTGAAGGAGGCACTGCCGGAACTGGCTT<br>CTTGGCACCGTCACGGTCTTGAACAGGCACGCGTTGACGGTTGGCGTTAC<br>GTTGAACGTTGGCGTCCACTGGACGTTCCGGCTACTGGTCCACACGGCAA<br>ATGGCTGCTGGTGCACCCGGGTGGTACTGAATCCGATGCGACGACGGAAT<br>GGGTACGCGAATCTCTGCTCGAGGCTGGTTGTTCTGTAGTGGACCTGCTGG<br>TTGACACTACTGACACTGACCCGGCAGCTGTGACGGAGCGTCTGAACCAG<br>GCTTGCGCTGGTGGTGGCCCGGAGCCGGTTGGTGTGGTTAGTCTGCTGGCG<br>TTCGACGGCCGCCACGACACTGCTCGCCCGTCCGTACCGCGTGGTACAGC<br>AGCTACTCTGGCTCTGGTACGTGCACTGGGTGCAGCGGGTATCACTGCTCC<br>GCTGTGGTGCCTGACCCAGGGTGCAGTTACAACTGGCACGGGTGACCGTC<br>TGGACGCTGTAGAACAGGCACAAATCTGGGGTCTGGGCCGTGTGGTTGCA<br>CTGGAACACCCGGATCGTTGGGGCGGTCTGGTTGACCTGCCGTCTACACTG<br>GACCCGCACGTACGTACTCAGCTGTGTGCAGCACTGGGCGGTGCTCATAC<br>CGACGAGGACCAGCTGGCCCTGCGTCCGGTGGGCATCCTGGGTCGCCGTC<br>TGGTTCCGGATGACGGTGACCGCCAGGCAGCTCGTACTGCACCGTGGCGT<br>CCAGCTGGTACTGTTCTGATCACAGGTGGTACTGGTGCTCTGGGCGCTCAC

		GTTGCGCGCTGGGCCGCTGCTCAGGGTGTAGGTCACCTGCTGCTGGCTGGT<br>CGTCGCGGTGCGGATGCACCGGGCGTTGCAGAACTGCGTGCAGACCTGGA<br>GGCATCTGGTGCAACTGTAACCGTAGCTTCTTGCGATGTGGCAGACCGCC<br>GTGCTGTTCAGGAACTGCTGGATGGTATCCCGGATCGCCACCCGCTGACC<br>GCCGTGTTCCATGCAGCTGGCGTCCTGGACGATGGTATGACGGCTGATCTG<br>GACACCGCACGTCTGGAGACCGTACTGGCCCCTAAAACCGAAGGTGCTCG<br>TCTGCTGGACGAACTCACTCGTGATACCGAACTGAGCGCATTCGTACTGTT<br>CTCTGGTTTCGCAGCAACCGTGGGTTCTAGTGGTCAGGCCGGCTACGCGGC<br>AGCTAACGCTCACCTGGACGCACTCGCGCAGCGCCGTCGTGCTGACGGTC<br>TGCCGGCAACTTCTATTGCGTGGGTTCCGTGGACTGGCGGCGGTCTGGTTG<br>ACGAAGGTATCGAAGAGCGCCTGCTGCGTCGTGGTCTGAGTGCTATGGAA<br>CCGCGCCTGGCAGTGCTGGGTCTGTCTGCCCTGCTGAGCGGTGGCCCGGAT<br>GGTCCGGCTAACGTAGCACTGGTAGACGTTGATTGGCGTCGTTTCCTGCCG<br>TCTTTCACCGCTTCCCGCCCGTCTCCGCTGCTGCGTGAGCTGCACCAGCCG<br>TCTCCGGCGGACCCGGGTGCTACTGCTGGTCGTGACGGCACCCCAGGCGA<br>TGGCGCAGGTCGTACTCTGGCAGAACGT
	**ACP**	CTGCCGGGTCTGACCGACGCTGAACGTGACCGCCTGCTGCTTACAACTGTT<br>CGCGCACAGGTGGCTTCCACTCTGGGTTATCCGGGCCCGGATGCTGTACCG<br>CCGCGTCAGCAGTTCAAAGAACTGGGTATCGACTCTCTGACTGCATTAGA<br>ACTGCGCAACGGTCTGCAAGCTGTTGCGGGCGTAGCACTGCCGGCTACTC<br>TGGTTTTCGATCATCCGACCCCGGAGGCAGTTGCTGGTCACCTGTCCCGTA<br>CTCTGCTGGGTGATGGTGCACACACC
	**linker**	**AGCTCTTCCCTGTTTACCGAAATCGACCGCCTGGGCTCCGCACTGGGT<br>TCCGCTGACCTGGATGAAGCAGAACGTAACCTGGTAGCAACTCGTCT<br>GCGTTCCCTGGCTTCTCGCTGGGATGCTGGTCCGTCTTCTGGCGCTG<br>GTGACGTTGCACAGACTCTGGCTGACGCAGACCGCGCGGAGGTACTG<br>GCATTCATCGATCGTGAACTGGGCATCTCTTCA**
**PnD (Accession number AFJ05067)**	**KS-AT**	ATGCGTTCCGAAGAACAGCTGCTGGACTACCTGAAACGTGTAACTGCTGA<br>CCTGCACGACACCCGTGTGCGTCTGCAAGCTACCGAACAGGCCTCTCGCG<br>AACCAATCGCAATCGTTGGCATGGGTTGTCGTTACCCAGGCGGTGTTTTCT<br>CTCCGGAGGACCTGTGGCAGCTGGTTGCAGAAGGCCGCGACGTAATCGGT<br>GACCTGCCGACTGATCGTTCTTGGAACCTGGACACTCTGTACGATCCGGAC<br>CCGGCTGCACCGGGCACGACTTACGCTCGTGGTGGTGGTTTCGTTGCTACC<br>GCGACCCACTTCGACGCAGGCTTCTTCGGCATCTCCCCTCGTGAAGCAGCT<br>GCAATGGACCCGCAGCAGCGTCTGGTACTGGAGACTGGTTGGGAAGCTCT<br>GGAGCGCGCGGGCATCCCGCCGACCTCTCTGGGTGGTTCTGATACTGGCG<br>TTTACATCGGTACTGGTATGCAAGACCACATCATCCACCTGCAAAAAGCTC<br>GCCAGGAGGCTGAAGGTTTCGTAGGCACGGGTAACGCGATCTCTGTGGTT<br>TCTGGTCGTCTGGCTTACACCCTGGGTCTGGAAGGCCCGGCTGTTTCCGTA<br>GACACGGCCTGCTCTTCTTCTCTGGTTGCACTGCACCTGGCTGTTCAGGCT<br>CTGCGTGCTGGTGAATGCTCCCTGGCTCTGGCAGGGGGTGCTACGGTGATC<br>AACACTCCGGTAATGTTCGTTGAGTTCTCTCGTCAGCGCGGTCTGTCCCCG<br>GACGGTCGTTGCCGCTCTTTCGCTGCTGATGCTGATGGCACGGGTTGGAGT<br>GAAGGCGCGGGCGTACTGGTTCTGGAGAAACTGAGCGACGCTCGCCGTCA<br>CGGTCATCAGGTTCTGGCAGTTGTTCGTGGTTCCGCTGTTAACCAGGACGG<br>TGCTTCTAACGGTCTGACTGCTCCGAACGGCCCGTCTCAGCAGCGCGTTAT<br>CCGTCGTGCTCTTACCGCAGCTGGCCTGACCGCAGATGAAGTCGACGTAG<br>TAGAGGCACATGGTACTGGCACCCGTCTCGGGGACCCGATCGAAGCTCAG<br>GCTCTGCTTGCAACCTACGGCCAGGGCCGTTCCGCTGACCGTCCGCTGTGG<br>CTGGGCTCTCTGAAGTCTAACATCGGTCACGCTCAGGCAGCAGCTGGCGT<br>GGGTGGTGTTATCAAATCTGTAATGGCACTGCGTCACGGTGTTCTCCCGCA<br>GACTCTGCACGTTCACGCACCAACCCCGGAGGTAGACTGGTCTGCTGGCG<br>AAGTGCGCCTGCTTACCGAGCGTCGTGACTGGCCACGTGCTGGTCGTCCGC<br>GTCGTGCAGGTGTATCCAGCTTCGGTATCTCTGGCACTAACGCTCACCTGA<br>TCCTGGAGGAAGCACCAGATGAAGCGCCGGAAGAAGCTCCAGACGACGC<br>AACCGACCGCGCACCGCGTTTGCGTGGTATGCCGTGGGTCGTGAGCGGTC<br>GTGGTCGTCAGGCTCTGCGTGCGCAGGCTGCGCGTCTGCGTGACTGGGCA<br>GCTGGTCACCCGGATGTTGCACAGGAAGAAGTGGGTCTGGCTCTCGCGAC<br>CCGCCGTTCTGTATTCGAGCACACCGCTGTGATCACCGGCGCAGACCATG<br>ATGAACTGCTGAACGGTTTATCTGCTCTGTCTGAAGATCGCCCGGCACCGG<br>GTGTTCGTCTGGAGACAGCTGCTGGTGGTGGCCTCGCATTCGCGTTTACTG

		GTCAGGGTGCACAGCGTCCGGGTATGGGCCGTGGTCTGTATGAGACGTTC<br>CCGGCTTACGCGGAAGCATTCGACGAGGCTTGCGCTGCACTGGACCCGCA<br>CCTGGAACGCCCGCTGGCTTCCGTTGTGTTTGCAGACGGCCCGGCTGACGC<br>TGAGGCGCTGCAAACCACCGCTTATGCCCAACCGGCTCTGTTCGCAGTTGA<br>GACCGCTCTGTTCCACCTGATGCAGTCTATGGGTGTTCACCCGGACCTGCT<br>GATCGGCCACTCTGTTGGCGAACTGAGCGCTGCGCACGCAGCTGGCGTTC<br>TGAGCCTCCAAGATGCTGCACGCCTGGTAGCTGCTCGTGGTCGCCTCATGC<br>AATCTCTGCCGGAGGACGGTCGTATGCTGGCCATCCAGGCATCTGAAGAC<br>GAGGTTCTTCCGTCCGTAGCTGAACTGAGCGAGAAGGCTGGTGGCGTTGC<br>AGTTGCTGCTGTAAACGGCCCGGCATCTGTAGTTGTATCTGGTCGTACTGA<br>GGCTGTTCATACTCTCGAAAAAGAATTTGCAGGTCGCGGTCGTCGCGTAC<br>GTTACCTGGACGTGTCTCACGCATTCCACTCCCCACTGATGGACCCGGTTC<br>TGGACGAGTTCGCACGCATCGCGGCTTCTGTTACCTTCCGTCCGGCTACCA<br>CCCCGGTTATCTCTAACGTGACTGGTGACCTGATCGGTGATGACCGTCTGG<br>CTGACCCGTCCTACTGGGCCGATCACATCCGTGCTACCGTTCGTTTCGCAG<br>ACGGTGTGCGTGCTCTGGCACGTGAACAGGTTGACACCGTAGTTGAACTG<br>GGCCCGGACGCTGCACTGACCGCTCTTTGTGGTGAAATCCTGGACCAGGA<br>CACCGCAGCTTTCGTACCGACTCTGAGCCGTAAACACGACGAAACCTCTA<br>CTTTCCTGACTGCTATGGCTCGACTGCACGCTCGTGGCATTCCGGTTCGTT<br>GGCCGGCTGCGACCACTCCGTCTGTAGAAAGTGCTGATCTGCCGACCTAC<br>GCTTTCCAGCGTGAACGTCATTGGCTGGACGGTGTAGCGGAAACTGACGT<br>GGCCGGCACTGGTCTGACC
	**DH-KR**	GGCATCGGTCACCCGCTGCTCCCGGCTGAAACTGCACTGCCGGGTACTGA<br>GGGTGTTGTGCTGACTGGCTCTCTGAGTCTGCACGACCATGCTTGGCTGGC<br>AGATCACGCTGTTCTGGGCGTAGTACTGGTGCCGGGAGCAGGTCTGCTGG<br>ACATGGCGCTGACAGCAGCTGAACATGCAGGCTGCACCCAGGTTGAAGAG<br>CTGACCCTTGAATCCCCGCTGATTTTACCGGAGGTTGGTGCTCGTAGCGTA<br>CAGGTACTGGTAGGTGCGGCTCAGGACTCCGGGCAGCGTGCCATCACCAT<br>CCACTCCCGTCCGCAGGACGGTGACCCGCACGCAGCATGGACCCGCCACG<br>CCACCGGCCTGCTGGCTACTGGTCCACAGGAAGAACCGGCTACCTTCTCTG<br>AAGCATGGCCGCCGACTGGCGCTGTACCAGTTCCGGTTGACGACCTGTAC<br>CTCCGCCTGACTGAAGGTGGTGTGGATTATGGTCCTTCTTTTCGTGGTCTG<br>CGCGCAGCCTGGCGTCTGGGTGAAGACTTCTACGCAGACATCGACCTGCC<br>GCACCTGTCTGACGTAGAACGTTTCACCCTGCACCCGGCACTGTTAGACGC<br>GGCTCTGCATTCTCTGGCACTGCCGGGTGCTATCCTCCATACCGGCCAGGC<br>ACACCTGCCGTTCTCTTGGTCTGGTGTGCGTCTGCACGCCTCTGGTGCAGA<br>CGCCCTGCGTATCCGCGTTCGTGGCACGGGTTCTTCCTCAGTATCTCTGGA<br>ACTGGCAGACGGTACTGGCCGCCCGGTAGCAACTGTTGGCGAGCTGGCTC<br>TGCGTCCGGTATCTCAGGAACAACTGCGTACCCCGGGCGCAGATCCGACT<br>AGCCTGTACACCGTAGAGTGGCCGGTAAAGGAACTGCCGGAACGTGCTGA<br>AGGTTCTGCAACACGTGCGTGGGCAGTAATCGGTGCACCGGAGCCAGCTG<br>GTTACGCGGTTGAAGGTGTTGAGCTGTCTCACTATGCTTCTCCGGCTACCC<br>TGGCAGCAGCTCTGGATGCTACCGACGTGCCGGTTCCGGAGGCTGTTCTG<br>GTGCCGTGCTTCTCTCCACACTCCCCGGGTTGTCCACCGGGTGCATCTGCT<br>GGCCTGGTTGAGCGTGCGCACGACTATGCTGCAACTGTACTGGACCTGGT<br>ACGCACCTGGCTGGGTGATGCTCGTTTCGAGGCCTCTCGCCTGCTGCTGCT<br>CACCCGTGGCGCAGTTACCCCAGACGCAGGCCACCACCCGCTGTCTGACG<br>AGCTGGGCTCCCTGGCATGGGGTCTGGTCCGCACAGTACAGAACGAACAC<br>CCGGGTCGCGTCCTGGTAGCAGACCTGGATGAAGATCCGGCTTCTTGGTCT<br>GTTCTGCCCGGCGTGCTGGCACATGAGGAACCGCAGGTTGCTGTTCGTCGT<br>GGTGTAGCGCACGTACCACGCCTGACTGCTGCACGCGCAGCAGCGGAACG<br>TGCAACCCCGTTCGACTCCGTGGGCACCGTACTGGTTACTGGGGGTACTGG<br>TGGCCTGGGTTCTCTGCTGGCTCGCCACCTGGTAGTAGAGCACGGTGTACG<br>CCATCTGCTGCTGACTTCTCGTCGCGGCCCGCAGGCTCCGGGTGCAGCTGC<br>TCTGGCAGCTGAACTGACTGAACTGGGTGCACAGGTTACCGTTACCGCTTG<br>CGACATGGCTGACTCCCCGGCGGTTGAAGAACTGCTGGGTAGCCTGCCGG<br>CTGGCCACCCGCTGACCGCAGTAATCCACACCGCTGGTGTGCTGGATGAC<br>GGTCTGGTTCAAGACCTGACCCCGGAACGTCTGAACACCGTGCTGCGCCC<br>GAAAGCTGATGCGGCTGTTGTTCTTGACCGCCTGACCCGTCACCTGGACCT<br>GGCAGCTTTCGTCCTGTACTCTAGCGTAGGTGGCACCCTCGGTGGTCCGGG<br>CCAGGGCAACTACGCCGCAGCTAACGCATTCCTGGACGCACTTGCTCAGC

		GTCGCCGCGCCGAGGGCCTGCCGGGTCTGTCTCTGGGTTGGGGTCTTTGGT<br>CCGACACCACTGGTATGGCGGCTGAGATCGGTACTACCCATGTGGGCCGT<br>CTGAACCGTTCTGGTCTGGTTACCATGTCTCCGGCTGAAGGTCTGGCACTG<br>TTCGATGCTGCAATCTCCGGTGGCTATGGTCCGGTTGTCCTGCCTGTACGC<br>CTGGACCTGCCGGCGCTGAAGGCACGCGCTACTTCTGGCGCTCTGCCGTCC<br>GTTCTGGCAGACCTGGTACGTACCCCGGCGGGTCCGGCACGCGCGGCAAA<br>ATCTGGTACTGTGTCTGGTGCTGCTCTGCGTGCAGCT
	**ACP**	CTGTCTCAGCTGTCCGAAGACGAGCGCCGTCGTATGCTGCTCGACGTTGTA<br>CGTGAAAACGTTGCGGCTGTTTTGGGCCTGCGTCAGGACGGCGCTATGGA<br>CGAGGAGCAGCCGTTCAAAGACCTGGGCCTGGACTCTCTGACGGCAGTAG<br>AACTGCGCAACCGTCTGGCCAGCGCTACTGGCCTGCAACTGCCGGCAACC<br>CTGCTGTTCGACCTGCCTTCTCCGAGCGCTCTGGCTCACCACCTGCTGACT<br>CGCATGGGT**TCTCAAGAGAGTAAATCTCCGGTAGCTGAAGCAGTTGAC**<br>**CATCTGACCGCGCTGCTG**
	**linker**	**ACCACTCACGACGTAGGCGACCTGGAGCGTTCCCAGGTTACCGCCCG**<br>**TCTGCGTTCTCTGCTGTGGCGCCTGGACGACGGTGGCGTAGAGTCTA**<br>**CTGAAGACGCGGCCGACCAGGCAGAAACTGACGACGACATCTTCGCA**<br>**CTGGTTGACCGTGAACTGGGTCTGGCTTCA**
**DEBS thio-esterase (1MO2_A)**		AGCGGGACTCCCGCCCGGGAAGCGAGCAGCGCTCTTCGCGACGGCTACCG<br>GCAGGCGGGCGTGTCGGGCAGGGTCCGGTCCTACCTCGACCTGCTGGCGG<br>GGCTGTCGGACTTCCGCGAGCACTTCGACGGCTCCGACGGGTTCTCCCTCG<br>ATCTCGTGGACATGGCCGACGGTCCCGGAGAGGTCACGGTGATCTGCTGC<br>GCGGGAACGGCGGCGATCTCCGGTCCGCACGAGTTCACCCGGCTCGCCGG<br>GGCGCTGCGCGGAATCGCTCCGGTTCGGGCCGTGCCCCAGCCCGGCTACG<br>AGGAGGGCGAACCTCTGCCGTCGTCGATGGCGGCGGTGGCGGCGGTGCAG<br>GCCGATGCGGTCATCAGGACACAGGGGGACAAGCCGTTCGTGGTGGCCGG<br>TCACTCCGCGGGGGCACTGATGGCCTACGCGCTGGCGACCGAACTGCTCG<br>ATCGCGGGCACCCGCCACGCGGTGTCGTCCTGATCGACGTCTACCCGCCC<br>GGTCACCAGGACGCGATGAACGCCTGGCTGGAGGAGCTGACCGCCACGCT<br>GTTCGACCGCGAGACGGTGCGGATGGACGACACCAGGCTCACCGCCCTGG<br>GCGCCTACGACCGCCTCACCGGTCAGTGGCGACCCCGGGAAACCGGGCTG<br>CCGACGCTGCTGGTCAGCGCCGGCGAGCCGATGGGTCCGTGGCCCGACGA<br>CAGCTGGAAGCCGACGTGGCCCTTCGAGCACGACACCGTCGCCGTCCCCG<br>GCGACCACTTCACGATGGTGCAGGAACACGCCGACGCGATCGCGCGGCAC<br>ATCGACGCCTGGCTGGGCGGAGGGAATTCGAGCTCCGTCGAC
**Npt (OSY40 025) Codon optimize d**		ATGATTGAGAAGTTACTCCCGGCGCCAGTCAGAACGGCAGAGACTTTCGA<br>CGATGCGCCTTTATCTGAAATGTTCCCCGAAGAGTGGGCGCAGGTTGCAA<br>ACGCTGTACCCAAACGCCAACGTGAGTTCGGTACTGTACGAGGGTGCGCT<br>CGTCGTGCCCTGGCCGAGCTTGGCTTCGCTCCGGCACCATTGCTGCCTGGA<br>CCTCATCGTGAGCCGCAGTGGCCAGATGGGGTTGTGGGCGCGATGACGCA<br>CTGCGCGGGATATCGCGCTGTAGCGGTGGCACGCGCCGGCGAAGTTCGCA<br>CAATCGGCCTGGATGCCGAACCGAATCTCCCACTAAATGACCCGGGCGTT<br>CTTGACCTGGTGACATTACCGGAAGAACGGGACCAGATCCGGCGCCTCGC<br>CGCCCTTCAACCGGAAGTCTGTTGGGATCGCTTGGTCTTTTCCGCAAAAGA<br>AAGTGTCTACAAAGCCTGGTTTCCGCTGACGCGCCGTTGGTTGGATTTTGA<br>AGAAGCACTGCTGACCTTTGATCCGACCAACGCGACCTTTACCGCGCAGC<br>TGCTGGTGCCGGGCCCGGTGGTTGATGGTCGTGAACTGACCGAATTTTCGG<br>GTCGTTGGCTGGTGGGTAGCGGTCTGGTCGTTACCGCGATTGTGGAAATG<br>GTGTCA

References

1. Chen, Y. L.; Zhao, J.; Liu, W.; Gao, J. F.; Tao, L. M.; Pan, H. X.; Tang, G. L., Identification of phoslactomycin biosynthetic gene clusters from Streptomyces platensis SAM-0654 and characterization of PnR1 and PnR2 as positive transcriptional regulators. *Gene* **2012,** *509* (2), 195-200.
2. Vagstad, A. L.; Bumpus, S. B.; Belecki, K.; Kelleher, N. L.; Townsend, C. A., Interrogation of global active site occupancy of a fungal iterative polyketide synthase reveals strategies for maintaining biosynthetic fidelity. *Journal of the American Chemical Society* **2012,** *134* (15), 6865-77.
3. Koryakina, I.; Kasey, C.; McArthur, J. B.; Lowell, A. N.; Chemler, J. A.; Li, S.; Hansen, D. A., Inversion of Extender Unit Selectivity in the Erythromycin Polyketide Synthase by Acyltransferase Domain Engineering. **2017,** *12* (1), 114-123.
4. Kushnir, S.; Sundermann, U.; Yahiaoui, S.; Brockmeyer, A.; Janning, P.; Schulz, F., Minimally Invasive Mutagenesis Gives Rise to a Biosynthetic Polyketide Library. *Angewandte Chemie International Edition* **2012,** *51* (42), 10664-10669.
5. Del Vecchio, F.; Petkovic, H.; Kendrew, S. G.; Low, L.; Wilkinson, B.; Lill, R.; Cortes, J.; Rudd, B. A.; Staunton, J.; Leadlay, P. F., Active-site residue, domain and module swaps in modular polyketide synthases. *J Ind Microbiol Biotechnol* **2003,** *30* (8), 489-94.
6. Vogeli, B.; Geyer, K.; Gerlinger, P. D.; Benkstein, S.; Cortina, N. S.; Erb, T. J., Combining Promiscuous Acyl-CoA Oxidase and Enoyl-CoA Carboxylase/Reductases for Atypical Polyketide Extender Unit Biosynthesis. *Cell Chem Biol* **2018,** *25* (7), 833-839.e4.
7. Sundermann, U.; Bravo-Rodriguez, K.; Klopries, S.; Kushnir, S.; Gomez, H.; Sanchez-Garcia, E.; Schulz, F., Enzyme-directed mutasynthesis: a combined experimental and theoretical approach to substrate recognition of a polyketide synthase. *ACS Chem Biol* **2013,** *8* (2), 443-50.
